# Bronchoalveolar Lavage Proteomics in Patients with Suspected Lung Cancer

**DOI:** 10.1038/srep42190

**Published:** 2017-02-07

**Authors:** Ana Sofia Carvalho, Célia Marina Cuco, Carla Lavareda, Francisco Miguel, Mafalda Ventura, Sónia Almeida, Paula Pinto, Tiago Tavares de Abreu, Luís Vaz Rodrigues, Susana Seixas, Cristina Bárbara, Mikel Azkargorta, Felix Elortza, Júlio Semedo, John K. Field, Leonor Mota, Rune Matthiesen

**Affiliations:** 1 Computational and Experimental Biology Group, Health Promotion and Chronic Diseases Department, National Institute of Health Dr Ricardo Jorge, Lisbon, Portugal; 2 Computational and Experimental Biology Group, CEDOC, Faculdade de Ciências Médicas, Universidade Nova de Lisboa, Lisboa, Portugal; 3 Unidade de Técnicas Invasivas Pneumológicas, Pneumologia II, Hospital Pulido Valente, Centro Hospitalar Lisboa Norte, Lisbon, Portugal; 4 Instituto de Saúde Ambiental, Faculdade de Medicina da Universidade de Lisboa, Lisbon, Portugal; 5 Department of Pulmonology, Unidade Local de Saúde da Guarda, Faculty of Health Sciences, University of Beira Interior, Portugal; 6 Instituto de Investigação e Inovação em Saúde, Universidade do Porto (I3S), Porto, Portugal; 7 Institute of Molecular Pathology and Immunology of the University of Porto (IPATIMUP), Porto, Portugal; 8 Proteomics Platform, CIC bioGUNE, CIBERehd, ProteoRed-ISCIII, Bizkaia Science and Technology Park, Derio, Spain; 9 Roy Castle Lung Cancer Research Programme, Department of Molecular and Clinical Cancer Medicine, University of Liverpool, William Duncan Building, 6 West Derby Street, Liverpool, L7 8TX, UK

## Abstract

Lung cancer configures as one of the deadliest types of cancer. The future implementation of early screening methods such as exhaled breath condensate analysis and low dose computed tomography (CT) as an alternative to current chest imaging based screening will lead to an increased burden on bronchoscopy units. New approaches for improvement of diagnosis in bronchoscopy units, regarding patient management, are likely to have clinical impact in the future. Diagnostic approaches to address mortality of lung cancer include improved early detection and stratification of the cancers according to its prognosis and further response to drug treatment. In this study, we performed a detailed mass spectrometry based proteome analysis of acellular bronchoalveolar lavage (BAL) fluid samples on an observational prospective cohort consisting of 90 suspected lung cancer cases which were followed during two years. The thirteen new lung cancer cases diagnosed during the follow up time period clustered, based on liquid chromatography-mass spectrometry (LC-MS) data, with lung cancer cases at the time of BAL collection. Hundred and thirty-tree potential biomarkers were identified showing significantly differential expression when comparing lung cancer versus non-lung cancer. The regulated biomarkers showed a large overlap with biomarkers detected in tissue samples.

According to the most recent estimates held by the International Agency for Research on Cancer (Globocan 2012) 1.82 million new cases were estimated for lung cancer in 2012^1^. With an overall ratio of mortality to incidence of 0.87, lung cancer leads as one of the deadliest cancers worldwide and specifically among women in more developed regions. In USA, five year survival rate for lung cancer patients is 15–17%^2^. In Europe approximately ninety percent of patients presenting advanced or metastatic stage IIIB/IV will die within five years while eighty percent, die within one year from being diagnosed^3^,^4^. The high fatality associated with the disease is due to late detection and limited performance of the current modalities in diagnosing lesions originating in peripheral, submucosal and peribronchial regions^5^. Screening for lung cancer with low-dose computed tomography (CT) or chest radiography attempts to detect the disease in an early stage. Screening trial initiatives took place in USA and Europe, *e.g.* NELSON trial, demonstrated a considerable increase in the number of early-stage lung cancers diagnosed with low-dose CT^6^. In USA low-dose CT screening for lung cancer has been implemented while in Europe awaits for decision^7^.

Patients with chest radiographic abnormality suspicious of lung cancer often undergo fiberoptic bronchoscopy for diagnosis which is normally confirmed histologically by biopsy and/or cytologically by bronchial washings, bronchial brushings or BAL. In endoscopically visible lung malignancies the diagnosis yield is high and significantly increases if bronchial washings and brushings (both cytology-based sampling techniques) are included when compared with forceps biopsy only^8^. Nevertheless in more than 40% of the cases bronchoscopy is non-diagnostic^9^ despite the development of new technologies such as radial endobronchial ultrasound (EBUS)^10^. In non-visible endoscopically lesions, such as those localized in the peripheral lung, transbronchial lung biopsy (TBLB) is usually performed and the sensitivity depends on the number of biopsy specimens taken and the size of the lesion^11^. The diagnostic rate of TBLB can be further improved if combined with BAL cytology^12^. Even though BAL cytology alone for lung cancer diagnosis has a low sensitivity (range 29–69%) the specificity is very high (90–100%)^12^,^13^,^14^. BAL obtained by fiberoptic bronchoscopy assures a unique representation of the molecular and cellular components from the peripheral airspaces and small airways (airway epithelial and immune cells) as well as the extracellular lining fluid (ELF), and consists of soluble molecules such as phospholipids, proteins, peptides, nucleic acids and cells^4^. BAL components have been explored beyond cytology such as for DNA alterations, e.g. indel, mutations and or methylation, as biomarkers for lung cancer^14^,^15^,^16^. The screening of K-ras mutations in BAL cells in which cytological examination was negative aids the diagnosis of lung cancer patients^17^. Sensitivity of BAL for lung cancer diagnosis can be increased by exploring BAL analysis differently such as by mass spectrometry based proteomics. Upon BAL analysis, such as cell count, culture and cytology, the excess is discarded. Therefore excess BAL specimens discarded after cell collection can be utilized and can add to the diagnosis of non-visible malignancies. Analysis of BAL proteome proved to be less cumbersome compared with other body fluids such as serum or plasma since depletion of the most abundant proteins is not a requirement. Furthermore, BAL is in most cases in direct contact with the tumor in lung cancer patients in contrast to other body fluids. BAL proteomics analysis may have a clinical utility in the diagnostic evaluation of patients with suspected lung cancer who lack an undefined diagnosis despite of chest radiographic abnormality or to prioritize patients for follow up and surgery. Mass spectrometry-based proteomics analysis of BAL has been explored for the study of lung diseases such as idiopathic pulmonary fibrosis^18^, sarcoidosis^19^, chronic obstructive pulmonary disease (COPD)^20^ or cystic fibrosis^21^. The proof of principle for using BAL proteome analysis for lung cancer diagnosis was reported by Oumeraci *et al*. in which six BAL samples from non small cell lung cancer (NSCLC) patients were analyzed using a MALDI-TOF mass spectrometry based method^22^. Following, Pastor *et al*. used 2D-PAGE and MALDI-TOF/TOF to evaluate the protein profile of BAL samples of sixty COPD and NSCLC patients. Forty proteins were found to be significantly differentially regulated between the disease groups and the controls. Seven of these proteins were found by Pastor *et al*. to be specific for lung cancer (CTSD, ALDO A, FBP1, ERZ, AKR1B10, TKT and SELENBP1)^23^. Applying the same 2D-PAGE MS-based technology in a biomarker discovery phase and validation phase a panel comprising APOA1, CO4A, CRP, GSTP1, and SAMP was elected^24^. Almatroodi and co-workers analyzed BAL cells isolated from eight lung adenocarcinoma and eight control patients by liquid chromatography mass spectrometry (LC-MS) and found 33 proteins consistently over expressed^25^. Ortea *et al*. utilized a SWATH DIA MS-based quantitative proteomics approach to analyze BAL from ten controls and twelve patients with lung adenocarcinoma^26^. They found a total of 44 significantly regulated proteins when combining the result from two statistical approaches. More importantly their first principal component of the data demonstrated almost perfect discrimination power between adenocarcinoma and control except for a single control sample.

In view of the discrimination power of the BAL proteome, we explore the BAL proteome on an observational cohort representative of patients with suspected lung cancer undergoing diagnostic bronchoscopy. To this end we performed high resolution LC-MS on 90 patients for which the diagnosis was based on BAL cytology and tumor histology whenever possible (suspicious = 7, no lung cancer suspicion = 47, lung cancer = 36) which were followed for two years (suspicious = 2, no lung cancer suspicion = 39 and lung cancer = 49). Subsets of samples were analyzed by mass spectrometry based proteomics repeatedly at different time points during the study, to demonstrate reproducibility of the applied method, leading to a data set of 124 LC-MS runs.

## Methods

### Study participants

BAL samples were collected prospectively from patients with suspected lung cancer undergoing diagnostic bronchoscopy at the Unidade de Técnicas Invasivas Pneumológicas, Pneumologia II, Hospital Pulido Valente, Centro Hospitalar Lisboa Norte. Human Ethics approval was received from Centro Hospitalar Lisboa Norte and National Health Institute Dr. Ricardo Jorge Research Ethics Committees. All methods were performed in accordance with the relevant guidelines and regulations. Informed consent of all participants was obtained. A total number of 91 BAL samples were collected between April and July 2014 of which 90 were further analyzed by MS. The sample excluded was from a HIV positive patient due to safety concerns. Patient diagnosis were obtained by BAL cytology and whenever available biopsy followed by histology. Patient diagnosis was assessed in two different dates August 2014 and June 2016 (Table 1). A subset of patients missed follow-up appointments or was followed in other hospitals. Non lung cancer patients were diagnosed with diseases such as COPD, interstitial lung disease, bronchiectasis, heart failure, asthma, chronic cough and pulmonary nodule follow up. After the initial bronchoscopy, negative “Suspicious” patients were approached in the following way: a subset of the patients underwent CT-guided transthoracic needle biopsy, others repeated bronchoscopy and some of them were submitted to thoracic surgery. In the remaining of patients presenting with small lung nodules, follow-up was performed according to Fleischner Society Guidelines^27^. The mean time length to the diagnosis of lung cancer was 100.61 days after the first bronchoscopy.

### BAL processing

In most cases, BAL was targeted toward affected lung segments. The procedure was performed by wedging the bronchoscope in a subsegmental bronchus. Usually, three lavages were performed using approximately 50 mL of 0.9% saline solution per lavage. The recovered fluid was placed at 4 °C immediately. BAL was centrifuged at 320× g for 10 minutes at 4 °C, to remove the cellular fraction. The resulting cell-free supernatant was immediately aliquoted and frozen at −80 °C until further analysis.

### Protein sample preparation

BAL samples were distributed to five sample batches by stratified random sampling, with approximately uniform distribution of lung cancer cases in each batch. Subsequent analysis confirmed a fairly even distribution of non-lung cancer versus lung cancer cases in each of the batches. BAL proteins were precipitated with six volumes of ice-cold acetone and then incubated overnight at −20 °C. The samples were then centrifuged at 14,000× g for 20 min at 4 °C and the supernatant discarded. The pellets were washed with ice-cold acetone and then centrifuged at 14,000× g for 20 min at 4 °C and the supernatant discarded. The resultant pellets were dissolved in 100 μl of 8 M urea and 100 mM HEPES with alternate sonication and vortexing. Protein estimation was carried out using the micro BCA assay kit and concentration was adjusted to 100 μg/100 μl using 8 M urea and 100 mM HEPES buffer.

### Peptide sample preparation

Protein solution containing SDS and DTT were loaded onto filtering columns and washed exhaustively with 8M urea in HEPES buffer^28^. Proteins were reduced with DTT and alkylated with IAA. Protein digestion was performed by overnight digestion with trypsin sequencing grade (Promega).

### LTQ Orbitrap XL analysis

Peptide separation was performed on a nanoACQUITY UPLC System (Waters) on-line connected to an LTQ Orbitrap XL mass spectrometer (Thermo Electron). An aliquot of each sample was loaded onto a Symmetry 300 C18 UPLC Trap column (180 μm × 20 mm, 5 μm, Waters). The precolumn was connected to a BEH130 C18 column (75 μm × 200 mm, 1.7 μm, Waters), and equilibrated in 3% acetonitrile and 0.1% FA. Peptides were eluted directly into the LTQ Orbitrap XL mass spectrometer through a nanoelectrospray capillary source (Proxeon Biosystems), at 300 nl/min and using a 120 min linear gradient of 3–40% acetonitrile, followed up by an increase to 40% acetonitrile for the next 30 min. The mass spectrometer automatically switched between MS and MS/MS acquisition in DDA mode. Full MS scan survey spectra (m/z 400–2000) were acquired in the Orbitrap with mass resolution of 30000 at m/z 400. After each survey scan, the six most intense ions above 1000 counts were sequentially subjected to collision-induced dissociation (CID) in the linear ion trap. Precursors with charge states of 2 and 3 were specifically selected for CID. Peptides were excluded from further analysis during 60 s using the dynamic exclusion feature.

#### Preprocessing of MS data

Orbitrap data were calibrated using polycyclodi-methylsiloxane (PCMs—outgassed material from semiconductors) present in the ambient air and Bis(2-Ethylhexyl)(Phthalate) (DEHP—from plastic)^29^,^30^ by modular VEMS^31^. Modular VEMS further allows alternative parent ion annotations for each MS/MS spectrum which is needed if two peptide elution profiles overlap in the m/z and retention time dimension. By allowing alternative parent ion annotation for each MS/MS spectrum, provides a storage space efficient data format. Furthermore, these alternative parent ion annotations were taken into account during the database dependent search.

#### Database search

The obtained data from the 124 LC-MS runs were searched using VEMS^32^,^33^ and MaxQuant^34^. A standard human proteome database from UniProt (3AUP000005640) for which fusion proteins from Sun *et al*.^32^ and previous cancer mutations associated to adenocarcinomas from the COSMIC database^35^ were included. Permutated protein sequences, where Arg and Lys were not permutated, were included in the database. Trypsin cleavage allowing a maximum of 4 missed cleavages was used. Carbamidomethyl cysteine was included as fixed modification. Methionine oxidation, N-terminal protein acetylation, deamidation of asparagine and glutamine was included as variable modifications. 5 ppm mass accuracy was specified for precursor ions and 0.5 m/z for fragment ions. The false discovery rate (FDR) for protein identification was set to 1% for peptide and protein identifications. No restriction was applied for minimal peptide length for VEMS search. Identified proteins were divided into evidence groups as defined by Matthiesen *et al*.^36^.

#### Quantitative analysis

Quantitative data from MaxQuant and VEMS were analyzed in R statistical programming language. Peptide LFQ, peptide spectral counts, protein LFQ, IBAQ and protein spectral counts from the two programs were preprocessed by three approaches: 1) removing common MS contaminants followed by log2(x + 1) transformation, 2) removing common MS contaminants followed by log2(x + 1) transformation and quantile normalization, 3) removing common MS contaminants followed by log2(x + 1) transformation, quantile normalization and abundance filtering to optimize overall Gaussian distribution of the quantitative values. This lead to six quantitative matrices for each of the five different types of quantitative values which were all subjected to statistical analysis utilizing R package limma^37^ where contrast between lung cancer and non-lung cancer was specified. Correction for multiple testing was applied using the method of Benjamini & Hochberg^38^. The result from PCA analysis was robust across the 30 different quantitative matrices created. The presented PCA results in the manuscript are from VEMS spectral counts that was preprocessed by removing common MS contaminants followed by log2(x + 1) transformation, quantile normalization and abundance filtering to optimize overall Gaussian distribution of the quantitative values. For defining potential biomarkers differentiating lung cancer versus non-lung cancer, based on estimated iBAQ values, only results that concur between MaxQuant and VEMS analysis were maintained and presented within this manuscript (see result section for more details).

#### Functional analysis

Details of how we define functional enrichment based on the hypergeometric probability test have previously been described^39^,^40^. Functional enrichment was made by extracting all functional categories for which at least one of the samples showed a significant enrichment based on the hypergeometric probability test. For these categories the number of proteins matching the functional category was extracted and these were compared by a Chi Square and a two-side Mann-Whitney U-test followed by multiple corrections of P values by the method of Benjamini & Hochberg^38^. For the significant functional categories, based on the Chi square test, the log2 number of proteins per category across tissue samples was visualized in a heatmap for cellular component (CC), biological process (BP), molecular function (MF) and KEGG.

#### Literature mining for potential consensus biomarkers

The significant expressed proteins obtained in our study, based on iBAQ values, were compared with eight similar lung cancer studies from the literature obtained on either BAL^23^,^24^,^26^, BAL cells^25^, plasma^41^ and tumor compared to control tissue^42^,^43^,^44^ (see Table S1). The proposed significant down- and up-regulated proteins were compared based on gene names. We observe that different studies concur better if compared on the gene level rather than based on for example UniProt accession numbers. We speculate that the reason for this observation is that MS data provide insufficient data to accurately resolve protein isoforms and this subsequently results in different MS search engines selecting different protein isoforms. We therefore compare the significant regulated proteins after collapsing to the encoding genes. Next a list of significant proteins that displays the same regulation in at least three independent experiments was selected. This list was finally compared with significant proteins found to be regulated in similar direction when comparing COPD patients with controls^20^,^23^.

## Results

### Characteristics of clinical samples

**Figure 1:**
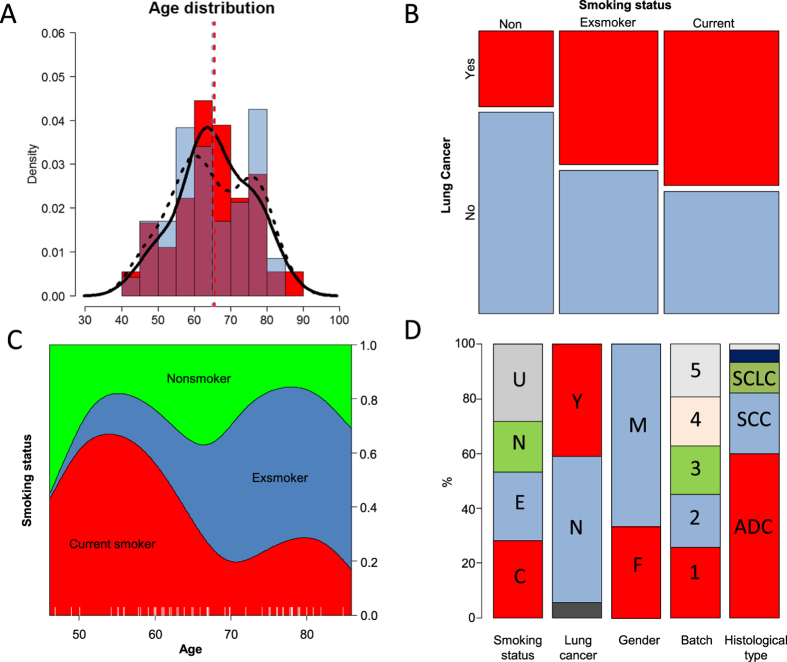
Overview of patients’ characteristics and diagnosis. (**A**) Age distribution between lung cancer cases (red) and non-lung cancer controls (blue). Histogram, density estimate (solid line cases; dotted line controls) and means are depicted for cases (red) and controls (blue). (**B**) mosaic plot of lung cancer versus smoking history. (**C**) Conditional Density plot of smoking history versus age. (**D**) Distribution of smoking history (C: current, E: exsmoker, N: never and U: unknown), cancer (Y: yes, N: no and no letter: lung cancer suspicion), Gender: (F: female, M: male), batch number, type: (ADC: adenocarcinoma, SCC: Squamous cell cancer, SCLC: small cell lung carcinoma and no label: large cell neuroendocrine lung cancer, carcinoid and others).

The baseline characteristics of the clinical samples analyzed in this study are outlined in Table 1. Patients mean age was 65.0 ± 10.5 years (38–86 years), 65.6% were males and 74% of patients with annotated smoking status were current smokers or exsmokers. Figure 1 depicts a subset of the clinical variables of the collected cohort. The mean age is slightly higher in the lung cancer group compared to the non-lung cancer group (Fig. 1A). The age density estimate for non-lung cancer cases displays a bimodal distribution suggesting that this group likely requires further stratification. Current and exsmokers are more represented in the lung cancer group compared to the nonsmokers (Fig. 1B) as expected. The distribution of smoking status change with age displaying fewer current smokers over 65 years (Fig. 1C) which can be explained by longer life expectancy of former and nonsmokers. Figure 1D shows a fairly even distribution for smoking history, cancer diagnostics, gender and batch sizes.

Final diagnoses were based on cytological diagnosis of bronchoalveolar lavage samples and histologic diagnosis based on forceps biopsy samples, whenever available, and were as follows: 57% adenocarcinoma (n = 28), 20% squamous cell carcinoma (n = 10), 8% small-cell carcinoma (n = 4), 2% large cell carcinoma (n = 1), and carcinoid, mixed types and others, (n = 6) which are in concordance with other published clinical cohorts (Table 1). BAL samples for which diagnosis of lung cancer were obtained is subsequently referred to as cases while those patients suspected of lung cancer for which cytology and histology were negative are referred as controls.

### Mass spectrometry analysis of BAL samples

The proteome of ninety BAL samples was analyzed by LC-MS in five different batches of samples, consisting of approximately 25 samples per batch, at different points in time. To assure reproducibility, of sample preparation and LC-MS analysis, we prepared technical replicas analyzed in different batches and therefore at different time points in the study. The hierarchical clustering of the LC-MS data set consisting of 124 LC-MS runs shows that the majority of replicas cluster directly next to each other. The average Euclidian distance for all samples was calculated to ~18 whereas the average distance for replicas was 11. This suggests that both the sample preparation and mass spectrometry analysis were reproducible throughout the analysis procedure. The data was acquired by data dependent acquisition which means protein identifications obtained from the same sample reanalyzed will fluctuate. The Jaccard index can be used to compare the similarity in identified proteins between runs. Figure S1 depicts the distribution of Jaccard index for replica and non-replica samples. The quantification of fluctuation in protein identifications demonstrates that the observed sample differences are not only caused by random fluctuations from the data dependent acquisition. These observations also highlight the necessity to demonstrate that the number of LC-MS runs reaches a reasonable plateau in terms of identifications. The fluctuations in protein identifications have minor effects on the iBAQ quantification since match between runs were used. The total number of identified protein isoforms was 5779 and 2195 when collapsed into encoding genes. Figure S2 depicts how the number of identified protein isoforms and corresponding encoding genes grows as sample size increase. The result in Figs S1 and S2 confirms that BAL proteome can vary significantly from patient to patient. The second sample leads to only a small increase in the number of detected proteins. However, the third sample provides a considerable increase followed by a number of samples with minor contributions to the total number of detected proteins. As the sample size increase further more samples contribute to these plateaus and the raise in detection becomes less pronounced when sample data with new characteristics is included.

### Clustering of BAL LC-MS data

**Figure 2:**
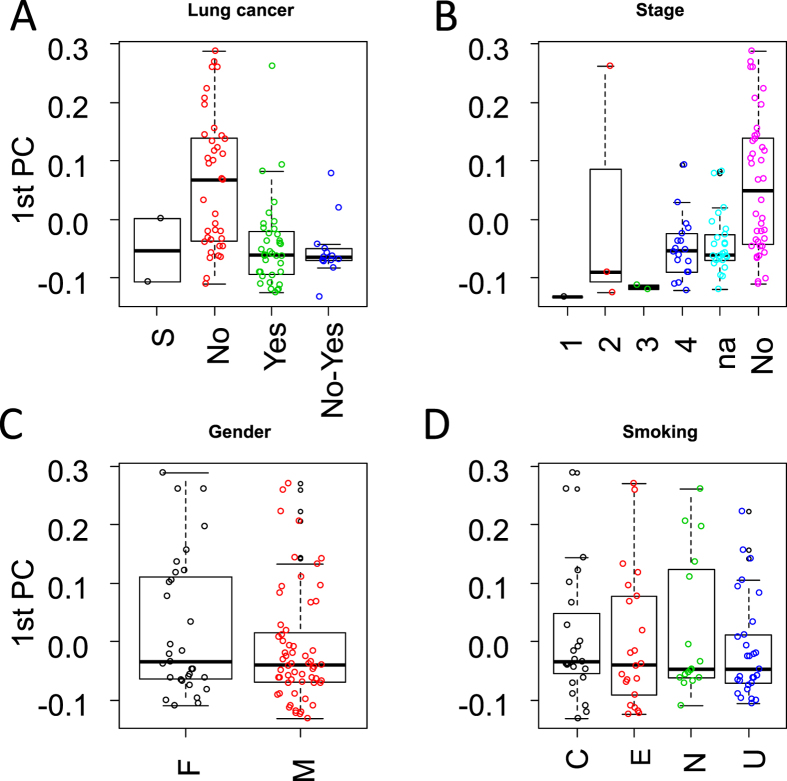
Principal component analysis using spectral count values from all abundant proteins. (**A**) Distribution of values from the first principal component grouped based on patients’ lung cancer status. “No-Yes” indicates the patients that were diagnosed with cancer within the two years of follow up. (**B**) grouped based on patients’ lung cancer staging. (**C**) Grouped based on patients’ gender. (**D**) Grouped based on patients’ smoking status.

The variance captured by mainly the first principal component, from a principal component analysis using spectral count values from abundant detected proteins, correlated mainly with cases and controls in comparison to other tested clinical parameters (Figs 2 and S3). The boxplot of the first principal component, in Fig. 2, is annotated according to the groups: “non-lung cancer” (No), “lung cancer” (Yes), “suspicious” (S) and “suspicious and non-lung cancer which two years later have been diagnosed with lung cancer” (No-Yes). The scatter plot of the first two principal components, in Fig. S3A, shows that the second principal component contains little if any information about lung cancer status. Those patients who died within the two years of follow-up are indicated in black (Fig. S3A). A large number of controls clustered with cases based on the diagnosis at the time of the BAL collection (suspicious = 7, no lung cancer suspicion = 47, lung cancer = 36). Given the low sensitivity of current BAL based diagnosis, we have collected the information available from patients that have been followed up, two years after BAL collection (suspicious = 2, no lung cancer suspicion = 39 and lung cancer = 49). Thirteen of the patients, which at the time of BAL sampling, were diagnosed without lung cancer or had a suspicious diagnosis, had within the two years follow up been diagnosed with lung cancer. These thirteen cases clustered with the lung cancer cases (Figs 2A and S3A). If we assume that 18 non-lung cancer cases are separated from lung cancer samples, based on the first two principal components, out of the 54 non-lung cancer and suspicious lung cancer cases, the probability of picking a separated non-lung cancer is 18/54 = 0.33. To not select any separated non-lung cancer cases in thirteen attempts can be estimated as (1–0.33)^13^ = 0.005. This result further suggests that the first principal component contain clinical applicable prediction value in a clinical representative cohort. A result that likely can be optimized by improving the experimental accuracy of the quantitative methods, improving the sample size and the clinical annotation of the cases. Additionally, we observed one lung cancer case clustering with non-lung cancer controls (Figs 2A and S3A). This corresponds to a patient whose BAL cytology based diagnosis was negative and a more invasive diagnostic methodology, in this case surgery, revealed a peripheral tumor, classified as stage 2, T2N0M0. Other parameters such as cancer stage (Figs 2B and S3B), gender (Figs 2C and S3C) and smoking status (Figs 2D and S3D) showed no correlation to the first two principal components. Correlation between the first two principal components and cancer histology type was not observed. The results obtained were robust regarding the software used (VEMS and/or MaxQuant) and whether spectral counting, MaxQuant LFQ values or iBAQ values were utilized. Finally the distributions observed in Fig. 2 are narrower for lung cancer groups providing further evidence that the variance in the first principal component is caused by cancer versus non cancer cases. Figure S3B shows that most of the patients that died within the first two years were stage IV lung cancer. Of the patients diagnosed with stage IV lung cancer 61% died within the two years follow up period.

### Significant regulated BAL proteins and peptides

**Figure 3:**
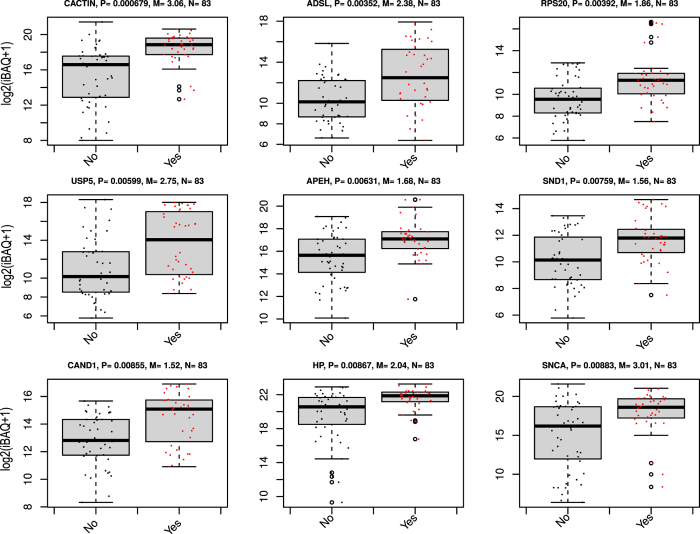
Boxplot of iBAQ expression values for the nine most significant regulated proteins between lung cancer cases and non cancer controls.

LC-MS data set was analyzed by search engines MaxQuant and VEMS using spectral count, label-free quantitation (LFQ) intensities and intensity-based absolute quantitation (iBAQ) values for quantitative statistical analysis. The results obtained from MaxQuant and VEMS iBAQ values were combined in the following way: 1) Statistical analysis from MaxQuant and VEMS output were filtered to select proteins significantly regulated after correction for multiple testing by the method of Benjamini & Hochberg (p < 0.05), 2) significantly regulated proteins were further filtered to maintain only proteins that were more than two fold regulated in either the VEMS or MaxQuant results and 3) finally the protein list were filtered to only include proteins that show the same direction of regulation in both MaxQuant and VEMS results (Fig. S4). MaxQuant and VEMS estimated log2 ratios based on iBAQ values of proteins in the consensus list were plotted in a scatterplot (Fig. S4). The analysis strategy described above lead to a robust list of 133 significantly differentially expressed proteins between lung cancer and non-lung cancer samples with a reduced software bias. This group of proteins will be further referred to as the consensus list. The scaled iBAQ values as estimated by VEMS for the consensus list of differential regulated proteins from MaxQuant and VEMS is depicted in Fig. S5. For the proteins in the consensus list the estimated iBAQ values from MaxQuant and VEMS are similar (Fig. S4) and therefore the heatmap based on MaxQuant iBAQ values give similar results. We observed five main clusters of clinical cases. Three of the main clusters mainly consist of non-lung cancer cases whereas the remaining two mainly consist of lung cancer cases. The VEMS iBAQ values for the nine most significant differential expressed proteins when comparing lung cancer versus non-lung cancer in the consensus list are represented in Fig. 3 (Boxplots for all the significant differential expressed proteins in the MaxQuant and VEMS consensus list is provided in Fig. S6).

VEMS iBAQ expression values of proteins in the consensus list were depicted in boxplots according with lung cancer histology types (Fig. S7). There were in general few differences in the distribution of iBAQ values across histological types for the consensus list of proteins (see Fig. S7). The histology group “SCC + ADC” consisting of a single sample case displays for some of the proteins iBAQ values more similar to the non-lung cancer cases. This lung cancer case is the above discussed peripheral lung cancer (stage 2 peripheral tumor, T2N0M0, for which the BAL based diagnosis was negative and lung cancer diagnosis was only obtained after surgery). Fig. S8 summarize proteins which were only identified in cancer samples based on the clinical annotation in 2014. The proteins in Fig. S8 are potentially markers for specific sub types of cancer and therefore of interest in a follow up study. Most of these proteins were only identified in few samples and consequently will come out as non-significant when comparing lung cancer versus controls. Nevertheless, some of these proteins have previously been described to be upregulated in cancer such as the kinetochore protein NDC80 homolog and the proteasome factors^45^.

Heatmap of mziXIC values for abundant peptides stratifies the samples in two main clusters and 7–8 sub clusters (see Fig. S9). Two of the sub clusters are uniform with respect to either lung cancer or non-lung cancer (see Fig. S9, highlighted). Peptide quantitative values provided a similar separation based on the first two principal components as observed for protein quantitative values (not shown).

### Functional analysis of BAL proteins

**Figure 4:**
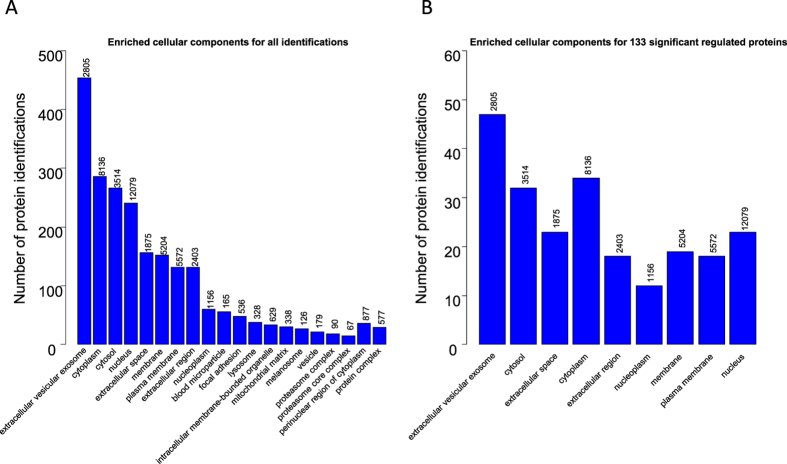
GO enrichment analysis (cellular component) of all identified proteins (**A**) and significant regulated proteins between cases and controls (**B**). The y-axis indicates the number of proteins in each cellular component category for all identified proteins and for the significantly regulated proteins. The numbers on top of the columns indicates the number of proteins in each category in total.

Functional enrichment and regulation analysis (see methods for details) using gene ontology (GO) categories cellular component (CC), molecular function (MF) and biological process (BP) revealed a number of functional groups for which protein enrichment was significantly different between the ninety BAL samples (Figs S10 and 11, CC; S12–13, MF; and S14–15, BP). Proteins in the main GO cellular component terms such as cytoplasm/cytosol, nucleus, extracellular space, plasma membrane/membrane and extracellular vesicular exosomes (EVEs) were consistently identified and enriched for all samples. This was the case for all the identified proteins (Fig. S10) and also for the proteins in the consensus list (Fig. S11) when analyzed by functional enrichment. EVE proteins were the most enriched when comparing relative enrichment estimates, P values and number of identified proteins (Fig. 4A and B).

The abundance of circulating exosomes is known to be elevated in an extensive range of diseases^46^. We consequently explored if there was an overall quantitative difference in exosome proteins when comparing total spectral counts from lung cancer versus non-lung cancer patient samples (Fig. S16). However, no significant difference in total spectral counts for exosome proteins was found. To further explore and provide evidence for abundant BAL exosomes, protein encoding genes identified and annotated to major subcellular localizations were compared by a Venn diagram (Fig. S17). Therefore targeting at specific identified encoding proteins across five major cellular component categories, EVE proteins were still the most identified and most significantly enriched. This observation was further explored by calculating the mean log2(iBAQ + 1) values for proteins specific for each of the five major cellular components, as an estimate of overall abundance, and compared in Fig. S18. According to the mean log2(iBAQ + 1) values proteins specifically annotated to extracellular space were the most abundant followed by specific EVE proteins. Specific proteins from extracellular space and EVE were 4–16 more abundant than specific proteins from cytoplasm, nucleus and membrane (Fig. S18 – note log2 scale). Proteins belonging to the molecular function category “ATP binding” were observed as being consistently enriched across samples (Figs S12 and 13). For the biological process categories proteins involved in “small molecule metabolic process”, “GTPase activity” and “viral process” were most enriched (Figs S14 and 15). The biological processes “gene expression”, “viral proteins” and “small molecule metabolic process” were most enriched for the significant regulated proteins between cancer and non-cancer cases Fig. S19).

## Discussion

Early cancer diagnosis is a major challenge particularly in lung cancer for which incidence and mortally rates are extremely high worldwide^1^. Clinical proteomics studies using biofluids can contribute to improve detection and stratification of tumors as well as prediction of efficacy in therapeutic treatments. Ninety BAL fluid samples of patients stratified into 49 cases, 39 controls and 2 undefined were analyzed by high resolution mass spectrometry. A large number of differentially regulated proteins (133) were identified of a total number of 5779 protein isoforms and 2195 when collapsed into encoding genes. Based on the shape of the number of identified proteins as a function of the number of samples, a reasonable and representative sampling of the BAL proteome was obtained. A significant increase in the number of samples will be required to obtain a substantial higher coverage (Fig. S1). We envisage that this analysis can be used to predict the relation between sample size and number of identified proteins in clinical proteomics studies using BAL. This correlation must considered MS instruments sensitivity though.

The principal component analysis of protein expression values shows that the variance captured by the first principal components is mainly associated with lung cancer (Fig. 2A) in accordance with Ortea *et al*.^26^ which compared adenocarcinoma cases and control BAL fluid patient samples (n~22). Although we have obtained similar results to Ortea *et al*.^26^, we additionally demonstrated that this type of separation succeeds for a cohort data set that reflects the heterogeneity of patient disease characteristics in a clinical setting with a larger variation in histological types. The two main principal components based on measured protein expression values showed no association to gender, batch effects or smoking status (see Figs 2 and S3). A large number of non-lung cancer cases grouped with lung cancer cases based on the diagnostics obtained at the time of BAL fluid sample collection (2014). Previous results have shown that the BAL based cytology diagnosis fail to detect up to 50% of the positive cases^8^ which could explain the large number of controls that cluster with lung cancer. This is reinforced by the fact that of the thirteen cases that changed diagnostic status from 2014 to 2016 all were non-lung cancer cases or suspicious for lung cancer which based on the MS data cluster with lung cancer cases (Figs 2A and S3A). We demonstrated that this is unlikely to be a random result and considering that ~ 25% of the patients’ follow up information was not available in this study the clinical impact is likely more eminent in future validation studies. We speculate that patient stratification based on BAL proteome analysis can be optimized to prioritize patients for a restrict follow-up and/or surgery. Due to the future implementation of early screening methods such as exhaled breath condensate analysis and low dose CT, an increase burden on bronchoscopy units is foreseen. Therefore, we believe BAL based proteomics analysis will have a clinical impact in the future. Furthermore, a patient with a negative diagnosis for lung cancer in 2014 based on bronchoscopy diagnosis, although based on MS data clustered with lung cancer cases, after re-evaluation six months after the initial diagnosis a stage IV lung cancer was detected.

PCA analysis clearly showed two main clusters, one consisting mainly of non-lung cancer controls and a second cluster consisting of lung cancer cases. In fact, based on the stratification we obtained in hierarchical clustering (Fig. S7) and principal component plots (Fig. 2A) we speculate that it will be possible to classify at least one third of the non-lung cancer controls at a higher accuracy based on proteome based diagnostic in bronchoscopy units. Additionally it could contribute to ameliorate the psychological impact on those patients with a low probability of lung cancer which would require a less restrict surveillance.

Nevertheless, we observed one lung cancer case grouping with non-lung cancer cases in Fig. 2A which was not possible to diagnose based on the BAL samples presumably due to the peripheral location of the tumor. This defines one of the limitations of BAL based diagnostics. Diverse evidence obtained from example imaging, proteomics and cytology data can conveniently be combined in probabilistic graphical models to improve prediction of clinical outcome. Our results clearly indicate that BAL based proteomics have the potential to improve the sensitive of bronchoscopy based diagnostics especially if combined with results from imaging.

**Figure 5:**
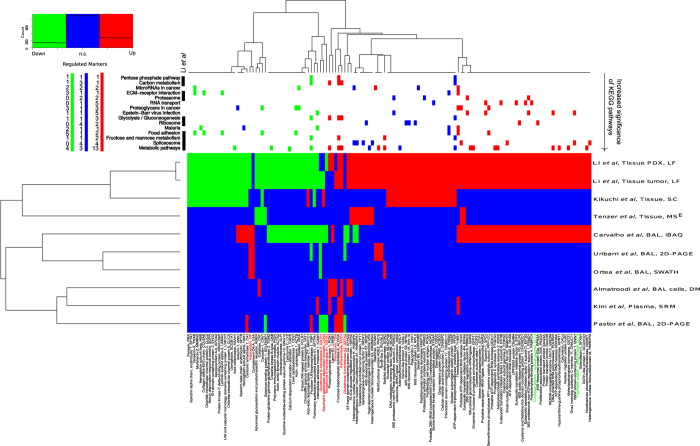
Overview of potential lung cancer biomarkers found to be consistently up- or down-regulated in three or more experiments. The heatmap depicts down-regulated in green, up-regulated in red and non-significant or not detected in blue. Gene and protein names in red indicate that the same gene has been reported regulated in the same direction in COPD. The column annotations on top depict which proteins are annotated to significant enriched KEGG pathways. The colors summarize the consensus regulation in BAL and tissue samples: Green - down in both BAL and tissue, blue conflict regulation in BAL versus tissue and red up regulated in both BAL and tissue samples. Left top panel summarize the overall regulation within significant enriched KEGG pathways. KEGG pathways that concur with Li *et al*. are indicated with black boxes. Abbreviations for quantitative methods are: DM, dimethyl labeling; SRM, single reaction monitoring; MS^E^, MS to E; SC, spectral count; iBAQ, intensity based absolute quantification; SWATH, sequential window acquisition of all theoretical fragment ion spectra.

We have identified 133 proteins which showed significant differential expression comparing cases and controls, in this discovery phase of a multistage biomarker pipeline for management of patients with suspected lung cancer by BAL proteomics analysis. It is well accepted that the best possible validation are performed by independent researchers using alternative methodologies and other sources of clinical samples. We therefore systematically compared the proteins claimed to be differential expressed between non-lung cancer and lung cancer obtained in this study with the significant protein regulation reported in the literature^23^,^24^,^25^,^26^,^41^,^42^,^43^,^44^. Table S1 summarizes the differential expressed proteins between non-lung cancer and lung cancer reported in the literature and the ones obtained in this study. The heatmap in Fig. 5 summaries the potential lung cancer biomarkers proposed in recent MS-based proteomics studies and those proposed in this study that was at least reproducible in three independent experiments (see method section for details).

Studies based on tissue samples cluster together in one main cluster whereas plasma, BAL cells and BAL based studies cluster together in a second main cluster as expected (see Fig. 5). The potential biomarkers that overlap with COPD potential biomarkers were highlighted in red if the proposed direction of protein regulation is the same. Interestingly the significant regulated proteins between lung cancer and non-lung cancer cases proposed in this study shows an extensive overlap with the significant regulated proteins identified in lung cancer tissue. This suggests that a large number of the regulated proteins observed in BAL proteomics analysis in this study likely originate from the tumors. The top panel in Fig. 5 indicates which proteins are identified in significantly enriched KEGG pathways and the proteins’ regulation in BAL versus tissue. Metabolic pathways constituted the most significant KEGG pathway which is consistent with the result reported by Li *et al*.^44^ for lung cancer tissue samples. The majority of dysregulated protein in metabolic pathways were found to be up regulated (Fig. 5). The proteins in metabolic pathways in this study were mainly upregulated (13 proteins versus 4, Fig. 5). Active clinical development of inhibitors targeting metabolic enzymes as anticancer therapeutics is ongoing^47^. These inhibitors could potentially be tagged with fluorescent labels and used for diagnostic purposes in acellular BAL samples. Dysregulation of metabolic proteins constitute a general feature across histological types and this fact is consistent with the separation observed by PCA in Figs 2A and S3A. Several proteins involved in RNA processing, RNA regulation, transport and translation were in KEGG pathways functionally enriched (spliceosome, MicroRNAs in cancer, RNA transport and ribosome). Focal adhesion and ECM-receptor interaction were identified as functional enriched KEGG pathways and the associated proteins were mainly downregulated (Fig. 5). Proteasome factors are known in general to be up regulated in cancer which is in agreement with observed regulation of the functional enriched proteasome KEGG pathway (Fig. 5). There are major efforts to target proteasome factors for cancer therapy and diagnosis^45^ and this topic is one of the major activities in the EU cost action Proteostasis BM1307. In addition, the cytosolic chaperonin complex plays a key role in proteostasis. The CCT chaperonin complex mediates the folding of the major cytoskeletal proteins such as tubulins and actins. The folding of several proteins involved cell proliferation and tumor genesis such as cyclin E, Von Hippel-Lindau tumour suppressor protein, cyclin B and p21ras are also mediated by the CCT chaperonin complex^48^. CCT2, CCT3, CCT4 and CTT5 was found to be significant and more than two fold up regulated in our study which is consistent with the results obtained by Li *et al*.^44^. CCT2 and CCT3 are highlighted in green in Fig. 5. CCT4 and CTT5 are not included in Fig. 5 since the regulation found by Li and co-workers on these factors are not consistently above two fold although just below the two fold threshold used to generate Fig. 5. Li and co-workers in addition found CCT6A, TCP1, CCT7 and CCT8 significantly up regulated which was identified but not significantly up regulated in our study. CCT6A is amplified together with EGFR on chromosome 7^44^. The KEGG pathway “Epstein-Barr virus infection” with the majority of the proteins upregulated supports the idea of exploring the role of virus infections in lung cancer such as human papilloma virus^49^ and Epstein-Barr virus^50^. Alternatively, the infection associated KEGG pathways in Fig. 5 could be due to a general infection response or in case of Epstein-Barr virus, proteins leaked from infected B cells^51^. Six significant regulated BAL proteins annotated to “proteoglycans in cancer” were found to be consistent with the regulation observed in lung cancer tissue samples. Progressive changes in proteoglycans occur in the tumor microenvironment but the role of these changes needs further investigation^52^. Overall the overlap in functional enriched KEGG pathway has a striking similarity to the KEGG pathways reported by Li and co-workers^44^. Ten of the KEGG pathways in Fig. 5 were also highlighted by these authors.

**Figure 6:**
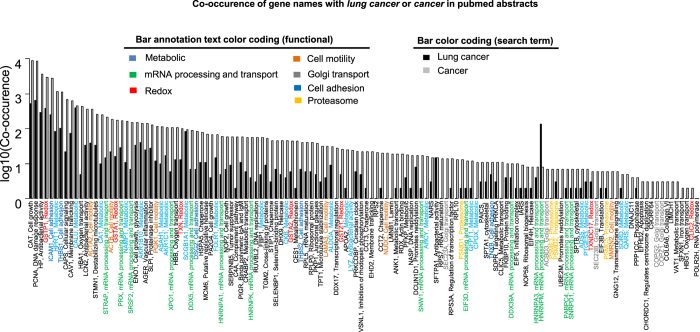
Co-occurrence of potential lung cancer biomarkers found to be consistently up- or down-regulated in three or more experiments with “lung cancer” or “cancer” in PubMed abstracts. Major functional groups are color coded in the bar text annotation.

Majority of the proteins in Fig. 5 which could be reproduced in at least 3 experiments have been associated to lung cancer or cancer in the literature. Figure 6 shows co-occurrence of the corresponding gene names with either *lung cancer* or *cancer* in PubMed abstracts. The proteins in Figs 5 and 6 function in a wide variety of processes. However, mainly in metabolic (N = 21), mRNA processing and transport (N = 14), redox (N = 7), cell motility (N = 6), golgi transport (N = 5), cell adhesion (N = 5) and proteasome (N = 4) processes (Figs 5 and 6). We next compared our consensus list of potential biomarkers with potential biomarkers from several published OMICs studies (Fig. S20). We again observe the biggest overlap with significant regulated proteins identified from lung cancer tissue by Li *et al*.^44^. There is little overlap when comparing genomics, transcriptomics and proteomics data, confirming the observation by Li *et al*.^44^.

Our proposed consensus list contains several novel potential biomarkers for detection of lung cancer. Several of these novel potential biomarkers have been discussed in the literature as potential biomarkers for lung cancer or other cancer types. GO functional analysis of the potential biomarkers are summarized in Figs 4B and S19B.

EVE proteins were demonstrated to be abundant and enriched in BAL fluid samples of lung cancer cases and non-lung cancer controls. Furthermore, ~35% of significant regulated proteins in the consensus list were annotated as EVE proteins. This raise the question if isolating exosomes from BAL before proteomics will lead to more accurate stratification of non-lung cancer versus lung cancer. The diversity of the cell derived exosomes present in BAL fluid samples e.g. immune cells, cancer cells or blood contamination, remains to be resolved. Comparing iBAQ expression of CD molecules from the human leucocytes in non-lung cancer versus lung cancer (http://www.hcdm.org)^53^ revealed no significant difference (Fig. S21). This excludes the possibility that the proposed biomarkers are caused by difference in immune cells in BAL from non-lung cancer versus lung cancer cases. This also makes sense considering that the cells were spun down in the BAL samples within two hours after sample collection. This is also supported by the strong overlap in potential biomarkers between our consensus list of potential biomarkers and the significant markers in lung cancer tissue observed by Li *et al*.^44^. Finally, proteins involved in various bacterial and viral infections were enriched in specific subsets of clinical samples. The clinical impact of this type of sample discrimination needs to be addressed in future studies which will require larger sample sets and thorough clinical annotation of patients.

In conclusion, this study demonstrated that MS-based proteomics of BAL fluid samples provides a valuable source of information for diagnosis purposes that can deliver clinical stratification of samples. Several identified proteins correlated statistical significantly with lung cancer cases. There is now accumulated evidence that LC-MS-based proteomics analysis of a larger and targeted clinical cohort using either antibodies or SRM targeted proteomics for improved sensitivity will lead to the discovery of cost effective proteome based clinical biomarkers. Therefore, diagnostic based on BAL proteomics holds the promise to increase the diagnostic sensitivity for detecting lung cancer. Nevertheless, some peripheral cases will be missed using this strategy alone, one peripheral case out of ninety samples was missed in this study. The acquired LC-MS data should therefore be analyzed in combination with imaging data and additional clinical information as a mean to prioritize patients in terms of disease management. Finally, BAL proteome based diagnostics can potentially be implemented directly in bronchoscopy units based on either chemical probes or antibody methodologies (e.g. by targeting metabolic enzymes or proteostasis factors).

## Additional Information

**How to cite this article**: Carvalho, A. S. *et al*. Bronchoalveolar Lavage Proteomics in Patients with Suspected Lung Cancer. *Sci. Rep.* **7**, 42190; doi: 10.1038/srep42190 (2017).

**Publisher's note:** Springer Nature remains neutral with regard to jurisdictional claims in published maps and institutional affiliations.

## Supplementary Material

Supplementary Figures

Supplementary Table

## Figures and Tables

**Table 1:** Patients’ baseline characteristics of an observational cohort.

Study Population Characteristics	N = 90
**Age group, years**	**2014**
<60	25 (S = 0, No = 12, Yes = 13, P = 0.8/0.8)
60–79	59 (S = 2, No = 25, Yes = 32, P = 0.4/0.6)
80+	6 (S = 0, No = 2, Yes = 4, P = 0.4/0.6)
Mean ± SD	65.1 ± 10.8
**Gender**	**2014**
Male	59 (S = 1, No = 20, Yes = 38, P = 0.02/0.2)
Female	31 (S = 1, No = 11, Yes = 19, P = 0.1/0.4)
**Smoking Status**	**2014**
Nonsmoker	16 (S = 0, No = 9, Yes = 7, P = 0.6/0.8)
Exsmoker	22 (S = 0, No = 7, Yes = 15, P = 0.1/0.4)
Current smoker	23 (S = 1, No = 10, Yes = 12, P = 0.7/0.8)
Unknown	29
**Cytology**	**2014/2016**
Negative	47/39
Positive	36/49
Suspicious	7/2
**Histology**	**2016**
Adenocarcinoma	28
Squasmous cell carcinoma	10
Small cell carcinoma	4
Large cell carcinoma	1
Others	6

Patients’ diagnostic, based on BAL samples, was accessed at two different periods, August 2014 and June 2016. Suspicious, non-lung cancer and lung cancer are indicated by “S”, “No” and “Yes”, respectively. P value was calculated by a chi square test comparing non-lung cancer and lung cancer case. P values are provided before and after correction for multiple testing using FDR.
