# Supplementary material for: Bronchoalveolar Lavage Proteomics in Patients with Suspected Lung Cancer
Source: Sci Rep. 2017 Feb 7;7:42190. doi: 10.1038/srep42190 (PMC5294405; doi:10.1038/srep42190)
Supplement: Supplementary Figures [file srep42190-s1.pdf]

Title Page

Title

Bronchoalveolar Lavage Proteomics in Patients with Suspected Lung Cancer

Authors and Affiliations:

Ana Sofia Carvalho[1,2], Célia Marina Cuco[3], Carla Lavareda[3], Francisco Miguel[3], Mafalda Ventura[3], Sónia Almeida[3], Paula Pinto[3,4], Tiago Tavares de Abreu[3], Luís Vaz Rodrigues[5], Susana Seixas[6,7], Cristina Bárbara[3,4], Mikel Azkargorta[7], Felix Elortza[8], Júlio Semedo[3], John K Field[8], Leonor Mota[3], Rune Matthiesen[1,2]

1. *Computational and Experimental Biology Group, Health Promotion and Chronic Diseases Department, National Institute of Health Dr Ricardo Jorge, Lisbon, Portugal.*
2. *CEDOC, Faculdade de Ciências Médicas, Universidade Nova de Lisboa, Lisboa, Portugal*
3. *Unidade de Técnicas Invasivas Pneumológicas, Pneumologia II, Hospital Pulido Valente, Centro Hospitalar Lisboa Norte, Lisbon, Portugal.*
4. *Instituto de Saúde Ambiental, Faculdade de Medicina da Universidade de Lisboa*
5. *Department of Pulmonology, Unidade Local de Saúde da Guarda, Faculty of Health Sciences, University of Beira Interior, Portugal*
6. *Instituto de Investigação e Inovação em Saúde, Universidade do Porto (I3S), Porto, Portugal;*
7. *Institute of Molecular Pathology and Immunology of the University of Porto (IPATIMUP), Porto, Portugal*
8. *Proteomics Platform CIC bioGUNE, CIBERehd, ProteoRed-ISCIII, Bizkaia Science and Technology Park, Derio, Spain.*
9. *Roy Castle Lung Cancer Research Programme, Department of Molecular and Clinical Cancer Medicine, University of Liverpool, William Duncan Building, 6 West Derby Street, Liverpool, L7 8TX, UK.*

Running Title

BAL proteomics in lung cancer diagnostics

Keywords

Lung Cancer, Bronchoalveolar Lavage, Diagnosis, Proteomics, Biomarkers

Supplementary figure legends

Figure S1. Distribution of Jaccard indexes comparing protein identifications from replica and non-replica samples.

Figure S2. Increase in the number of proteins (black bars) and protein encoding genes (grey bars) identified as sample size increase.

Figure S3. Principal component analysis using spectral count values from all abundant proteins. A) Annotated based on patients' lung cancer status as indicated in the legend. Black symbols indicate patients who died within the two years of follow-up. B) Annotated based on patients' lung cancer staging. Black symbols indicate patients who died within the two years of follow-up. C) Annotated based on patients' gender. D) Annotated based on patients' smoking status.

Figure S4. Comparison of ratios obtained from VEMS and MaxQuant analysis for biomarkers found significant regulated for non-lung cancer versus lung cancer by both software.

Figure S5. Heatmap of significant regulated proteins based on iBAQ values obtained from VEMS and MaxQuant. Left bar depicts lung cancer cases (red) versus non lung cancer controls (green).

Figure S6. Boxplot of iBAQ expression values for all significant regulated proteins between cases and controls in the consensus list. Barplot titles includes gene name, P value correct for multiple testing, M log2 fold ratio and N the sample size.

Figure S7. Boxplot of iBAQ expression values for all significant regulated proteins between cases and controls factored on histology groups. Barplot titles includes gene name, P value correct for multiple testing, M log2 fold ratio and N sample size. The indicated P values results from comparing cases and controls.

Figure S8. Heatmap of protein biomarkers identified exclusively in lung cancer samples. The ten most significantly functional enriched KEGG pathways are indicated in the top of the heatmap where a black bars depicts which functional categories the protein belong.

Figure S9. Heatmap of peptide mziXIC values for most abundant peptides. "Yes" and "No" indicates the patients status with respect to lung cancer. Two sub clusters are highlighted with only lung cancer (light red) and non-lung cancer (light green).

Figure S10. Significantly enriched GO terms within the cellular component category (CC) for all identified proteins across all patient samples. The heatmap depicts the log2 number of proteins identified in different cellular component categories across patient samples.

Figure S11. Significantly enriched GO terms within the cellular component (CC) category for proteins in the consensus list across all patient samples. The heatmap depicts the log2 number of proteins in consensus list and in different cellular component categories across patient samples.

Figure S12. Significantly enriched GO terms within the molecular function (MF) category for all identified proteins across all patient samples. The heatmap depicts the log2 number of proteins identified in different molecular function categories across patient samples.

Figure S13. Significantly enriched GO terms within the molecular function (MF) category for proteins in the consensus list across all patient samples. The heatmap depicts the log2 number of proteins in consensus list and in different functional molecular function categories across patient samples.

Figure S14. Significantly enriched GO terms within the biological process (BP) category for all identified proteins across all patient samples. The heatmap depicts the log2 number of proteins identified in different biological process categories across patient samples.

Figure S15. Significantly enriched GO terms within the biological process category (BP) for proteins in the consensus list across all patient samples. The heatmap depicts the log2 number of proteins in consensus list and in different biological process categories across patient samples.

Figure S16. Boxplot factored on lung cancer versus non-lung cancer cases of total spectral counts for all identified proteins annotated as "extracellular vesicular exosome".

Figure S17. Venn diagram comparing identified proteins in five major cellular component categories.

Figure S18. Barplot of mean log2(iBAQ+1) for proteins specific for one of the major cellular component category in Figure S15. The numbers of the bars indicates the number of specific proteins for each of the major cellular component categories that were utilized for the calculation of mean log2(iBAQ+1) values.

Figure S19. GO enrichment analysis (biological process) of all identified proteins (A) and significant regulated proteins between cases and controls (B). The y-axis indicates the number of proteins in each biological process category for all identified proteins and for the significantly regulated proteins. The numbers on top of the columns indicates the number of proteins in each category in total.

Figure S20. Reproducibility of VEMS/MaxQuant consensus lung cancer biomarkers in other OMICs lung cancer studies published in the literature. The proposed genes are

order with the genes reproduced most times in the top. The heatmap depicts down-regulated in green, up-regulated in red and non-significant or not detected in blue.

Figure S21. Boxplot comparing iBAQ expression of CD molecules from human leucocyte in non-lung cancer versus lung cancer (http://www.hcdm.org). The iBAQ expression values were compared by a Wilcox test. N indicates the number of markers that were identified by MS for the specific Leucocyte.

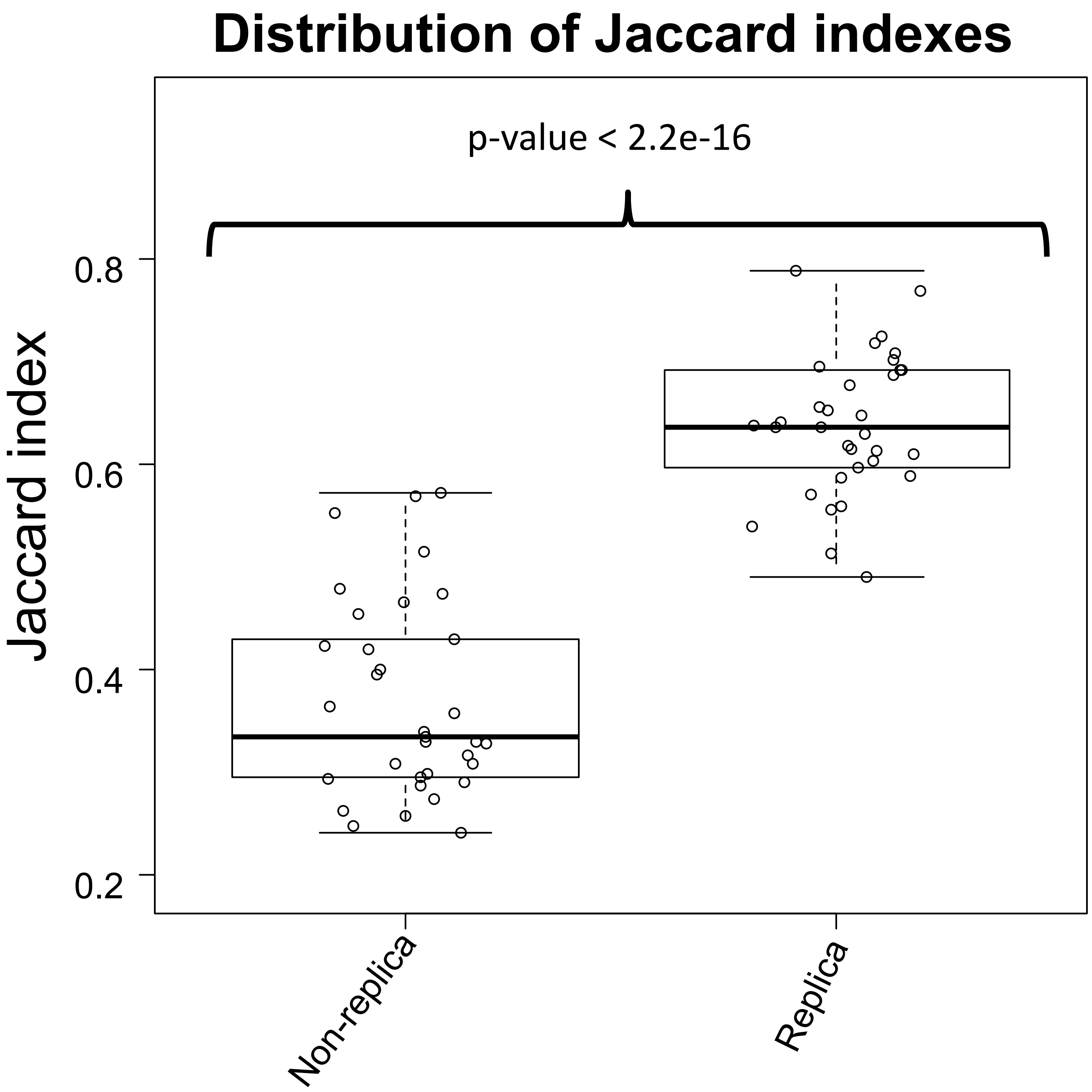

Distribution of Jaccard indexes
p-value < 2.2e-16
Jaccard index
0.8
0.6
0.4
0.2
Non-replica
Replica

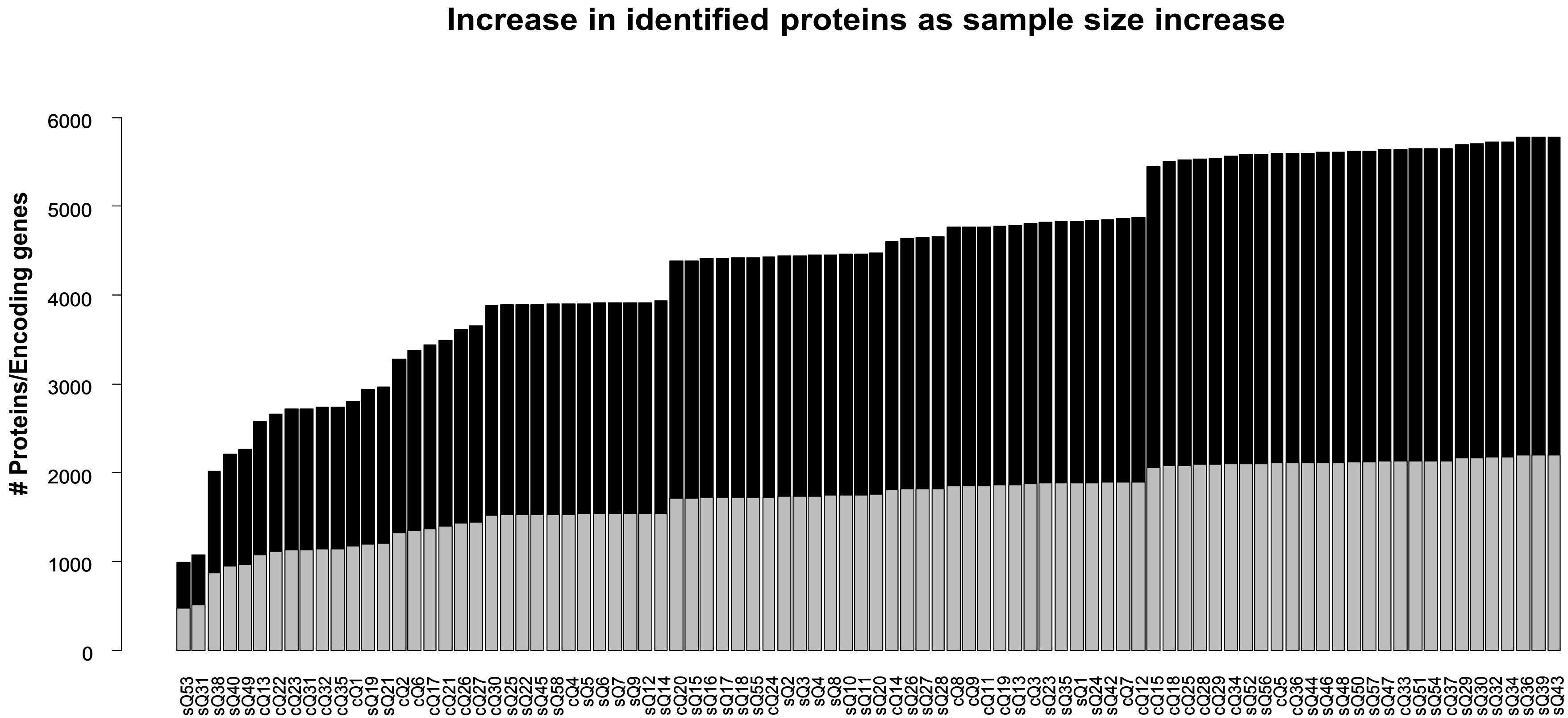
Increase in identified proteins as sample size increase
Proteins/Encoding genes
0
1000
2000
3000
4000
5000
6000
sQ53 sQ31 sQ38 sQ40 sQ49 cQ13 cQ22 cQ23 cQ31 cQ32 cQ35 cQ1 sQ19 sQ21 cQ2 cQ6 cQ17 cQ21 cQ26 cQ27 cQ30 sQ25 sQ22 sQ45 sQ58 cQ4 sQ5 sQ6 sQ7 sQ9 sQ12 sQ14 cQ20 sQ15 sQ16 sQ17 sQ18 sQ55 cQ24 sQ2 sQ3 sQ4 sQ8 sQ10 sQ11 sQ20 cQ14 sQ26 sQ27 sQ28 cQ8 cQ9 cQ11 cQ19 sQ13 cQ3 sQ23 sQ35 sQ1 sQ24 sQ42 cQ7 cQ12 cQ15 cQ18 cQ25 cQ28 cQ29 cQ34 sQ52 sQ56 cQ5 cQ36 sQ44 sQ46 sQ48 sQ50 sQ57 sQ47 cQ33 sQ51 sQ54 cQ37 sQ29 sQ30 sQ32 sQ34 sQ36 sQ39 sQ43

Figure S2

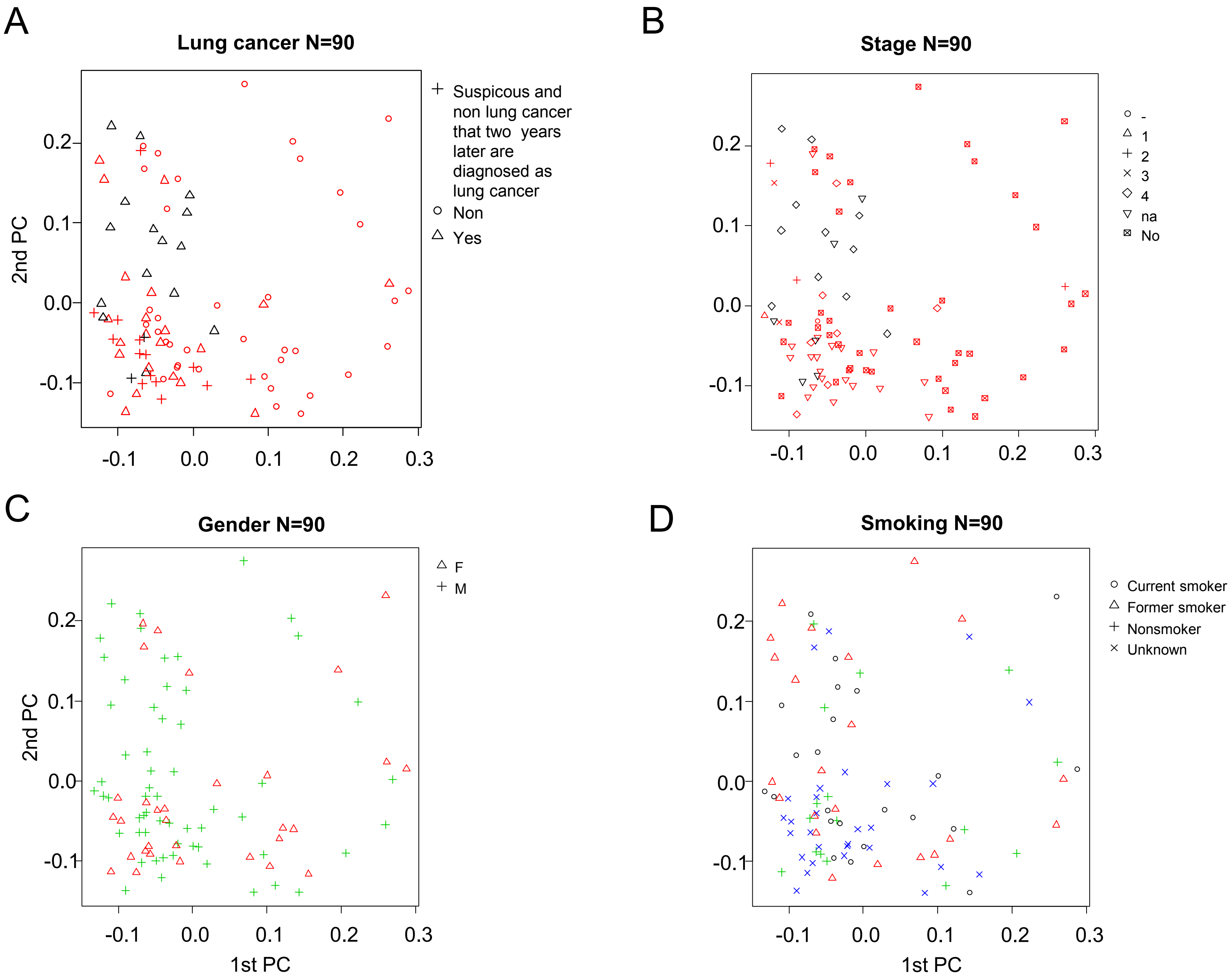
A
Lung cancer N=90
Suspicous and non lung cancer that two years later are diagnosed as lung cancer
Non
Yes
2nd PC
B
Stage N=90
-
1
2
3
4
na
No
C
Gender N=90
F
M
2nd PC
1st PC
D
Smoking N=90
Current smoker
Former smoker
Nonsmoker
Unknown
1st PC
0.2
0.1
0.0
-0.1
-0.1
0.0
0.1
0.2
0.3

Figure S3

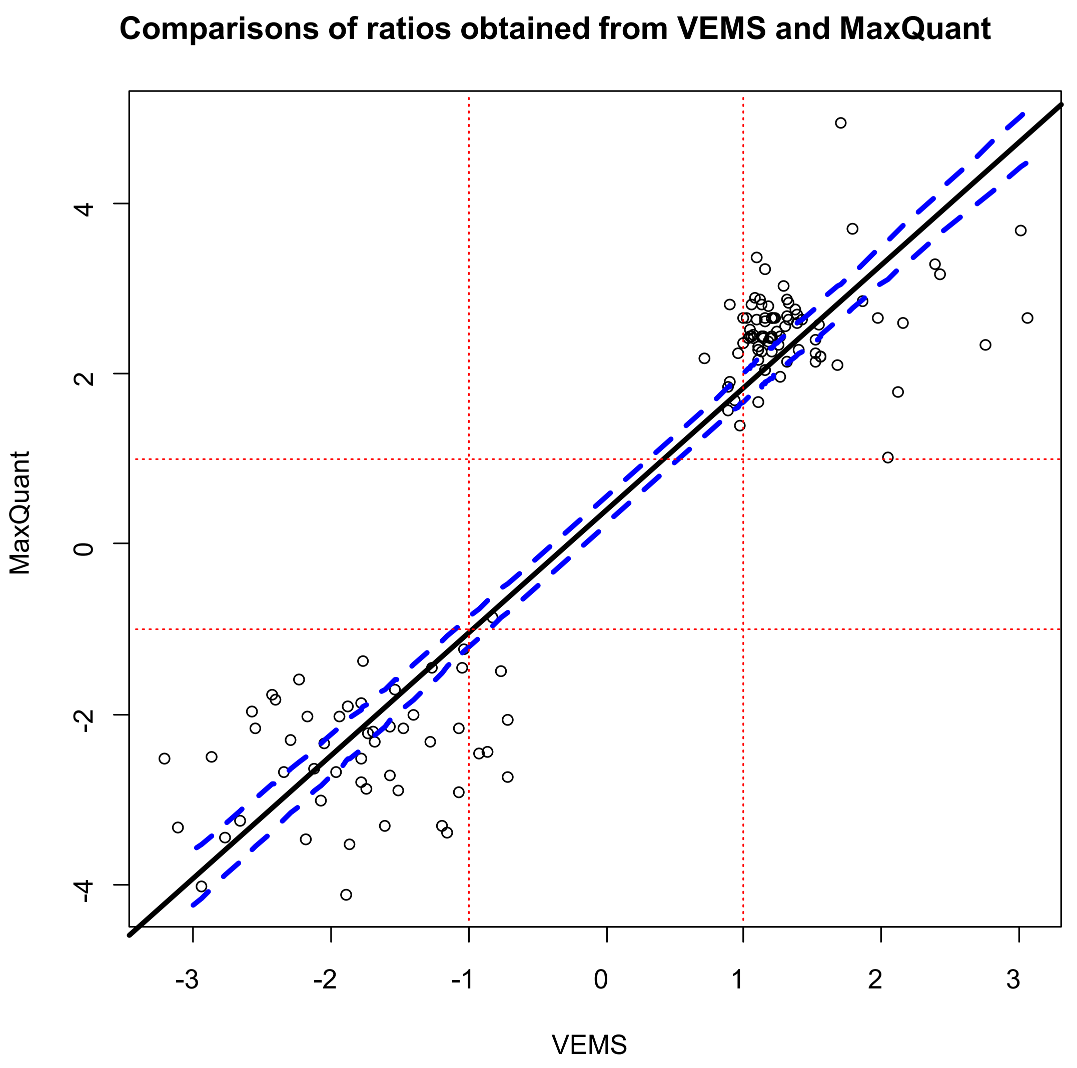
Comparisons of ratios obtained from VEMS and MaxQuant
MaxQuant
VEMS
4
2
0
-2
-4
-3
-2
-1
0
1
2
3

Figure S4

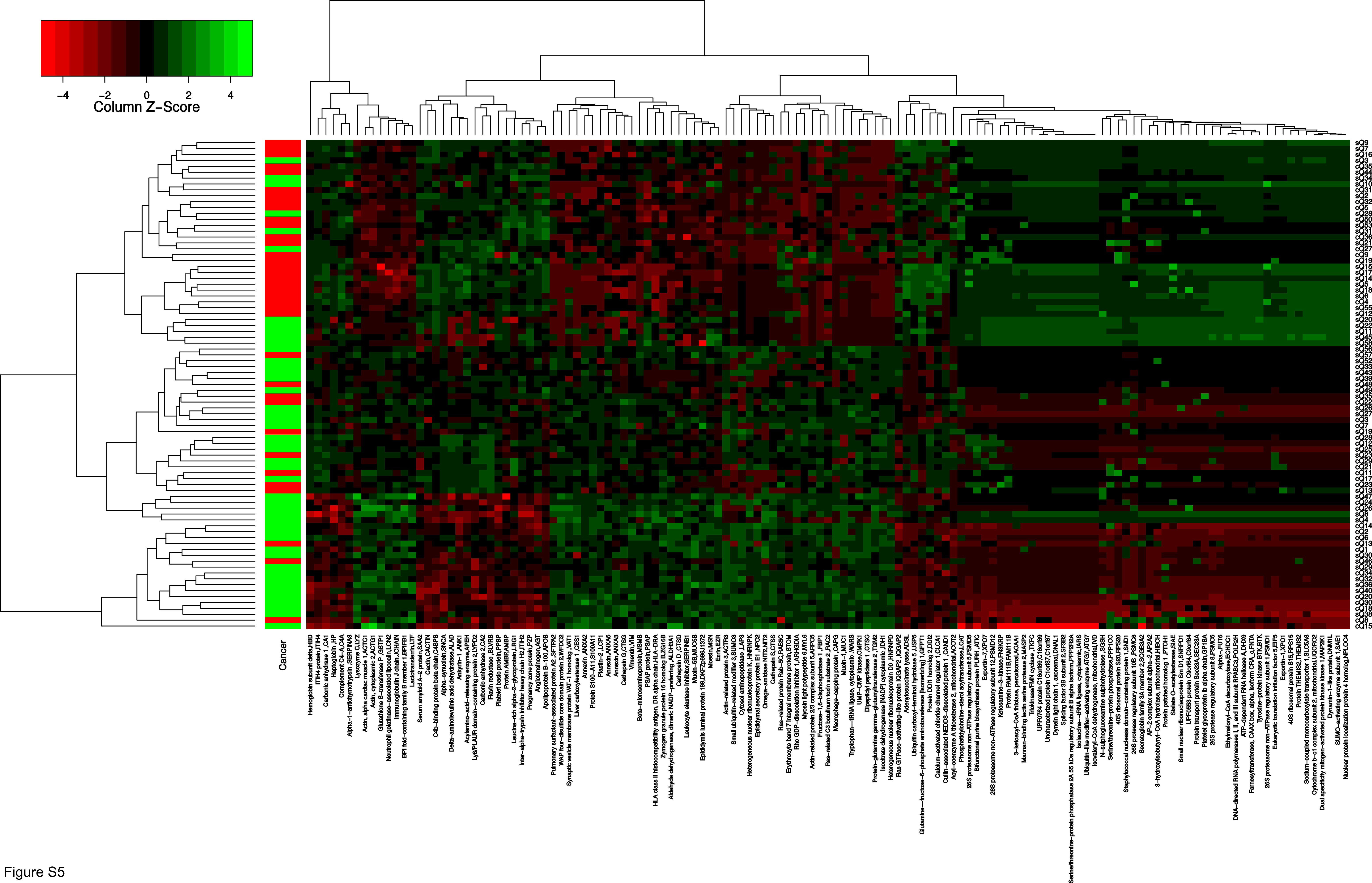

Figure S5

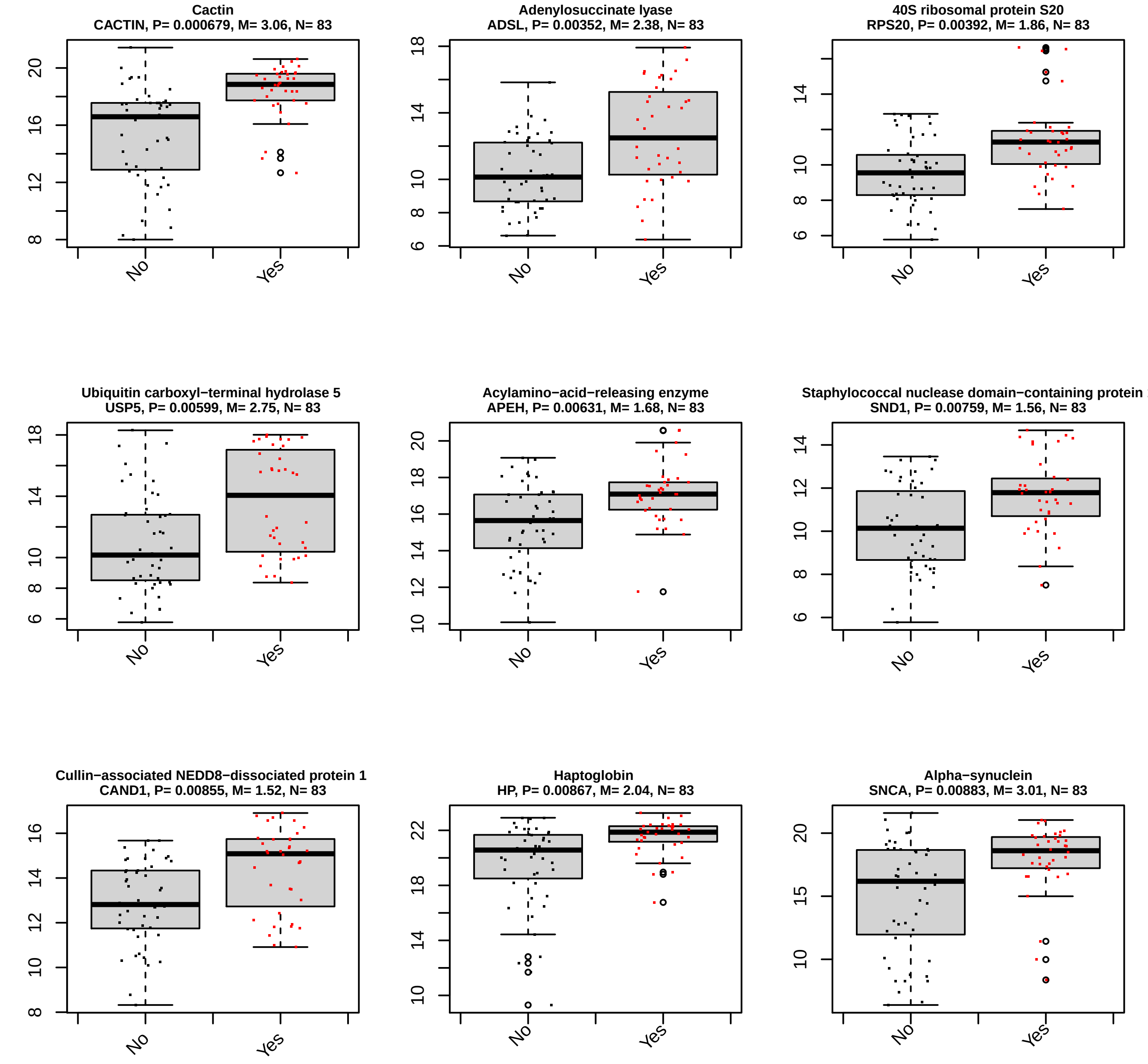

Cactin
CACTIN, P= 0.000679, M= 3.06, N= 83
Adenylosuccinate lyase
ADSL, P= 0.00352, M= 2.38, N= 83
40S ribosomal protein S20
RPS20, P= 0.00392, M= 1.86, N= 83
Ubiquitin carboxyl−terminal hydrolase 5
USP5, P= 0.00599, M= 2.75, N= 83
Acylamino−acid−releasing enzyme
APEH, P= 0.00631, M= 1.68, N= 83
Staphylococcal nuclease domain−containing protein
SND1, P= 0.00759, M= 1.56, N= 83
Cullin−associated NEDD8−dissociated protein 1
CAND1, P= 0.00855, M= 1.52, N= 83
Haptoglobin
HP, P= 0.00867, M= 2.04, N= 83
Alpha−synuclein
SNCA, P= 0.00883, M= 3.01, N= 83
No
Yes

Figure S6

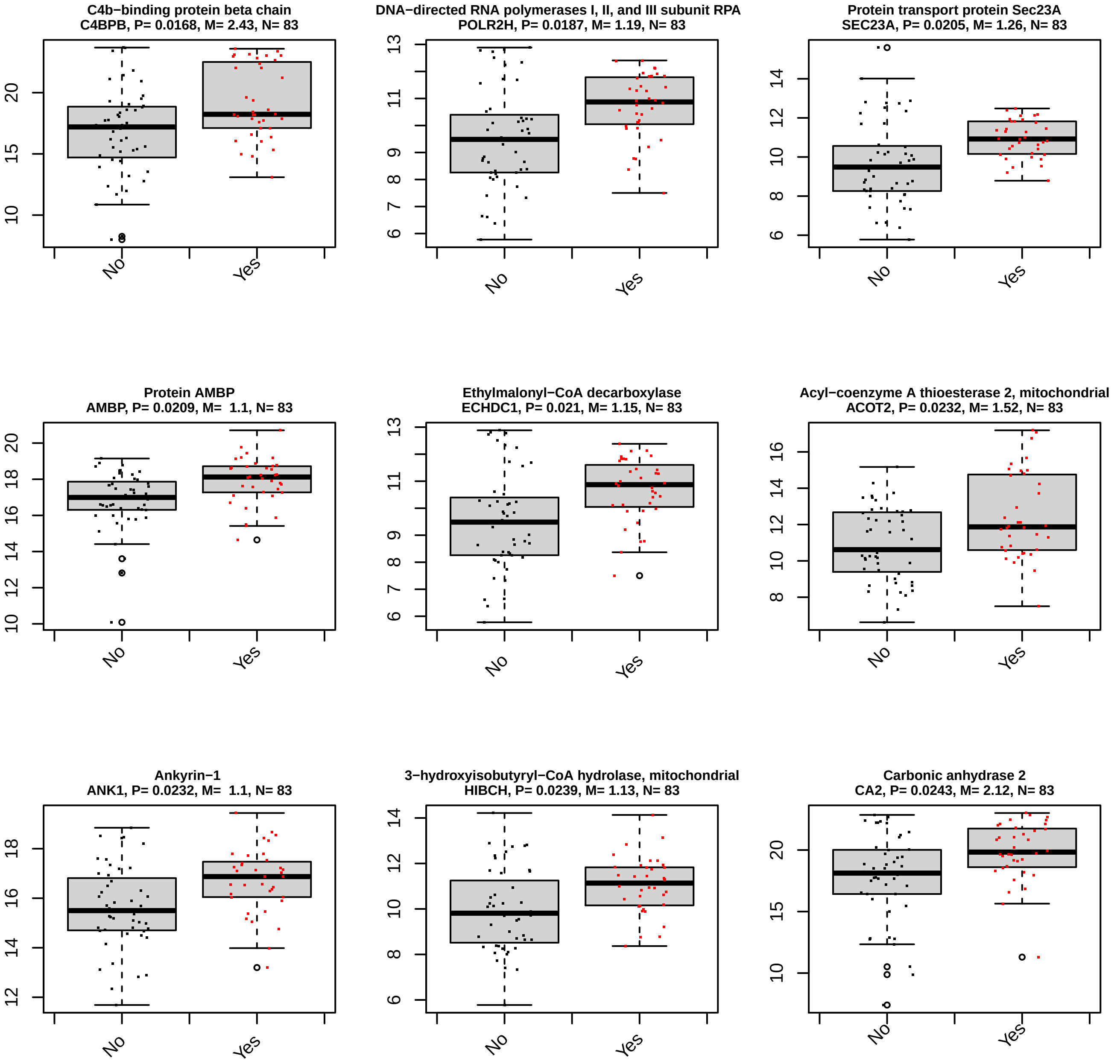
C4b−binding protein beta chain
C4BPB, P= 0.0168, M= 2.43, N= 83
10 15 20
No Yes
DNA−directed RNA polymerases I, II, and III subunit RPA
POLR2H, P= 0.0187, M= 1.19, N= 83
6 7 8 9 11 13
No Yes
Protein transport protein Sec23A
SEC23A, P= 0.0205, M= 1.26, N= 83
6 8 10 12 14
No Yes
Protein AMBP
AMBP, P= 0.0209, M= 1.1, N= 83
10 12 14 16 18 20
No Yes
Ethylmalonyl−CoA decarboxylase
ECHDC1, P= 0.021, M= 1.15, N= 83
6 7 8 9 11 13
No Yes
Acyl−coenzyme A thioesterase 2, mitochondrial
ACOT2, P= 0.0232, M= 1.52, N= 83
8 10 12 14 16
No Yes
Ankyrin−1
ANK1, P= 0.0232, M= 1.1, N= 83
12 14 16 18
No Yes
3−hydroxyisobutyryl−CoA hydrolase, mitochondrial
HIBCH, P= 0.0239, M= 1.13, N= 83
6 8 10 12 14
No Yes
Carbonic anhydrase 2
CA2, P= 0.0243, M= 2.12, N= 83
10 15 20
No Yes

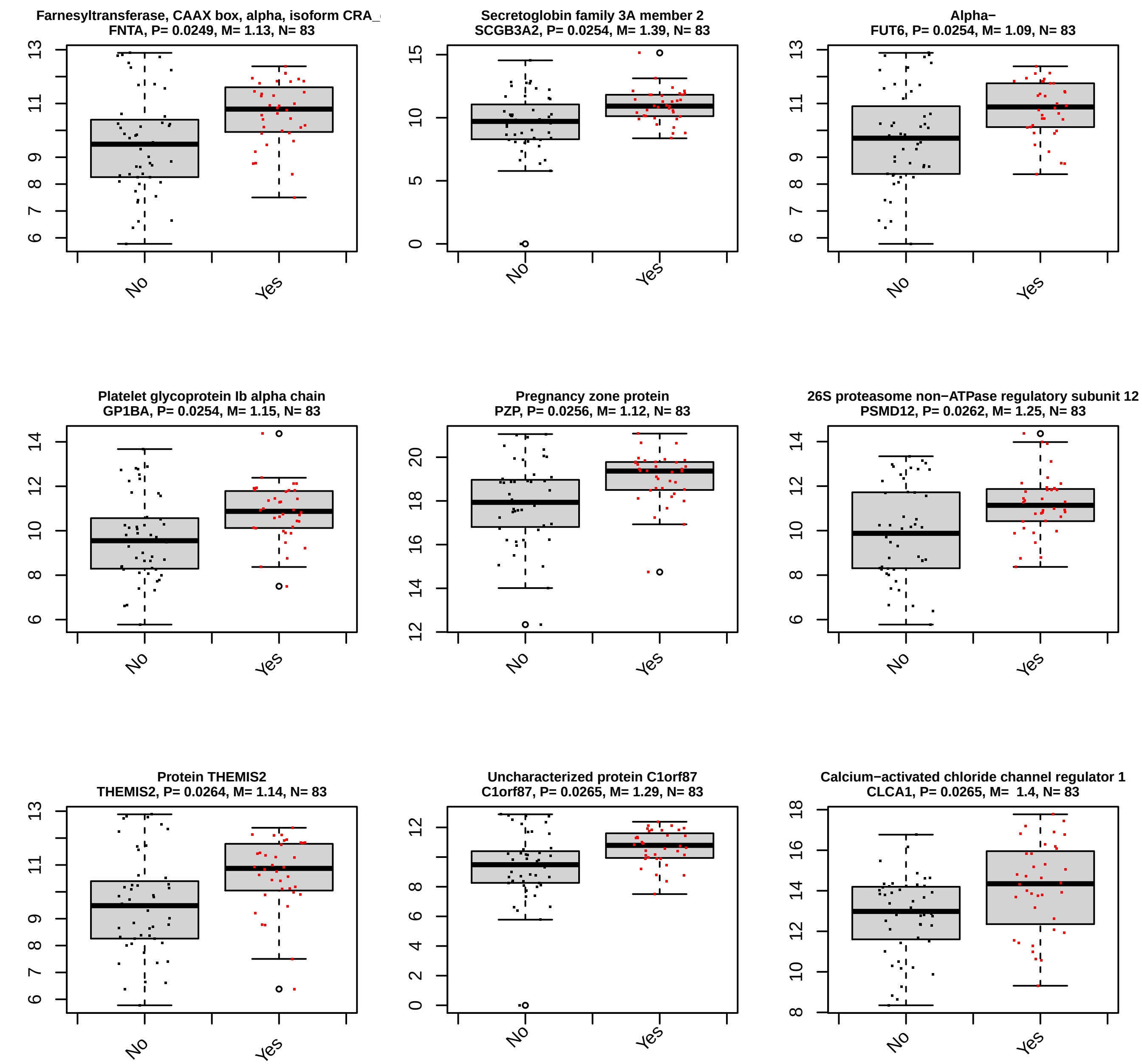
Farnesyltransferase, CAAX box, alpha, isoform CRA_
FNTA, P= 0.0249, M= 1.13, N= 83
6 7 8 9 11 13
No
Yes
Secretoglobin family 3A member 2
SCGB3A2, P= 0.0254, M= 1.39, N= 83
0 5 10 15
No
Yes
Alpha−
FUT6, P= 0.0254, M= 1.09, N= 83
6 7 8 9 11 13
No
Yes
Platelet glycoprotein Ib alpha chain
GP1BA, P= 0.0254, M= 1.15, N= 83
6 8 10 12 14
No
Yes
Pregnancy zone protein
PZP, P= 0.0256, M= 1.12, N= 83
12 14 16 18 20
No
Yes
26S proteasome non−ATPase regulatory subunit 12
PSMD12, P= 0.0262, M= 1.25, N= 83
6 8 10 12 14
No
Yes
Protein THEMIS2
THEMIS2, P= 0.0264, M= 1.14, N= 83
6 7 8 9 11 13
No
Yes
Uncharacterized protein C1orf87
C1orf87, P= 0.0265, M= 1.29, N= 83
0 2 4 6 8 12
No
Yes
Calcium−activated chloride channel regulator 1
CLCA1, P= 0.0265, M= 1.4, N= 83
8 10 12 14 16 18
No
Yes

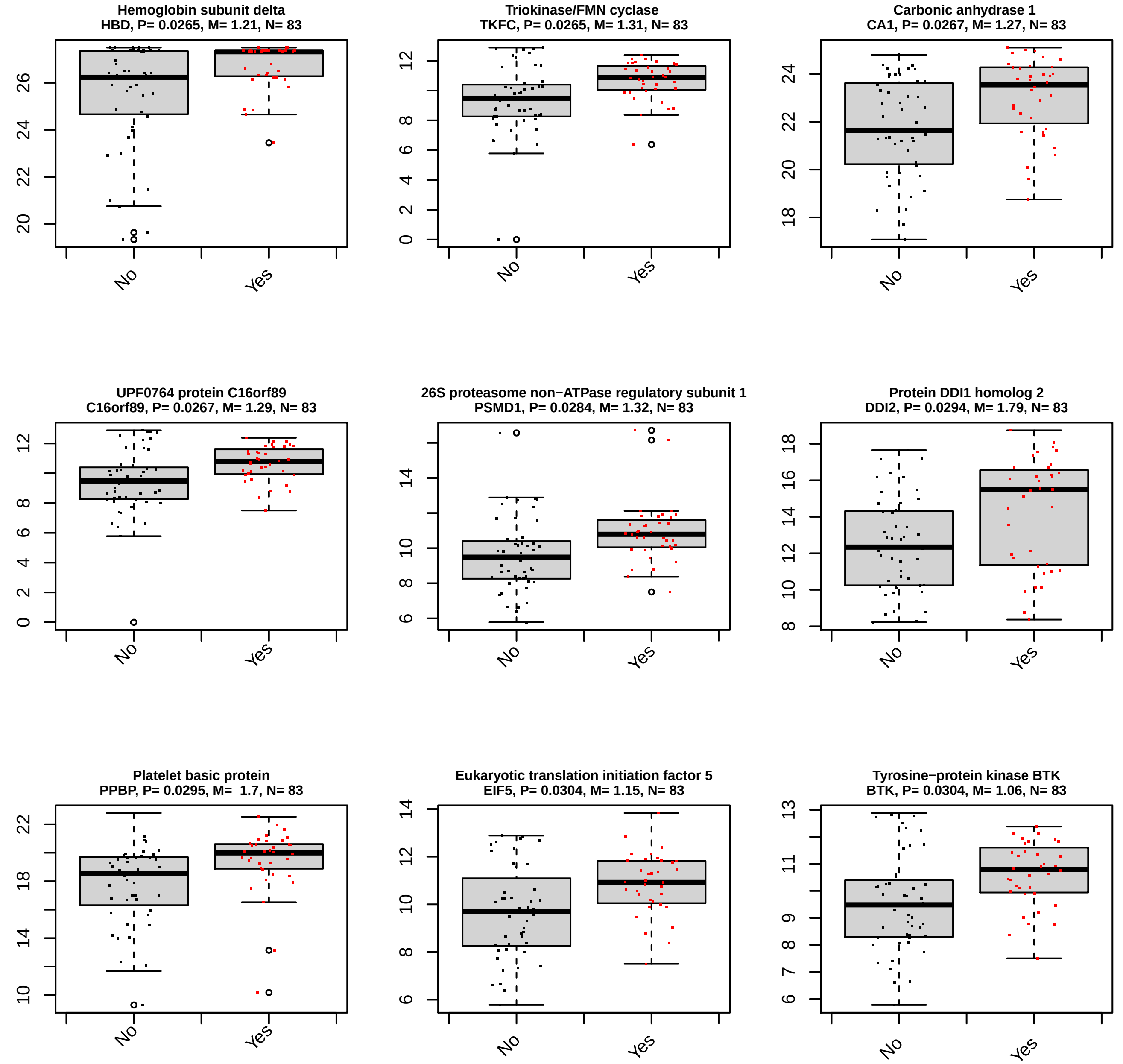
Hemoglobin subunit delta
HBD, P= 0.0265, M= 1.21, N= 83
Triokinase/FMN cyclase
TKFC, P= 0.0265, M= 1.31, N= 83
Carbonic anhydrase 1
CA1, P= 0.0267, M= 1.27, N= 83
UPF0764 protein C16orf89
C16orf89, P= 0.0267, M= 1.29, N= 83
26S proteasome non−ATPase regulatory subunit 1
PSMD1, P= 0.0284, M= 1.32, N= 83
Protein DDI1 homolog 2
DDI2, P= 0.0294, M= 1.79, N= 83
Platelet basic protein
PPBP, P= 0.0295, M= 1.7, N= 83
Eukaryotic translation initiation factor 5
EIF5, P= 0.0304, M= 1.15, N= 83
Tyrosine−protein kinase BTK
BTK, P= 0.0304, M= 1.06, N= 83
No
Yes

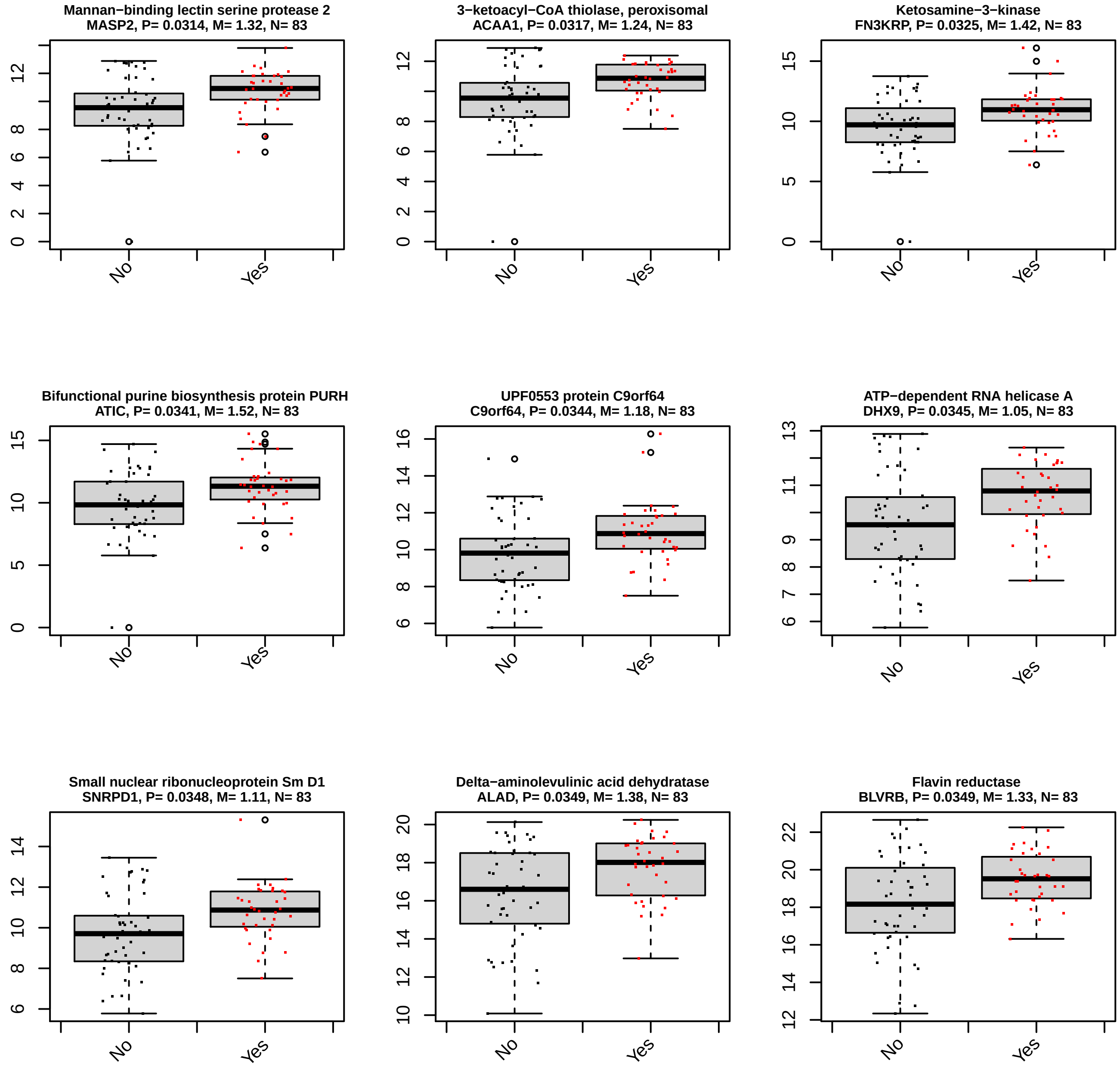
Mannan−binding lectin serine protease 2
MASP2, P= 0.0314, M= 1.32, N= 83
3−ketoacyl−CoA thiolase, peroxisomal
ACAA1, P= 0.0317, M= 1.24, N= 83
Ketosamine−3−kinase
FN3KRP, P= 0.0325, M= 1.42, N= 83
Bifunctional purine biosynthesis protein PURH
ATIC, P= 0.0341, M= 1.52, N= 83
UPF0553 protein C9orf64
C9orf64, P= 0.0344, M= 1.18, N= 83
ATP−dependent RNA helicase A
DHX9, P= 0.0345, M= 1.05, N= 83
Small nuclear ribonucleoprotein Sm D1
SNRPD1, P= 0.0348, M= 1.11, N= 83
Delta−aminolevulinic acid dehydratase
ALAD, P= 0.0349, M= 1.38, N= 83
Flavin reductase
BLVRB, P= 0.0349, M= 1.33, N= 83
No
Yes

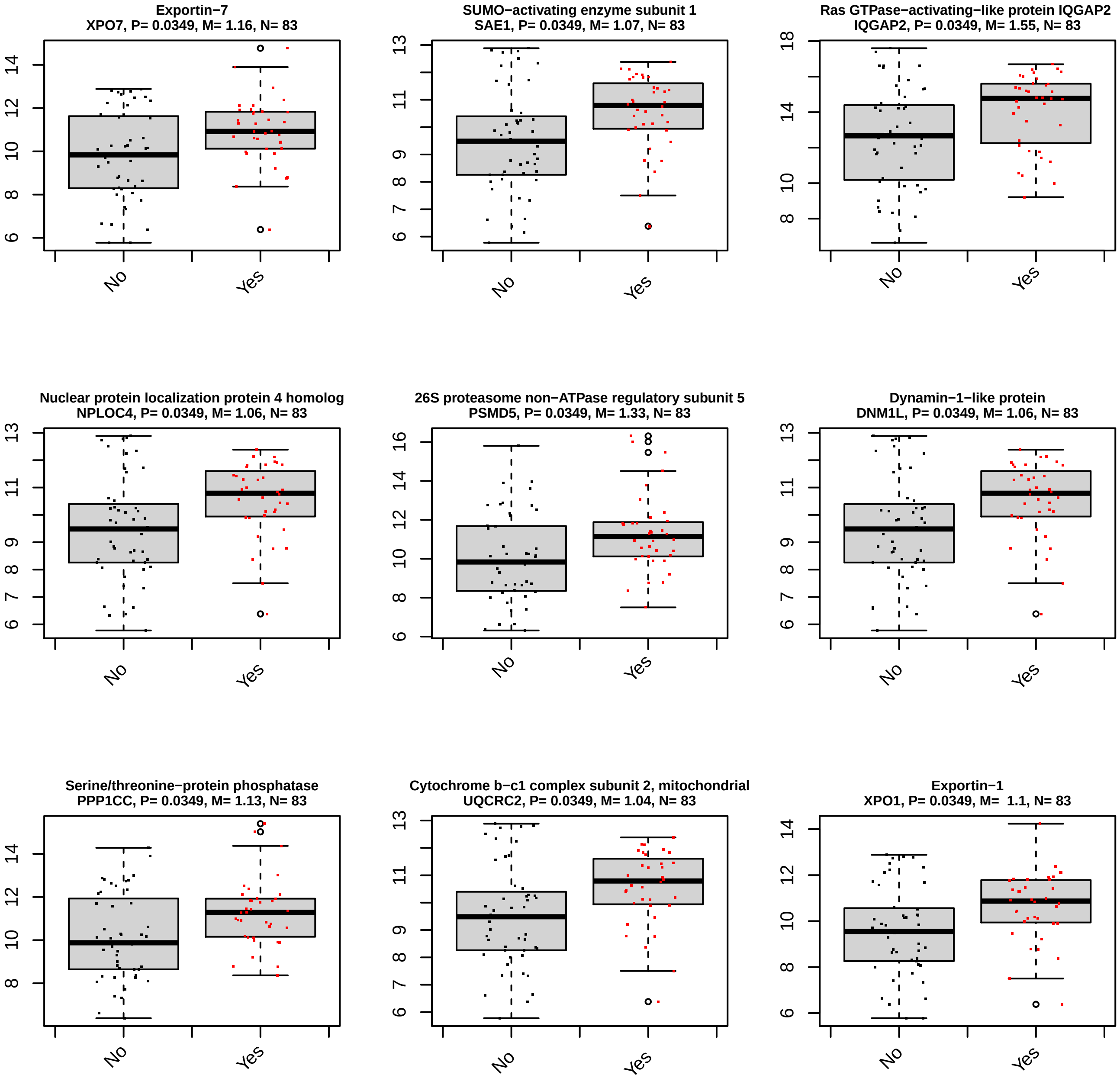

Exportin−7
XPO7, P= 0.0349, M= 1.16, N= 83
SUMO−activating enzyme subunit 1
SAE1, P= 0.0349, M= 1.07, N= 83
Ras GTPase−activating−like protein IQGAP2
IQGAP2, P= 0.0349, M= 1.55, N= 83
Nuclear protein localization protein 4 homolog
NPLOC4, P= 0.0349, M= 1.06, N= 83
26S proteasome non−ATPase regulatory subunit 5
PSMD5, P= 0.0349, M= 1.33, N= 83
Dynamin−1−like protein
DNM1L, P= 0.0349, M= 1.06, N= 83
Serine/threonine−protein phosphatase
PPP1CC, P= 0.0349, M= 1.13, N= 83
Cytochrome b−c1 complex subunit 2, mitochondrial
UQCRC2, P= 0.0349, M= 1.04, N= 83
Exportin−1
XPO1, P= 0.0349, M= 1.1, N= 83
No
Yes

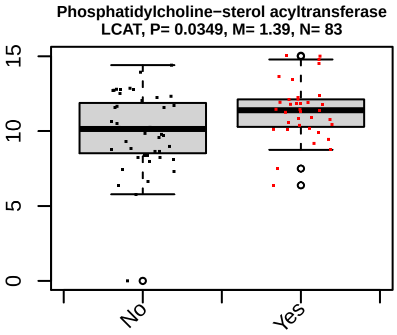
Phosphatidylcholine−sterol acyltransferase
LCAT, P= 0.0349, M= 1.39, N= 83
0
5
10
15
No
Yes

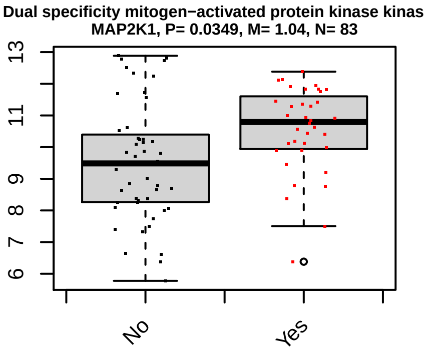
Dual specificity mitogen−activated protein kinase kinas
MAP2K1, P= 0.0349, M= 1.04, N= 83
6
7
8
9
11
13
No
Yes

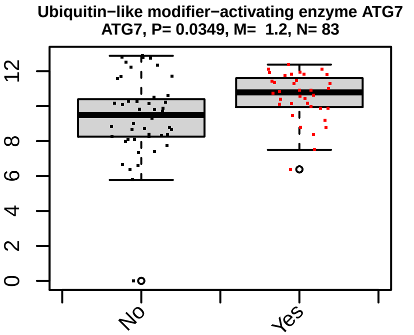
Ubiquitin−like modifier−activating enzyme ATG7
ATG7, P= 0.0349, M= 1.2, N= 83
0
2
4
6
8
12
No
Yes

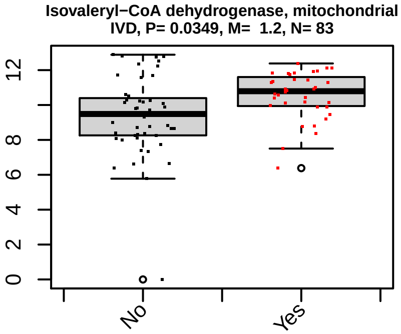
Isovaleryl−CoA dehydrogenase, mitochondrial
IVD, P= 0.0349, M= 1.2, N= 83
0
2
4
6
8
12
No
Yes

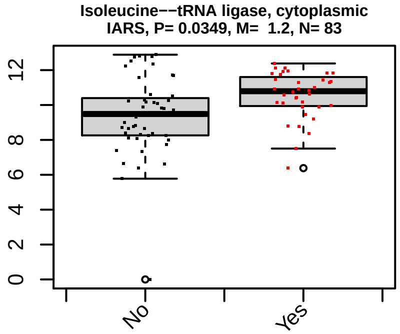
Isoleucine−−tRNA ligase, cytoplasmic
IARS, P= 0.0349, M= 1.2, N= 83
0
2
4
6
8
12
No
Yes

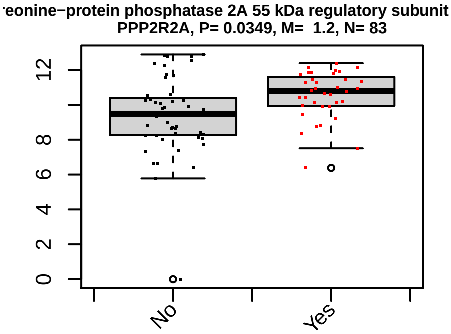
eonine−protein phosphatase 2A 55 kDa regulatory subunit
PPP2R2A, P= 0.0349, M= 1.2, N= 83
0
2
4
6
8
12
No
Yes

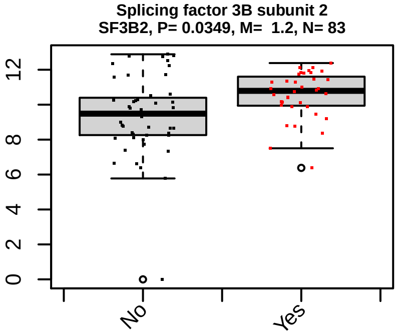
Splicing factor 3B subunit 2
SF3B2, P= 0.0349, M= 1.2, N= 83
0
2
4
6
8
12
No
Yes

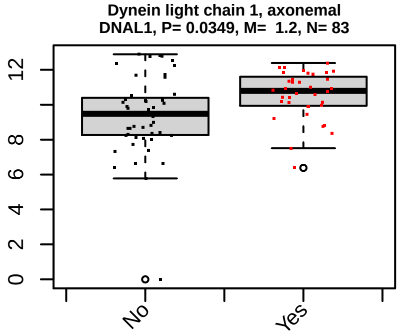
Dynein light chain 1, axonemal
DNAL1, P= 0.0349, M= 1.2, N= 83
0
2
4
6
8
12
No
Yes

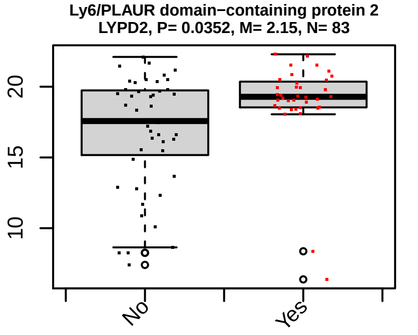
Ly6/PLAUR domain−containing protein 2
LYPD2, P= 0.0352, M= 2.15, N= 83
10
15
20
No
Yes

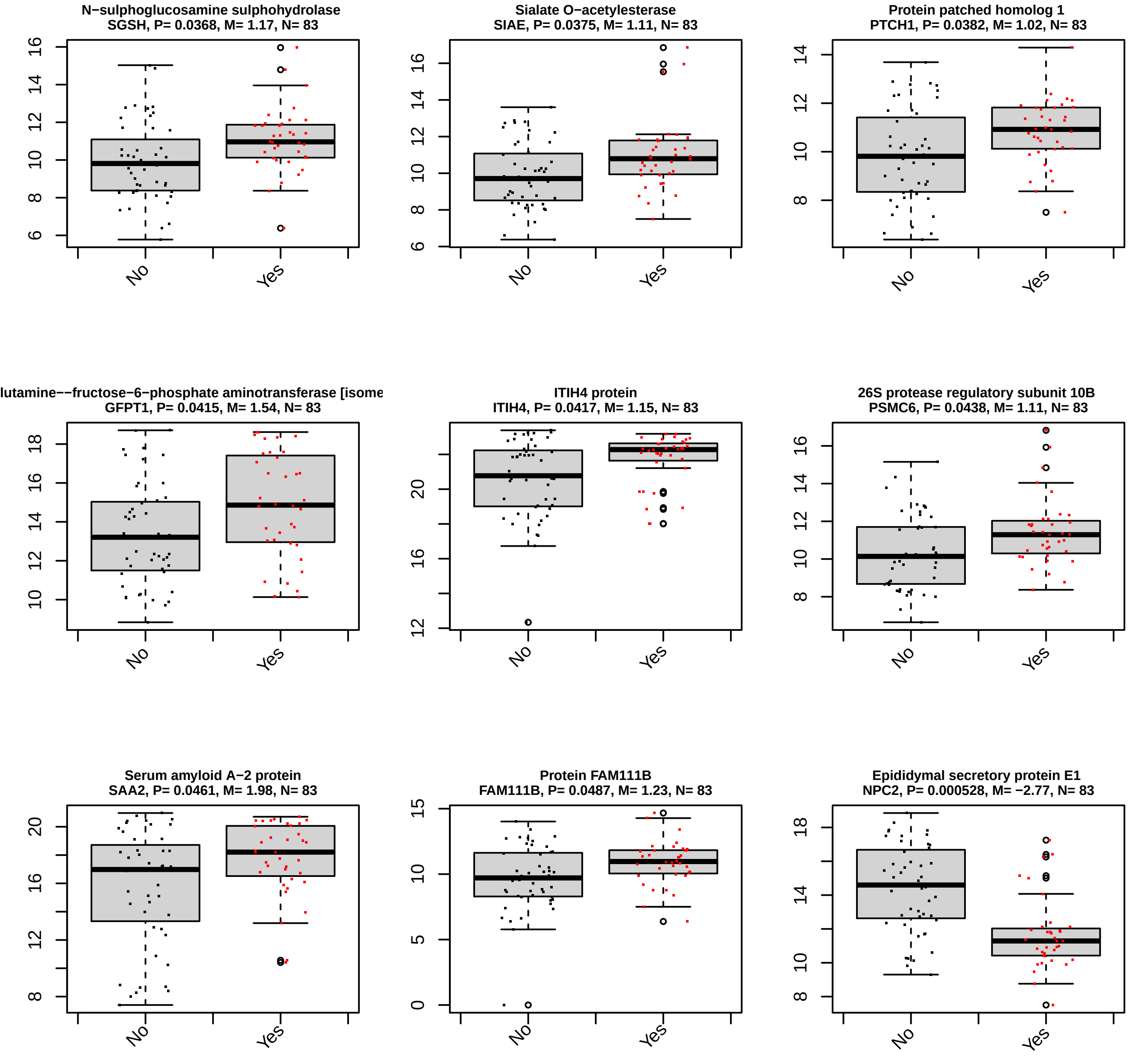
N−sulphoglucosamine sulphohydrolase
SGSH, P= 0.0368, M= 1.17, N= 83
Sialate O−acetylesterase
SIAE, P= 0.0375, M= 1.11, N= 83
Protein patched homolog 1
PTCH1, P= 0.0382, M= 1.02, N= 83
lutamine−−fructose−6−phosphate aminotransferase [isome
GFPT1, P= 0.0415, M= 1.54, N= 83
ITIH4 protein
ITIH4, P= 0.0417, M= 1.15, N= 83
26S protease regulatory subunit 10B
PSMC6, P= 0.0438, M= 1.11, N= 83
Serum amyloid A−2 protein
SAA2, P= 0.0461, M= 1.98, N= 83
Protein FAM111B
FAM111B, P= 0.0487, M= 1.23, N= 83
Epididymal secretory protein E1
NPC2, P= 0.000528, M= −2.77, N= 83
No
Yes

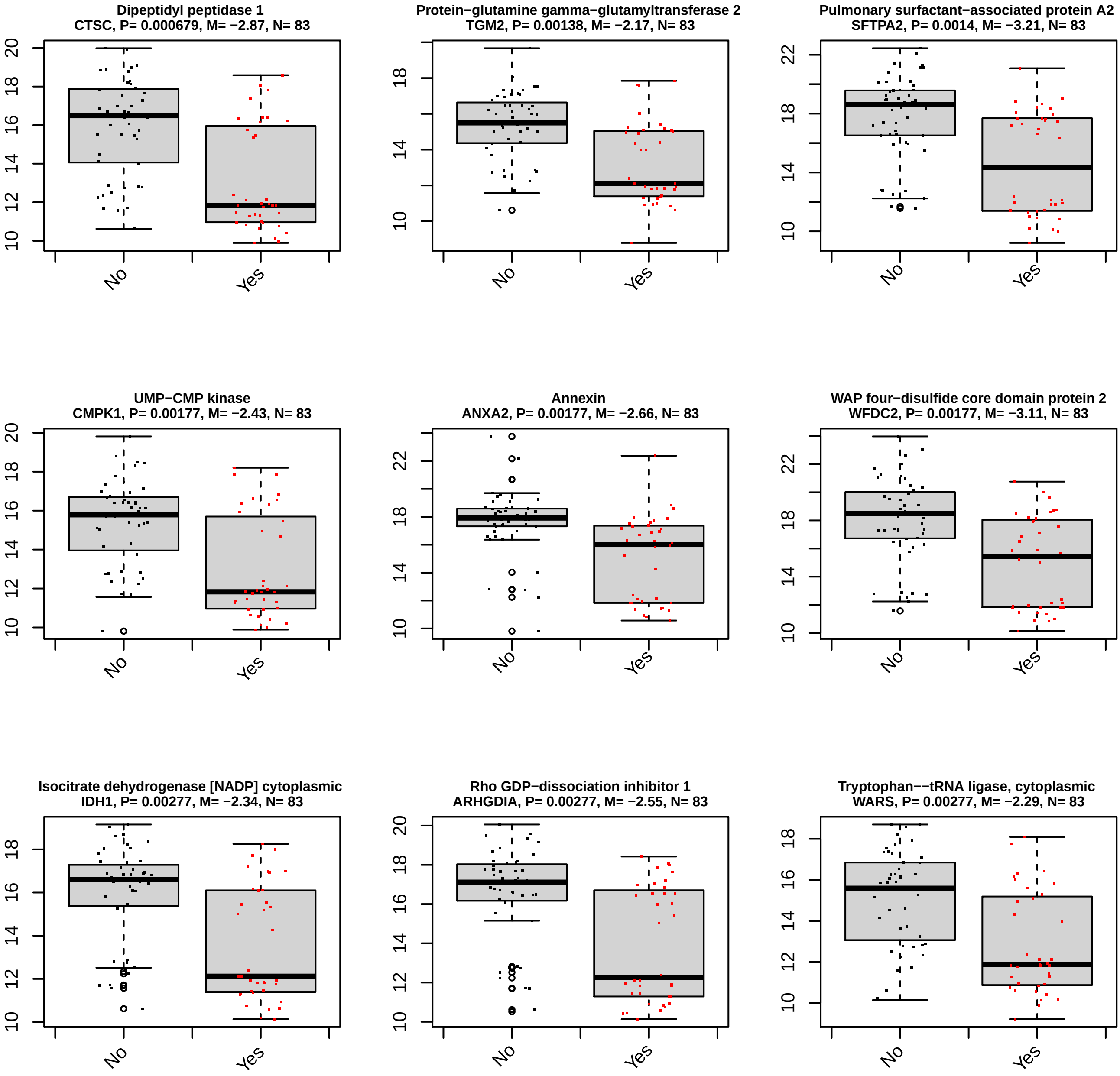

Dipeptidyl peptidase 1
CTSC, P= 0.000679, M= −2.87, N= 83
Protein−glutamine gamma−glutamyltransferase 2
TGM2, P= 0.00138, M= −2.17, N= 83
Pulmonary surfactant−associated protein A2
SFTPA2, P= 0.0014, M= −3.21, N= 83
UMP−CMP kinase
CMPK1, P= 0.00177, M= −2.43, N= 83
Annexin
ANXA2, P= 0.00177, M= −2.66, N= 83
WAP four−disulfide core domain protein 2
WFDC2, P= 0.00177, M= −3.11, N= 83
Isocitrate dehydrogenase [NADP] cytoplasmic
IDH1, P= 0.00277, M= −2.34, N= 83
Rho GDP−dissociation inhibitor 1
ARHGDIA, P= 0.00277, M= −2.55, N= 83
Tryptophan−−tRNA ligase, cytoplasmic
WARS, P= 0.00277, M= −2.29, N= 83
No
Yes

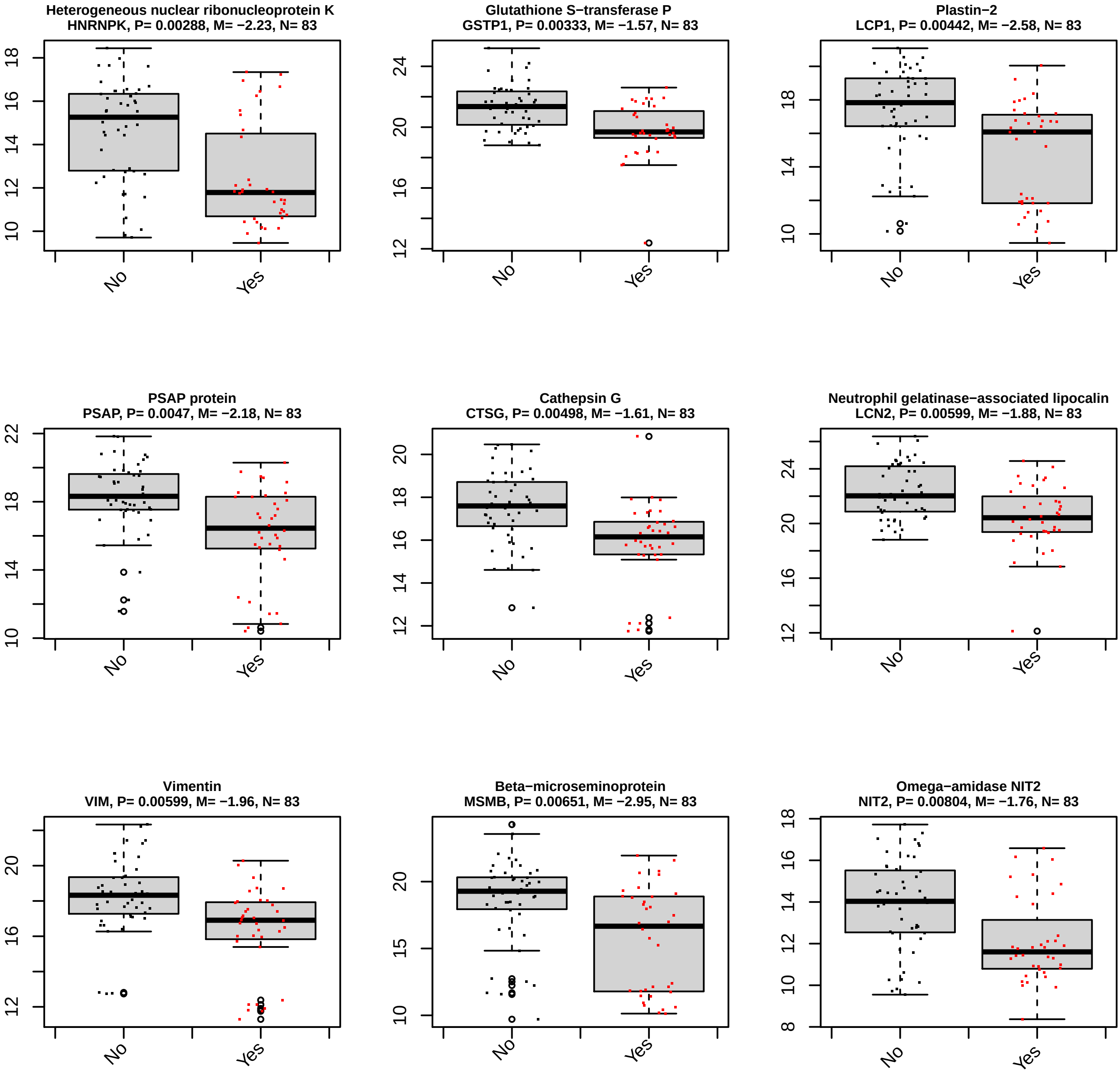
Heterogeneous nuclear ribonucleoprotein K
HNRNPK, P= 0.00288, M= −2.23, N= 83
Glutathione S−transferase P
GSTP1, P= 0.00333, M= −1.57, N= 83
Plastin−2
LCP1, P= 0.00442, M= −2.58, N= 83
PSAP protein
PSAP, P= 0.0047, M= −2.18, N= 83
Cathepsin G
CTSG, P= 0.00498, M= −1.61, N= 83
Neutrophil gelatinase−associated lipocalin
LCN2, P= 0.00599, M= −1.88, N= 83
Vimentin
VIM, P= 0.00599, M= −1.96, N= 83
Beta−microseminoprotein
MSMB, P= 0.00651, M= −2.95, N= 83
Omega−amidase NIT2
NIT2, P= 0.00804, M= −1.76, N= 83
No
Yes

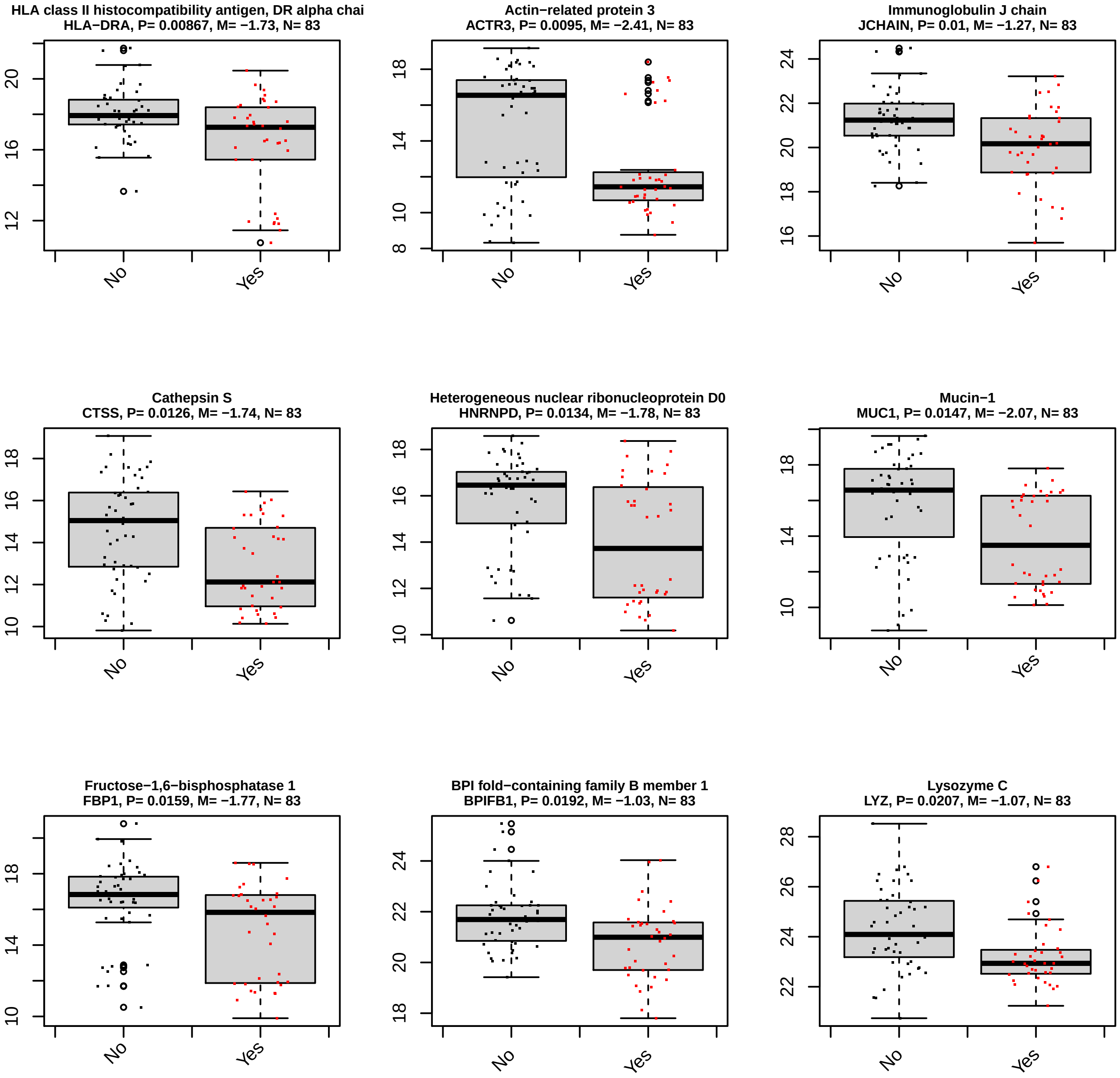

HLA class II histocompatibility antigen, DR alpha chai
HLA−DRA, P= 0.00867, M= −1.73, N= 83
12
16
20
No
Yes
Actin−related protein 3
ACTR3, P= 0.0095, M= −2.41, N= 83
8
10
14
18
No
Yes
Immunoglobulin J chain
JCHAIN, P= 0.01, M= −1.27, N= 83
16
18
20
22
24
No
Yes
Cathepsin S
CTSS, P= 0.0126, M= −1.74, N= 83
10
12
14
16
18
No
Yes
Heterogeneous nuclear ribonucleoprotein D0
HNRNPD, P= 0.0134, M= −1.78, N= 83
10
12
14
16
18
No
Yes
Mucin−1
MUC1, P= 0.0147, M= −2.07, N= 83
10
14
18
No
Yes
Fructose−1,6−bisphosphatase 1
FBP1, P= 0.0159, M= −1.77, N= 83
10
14
18
No
Yes
BPI fold−containing family B member 1
BPIFB1, P= 0.0192, M= −1.03, N= 83
18
20
22
24
No
Yes
Lysozyme C
LYZ, P= 0.0207, M= −1.07, N= 83
22
24
26
28
No
Yes

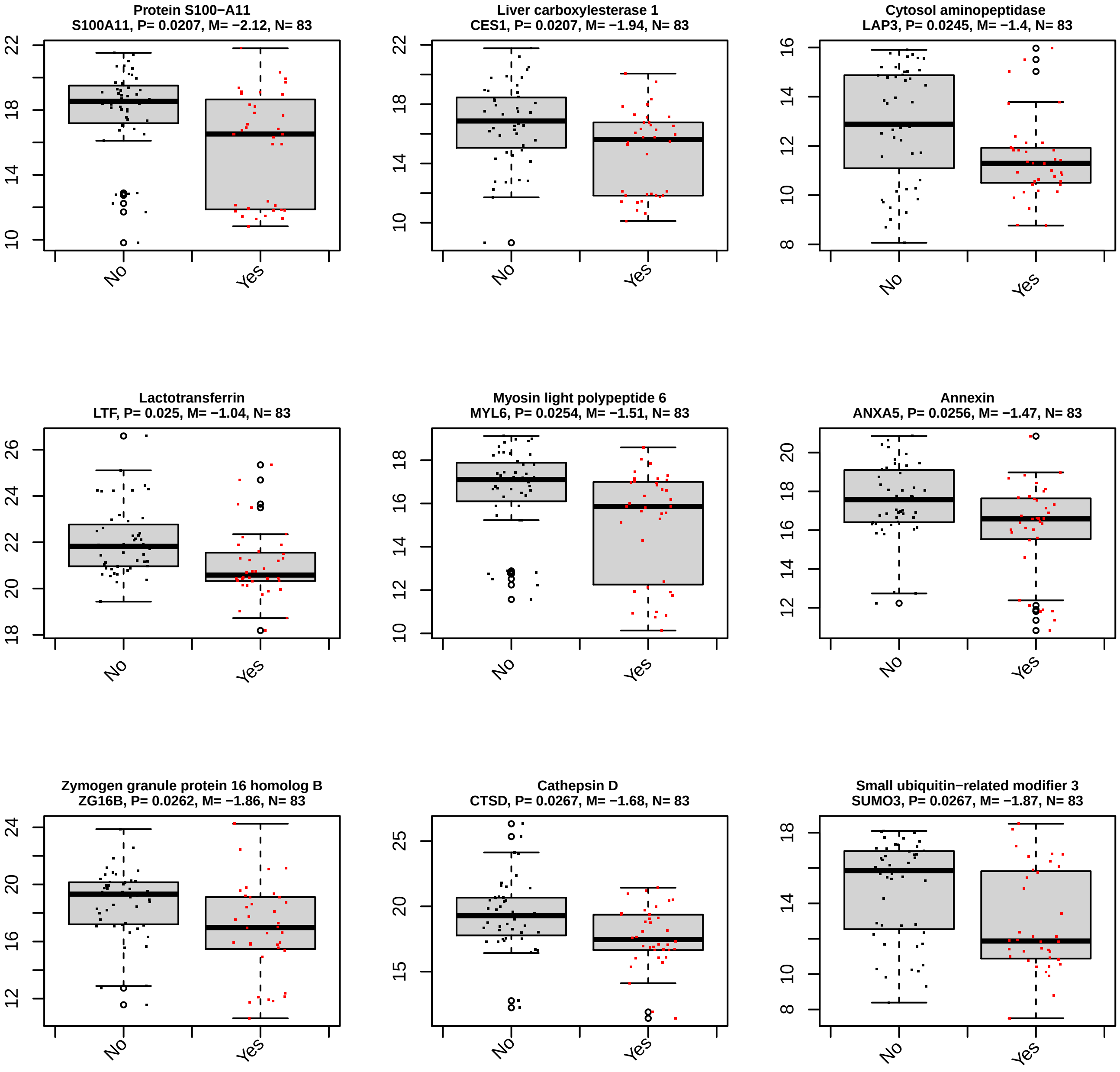

Protein S100−A11
S100A11, P= 0.0207, M= −2.12, N= 83
Liver carboxylesterase 1
CES1, P= 0.0207, M= −1.94, N= 83
Cytosol aminopeptidase
LAP3, P= 0.0245, M= −1.4, N= 83
Lactotransferrin
LTF, P= 0.025, M= −1.04, N= 83
Myosin light polypeptide 6
MYL6, P= 0.0254, M= −1.51, N= 83
Annexin
ANXA5, P= 0.0256, M= −1.47, N= 83
Zymogen granule protein 16 homolog B
ZG16B, P= 0.0262, M= −1.86, N= 83
Cathepsin D
CTSD, P= 0.0267, M= −1.68, N= 83
Small ubiquitin−related modifier 3
SUMO3, P= 0.0267, M= −1.87, N= 83
No
Yes

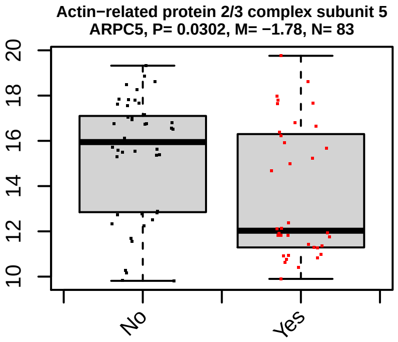
Actin−related protein 2/3 complex subunit 5
ARPC5, P= 0.0302, M= −1.78, N= 83
10
12
14
16
18
20
No
Yes

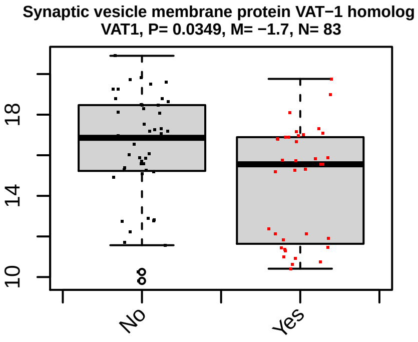
Synaptic vesicle membrane protein VAT−1 homolog
VAT1, P= 0.0349, M= −1.7, N= 83
10
14
18
No
Yes

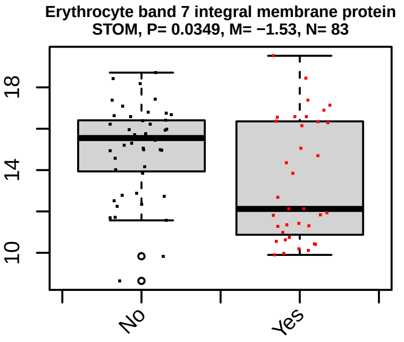
Erythrocyte band 7 integral membrane protein
STOM, P= 0.0349, M= −1.53, N= 83
10
14
18
No
Yes

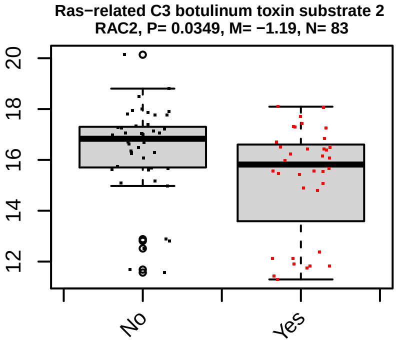
Ras−related C3 botulinum toxin substrate 2
RAC2, P= 0.0349, M= −1.19, N= 83
12
14
16
18
20
No
Yes

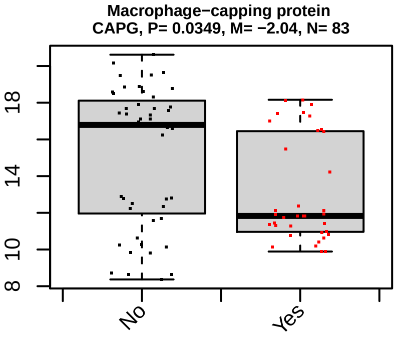
Macrophage−capping protein
CAPG, P= 0.0349, M= −2.04, N= 83
8
10
14
18
No
Yes

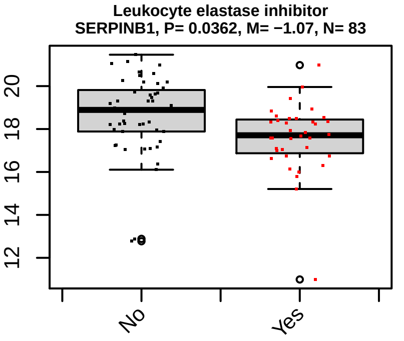
Leukocyte elastase inhibitor
SERPINB1, P= 0.0362, M= −1.07, N= 83
12
14
16
18
20
No
Yes

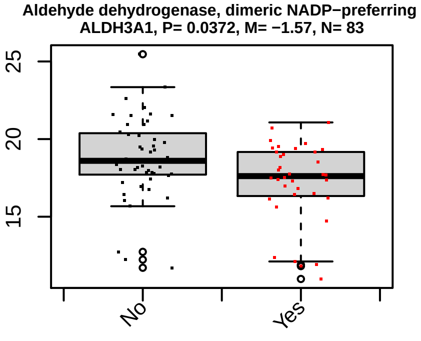
Aldehyde dehydrogenase, dimeric NADP−preferring
ALDH3A1, P= 0.0372, M= −1.57, N= 83
15
20
25
No
Yes

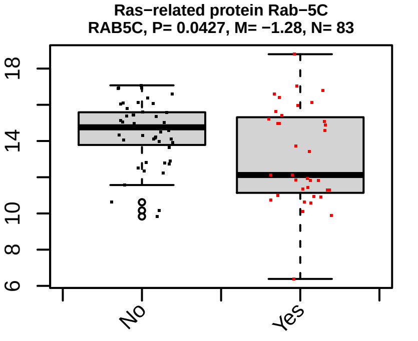
Ras−related protein Rab−5C
RAB5C, P= 0.0427, M= −1.28, N= 83
6
8
10
14
18
No
Yes

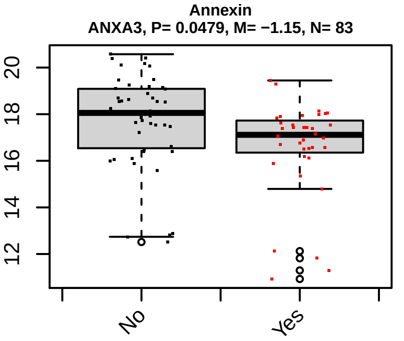
Annexin
ANXA3, P= 0.0479, M= −1.15, N= 83
12
14
16
18
20
No
Yes

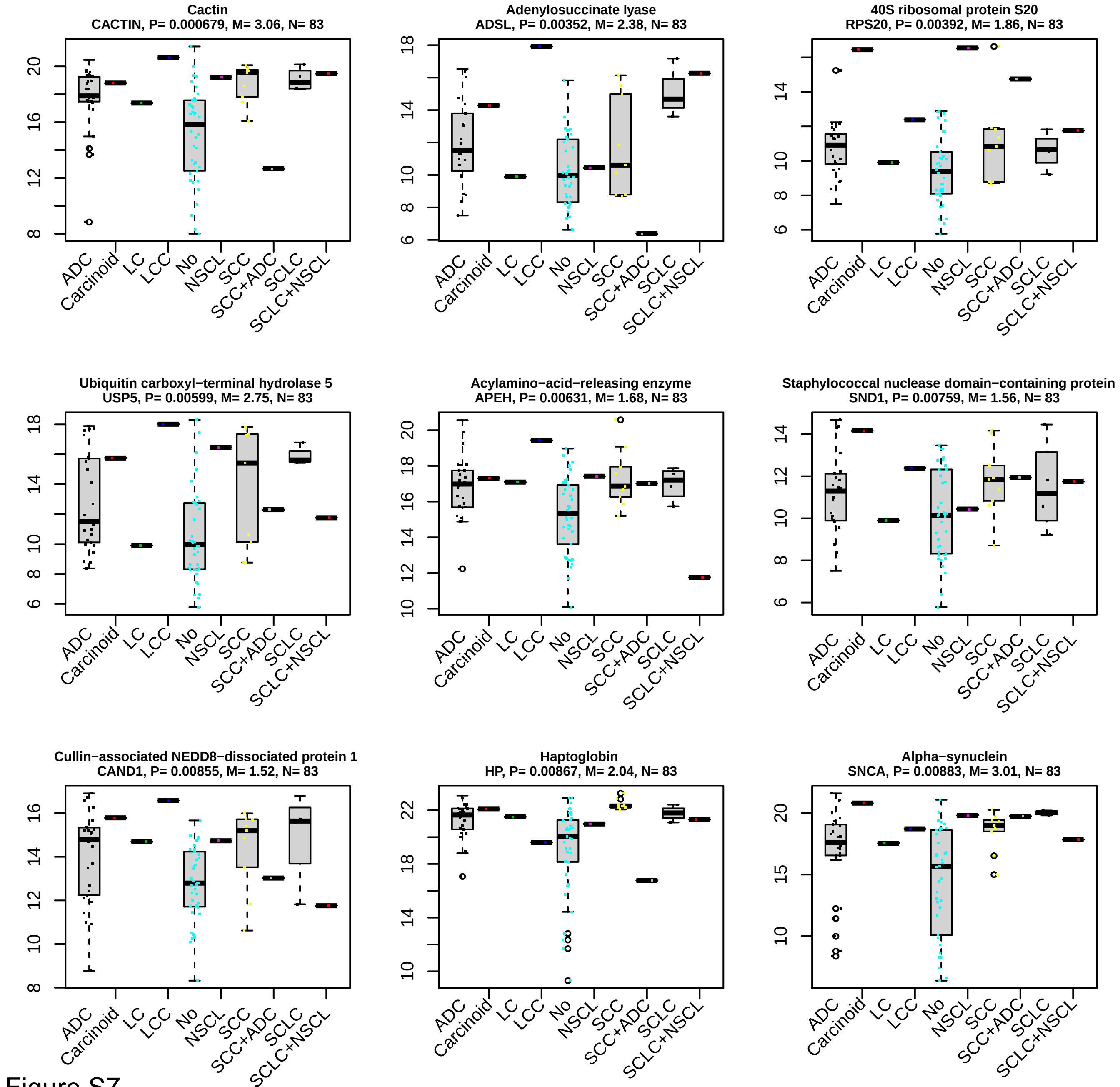
Cactin
CACTIN, P= 0.000679, M= 3.06, N= 83
Adenylosuccinate lyase
ADSL, P= 0.00352, M= 2.38, N= 83
40S ribosomal protein S20
RPS20, P= 0.00392, M= 1.86, N= 83
Ubiquitin carboxyl−terminal hydrolase 5
USP5, P= 0.00599, M= 2.75, N= 83
Acylamino−acid−releasing enzyme
APEH, P= 0.00631, M= 1.68, N= 83
Staphylococcal nuclease domain−containing protein
SND1, P= 0.00759, M= 1.56, N= 83
Cullin−associated NEDD8−dissociated protein 1
CAND1, P= 0.00855, M= 1.52, N= 83
Haptoglobin
HP, P= 0.00867, M= 2.04, N= 83
Alpha−synuclein
SNCA, P= 0.00883, M= 3.01, N= 83
ADC
Carcinoid
LC
LCC
No
NSCL
SCC
SCC+ADC
SCLC
SCLC+NSCL

Figure S7

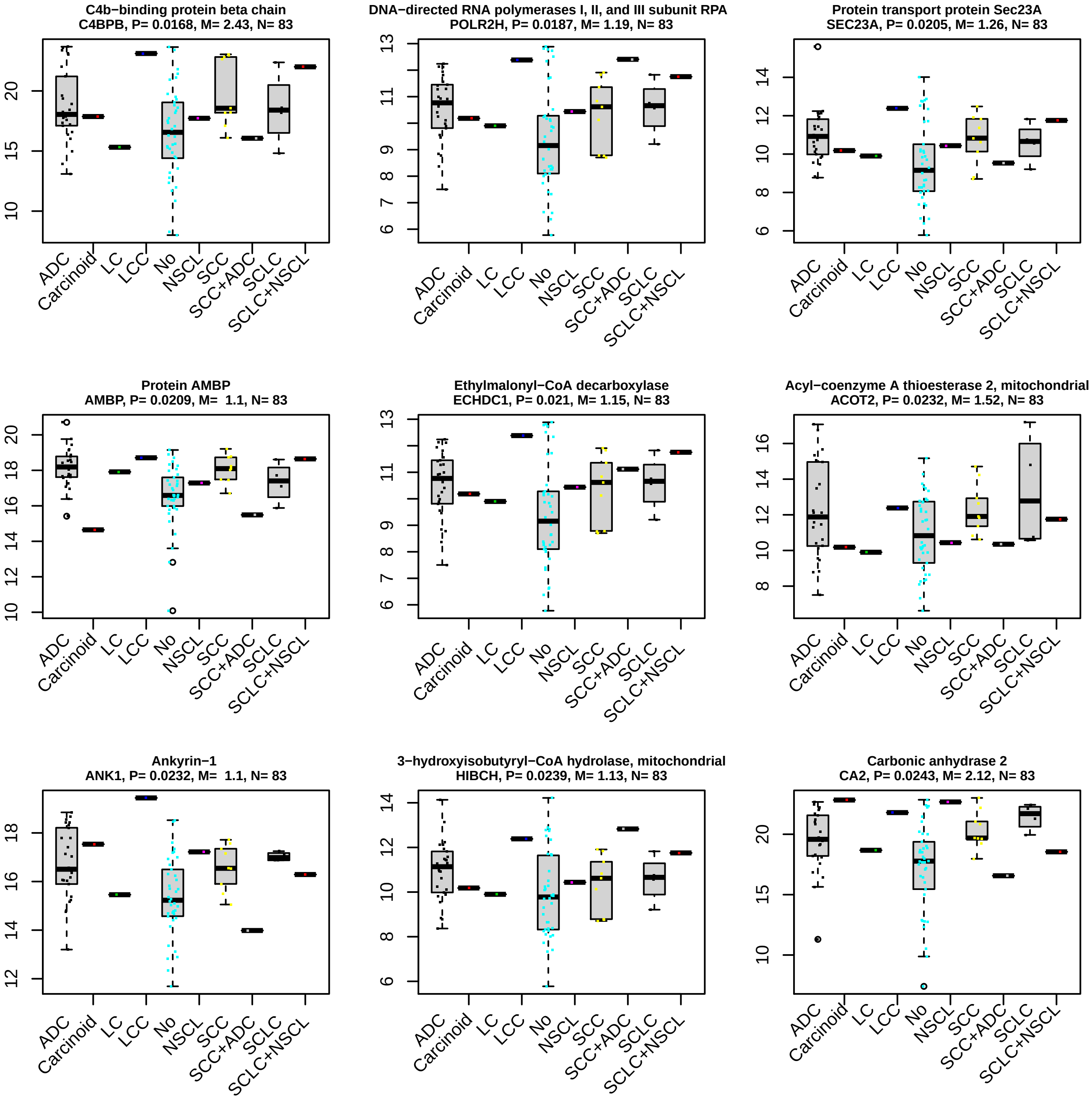
C4b−binding protein beta chain
C4BPB, P= 0.0168, M= 2.43, N= 83
10
15
20
DNA−directed RNA polymerases I, II, and III subunit RPA
POLR2H, P= 0.0187, M= 1.19, N= 83
6
7
8
9
11
13
Protein transport protein Sec23A
SEC23A, P= 0.0205, M= 1.26, N= 83
6
8
10
12
14
Protein AMBP
AMBP, P= 0.0209, M= 1.1, N= 83
10
12
14
16
18
20
Ethylmalonyl−CoA decarboxylase
ECHDC1, P= 0.021, M= 1.15, N= 83
6
7
8
9
11
13
Acyl−coenzyme A thioesterase 2, mitochondrial
ACOT2, P= 0.0232, M= 1.52, N= 83
8
10
12
14
16
Ankyrin−1
ANK1, P= 0.0232, M= 1.1, N= 83
12
14
16
18
3−hydroxyisobutyryl−CoA hydrolase, mitochondrial
HIBCH, P= 0.0239, M= 1.13, N= 83
6
8
10
12
14
Carbonic anhydrase 2
CA2, P= 0.0243, M= 2.12, N= 83
10
15
20
ADC
Carcinoid
LC
LCC
No
NSCL
SCC
SCC+ADC
SCLC
SCLC+NSCL

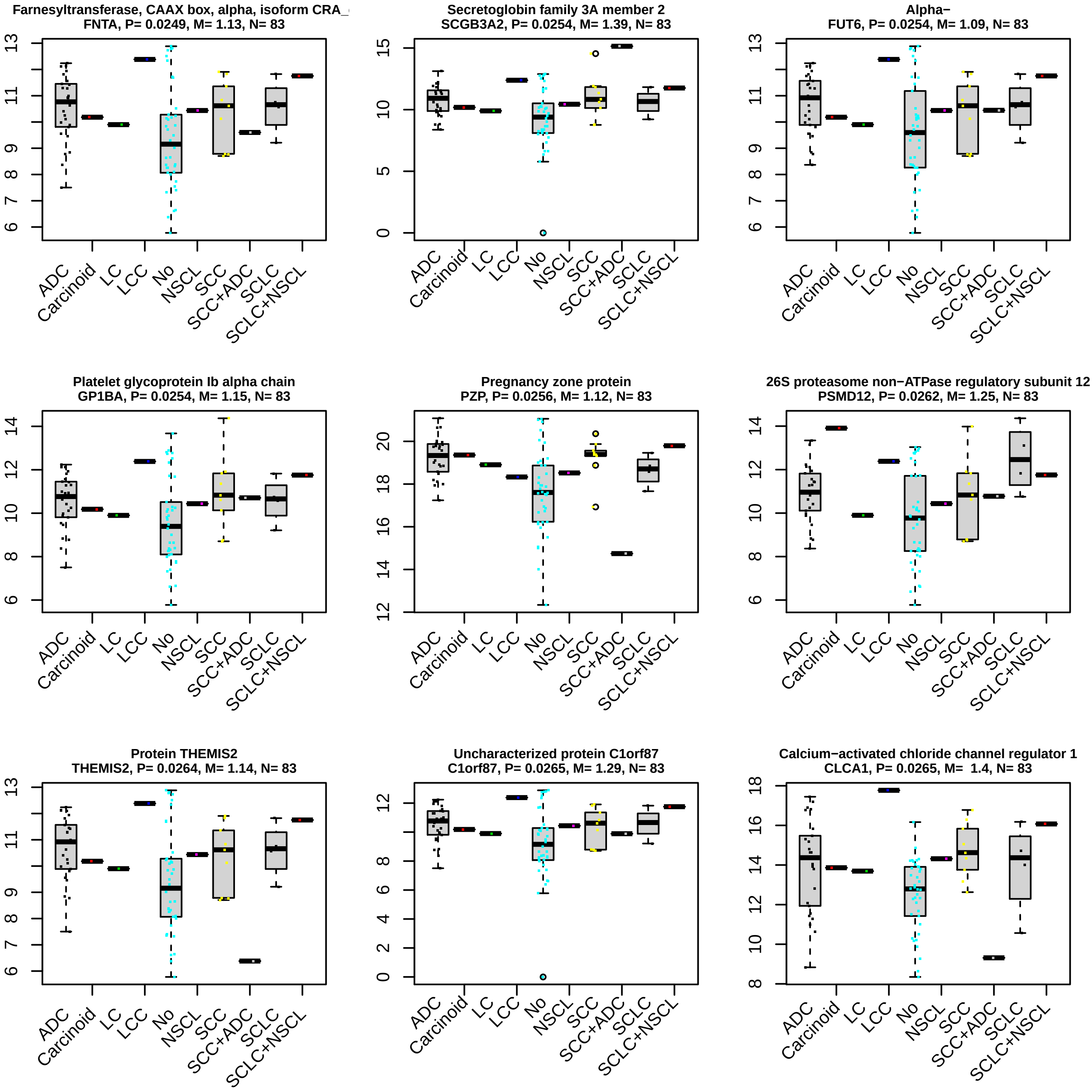

Farnesyltransferase, CAAX box, alpha, isoform CRA_
FNTA, P= 0.0249, M= 1.13, N= 83
Secretoglobin family 3A member 2
SCGB3A2, P= 0.0254, M= 1.39, N= 83
Alpha−
FUT6, P= 0.0254, M= 1.09, N= 83
Platelet glycoprotein Ib alpha chain
GP1BA, P= 0.0254, M= 1.15, N= 83
Pregnancy zone protein
PZP, P= 0.0256, M= 1.12, N= 83
26S proteasome non−ATPase regulatory subunit 12
PSMD12, P= 0.0262, M= 1.25, N= 83
Protein THEMIS2
THEMIS2, P= 0.0264, M= 1.14, N= 83
Uncharacterized protein C1orf87
C1orf87, P= 0.0265, M= 1.29, N= 83
Calcium−activated chloride channel regulator 1
CLCA1, P= 0.0265, M= 1.4, N= 83
ADC
Carcinoid
LC
LCC
No
NSCL
SCC
SCC+ADC
SCLC
SCLC+NSCL

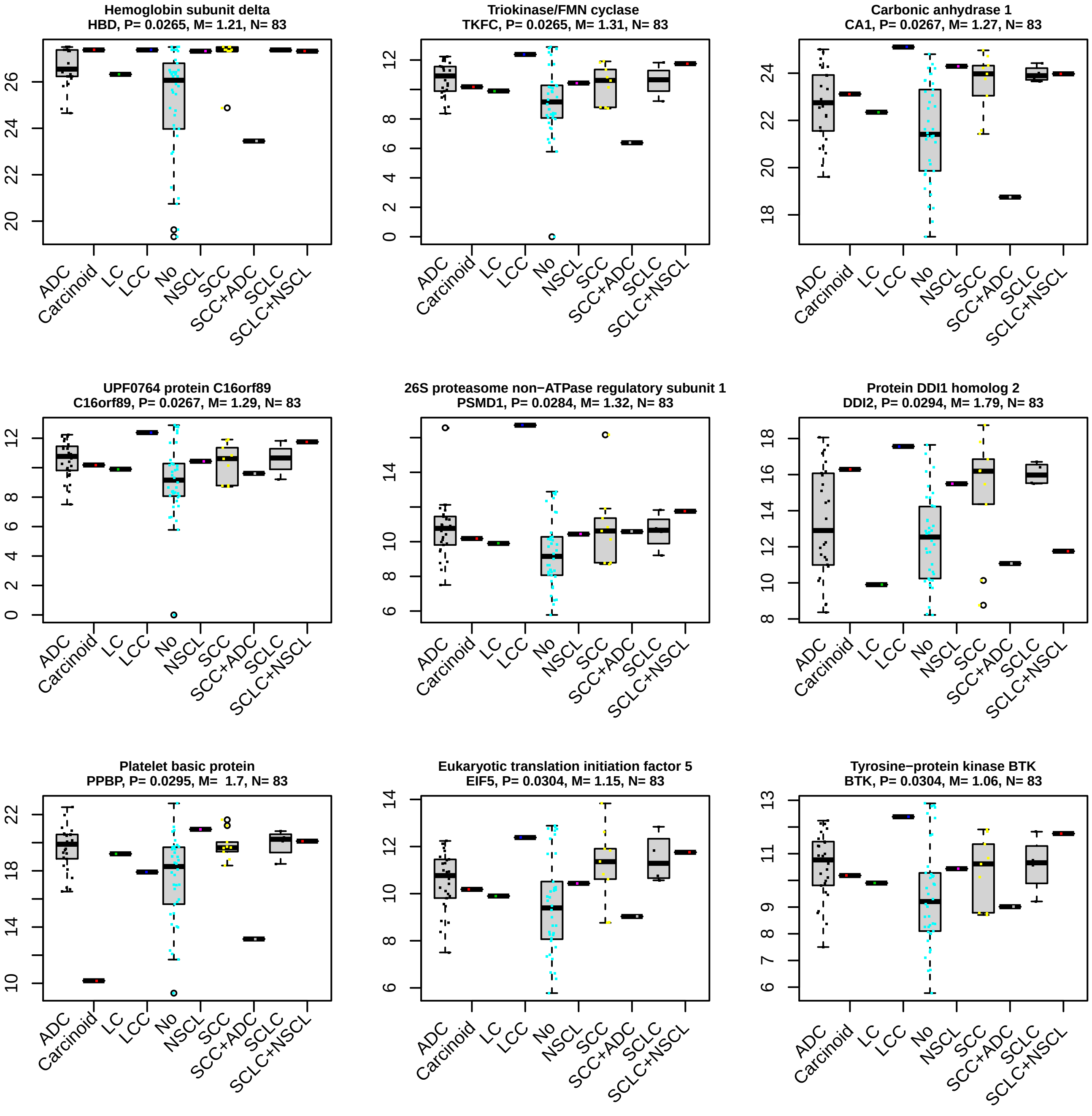
Hemoglobin subunit delta
HBD, P= 0.0265, M= 1.21, N= 83
Triokinase/FMN cyclase
TKFC, P= 0.0265, M= 1.31, N= 83
Carbonic anhydrase 1
CA1, P= 0.0267, M= 1.27, N= 83
UPF0764 protein C16orf89
C16orf89, P= 0.0267, M= 1.29, N= 83
26S proteasome non−ATPase regulatory subunit 1
PSMD1, P= 0.0284, M= 1.32, N= 83
Protein DDI1 homolog 2
DDI2, P= 0.0294, M= 1.79, N= 83
Platelet basic protein
PPBP, P= 0.0295, M= 1.7, N= 83
Eukaryotic translation initiation factor 5
EIF5, P= 0.0304, M= 1.15, N= 83
Tyrosine−protein kinase BTK
BTK, P= 0.0304, M= 1.06, N= 83
ADC
Carcinoid
LC
LCC
No
NSCL
SCC
SCC+ADC
SCLC
SCLC+NSCL

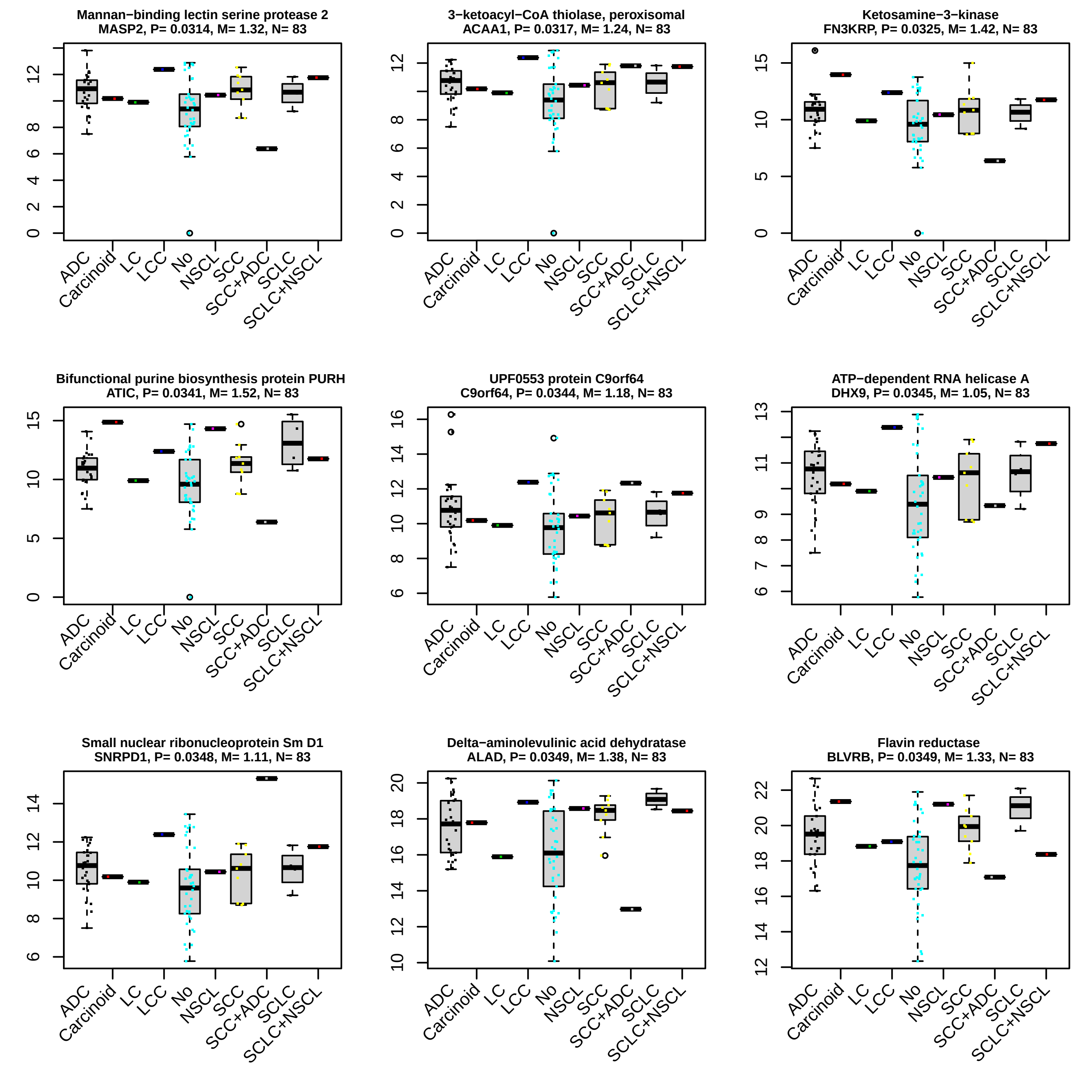

Mannan−binding lectin serine protease 2
MASP2, P= 0.0314, M= 1.32, N= 83
0 2 4 6 8 12
3−ketoacyl−CoA thiolase, peroxisomal
ACAA1, P= 0.0317, M= 1.24, N= 83
0 2 4 6 8 12
Ketosamine−3−kinase
FN3KRP, P= 0.0325, M= 1.42, N= 83
0 5 10 15
Bifunctional purine biosynthesis protein PURH
ATIC, P= 0.0341, M= 1.52, N= 83
0 5 10 15
UPF0553 protein C9orf64
C9orf64, P= 0.0344, M= 1.18, N= 83
6 8 10 12 14 16
ATP−dependent RNA helicase A
DHX9, P= 0.0345, M= 1.05, N= 83
6 7 8 9 11 13
Small nuclear ribonucleoprotein Sm D1
SNRPD1, P= 0.0348, M= 1.11, N= 83
6 8 10 12 14
Delta−aminolevulinic acid dehydratase
ALAD, P= 0.0349, M= 1.38, N= 83
10 12 14 16 18 20
Flavin reductase
BLVRB, P= 0.0349, M= 1.33, N= 83
12 14 16 18 20 22
ADC
Carcinoid
LC
LCC
No
NSCL
SCC
SCC+ADC
SCLC
SCLC+NSCL

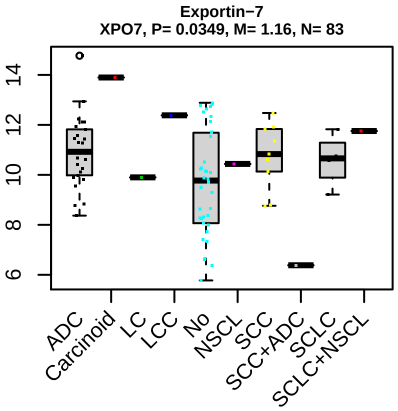
Exportin−7
XPO7, P= 0.0349, M= 1.16, N= 83
6
8
10
12
14
ADC
Carcinoid
LC
LCC
No
NSCL
SCC
SCC+ADC
SCLC
SCLC+NSCL

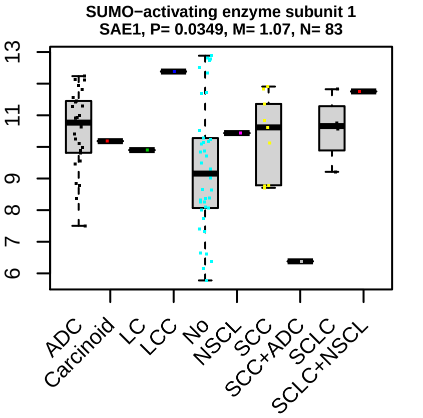
SUMO−activating enzyme subunit 1
SAE1, P= 0.0349, M= 1.07, N= 83
6
7
8
9
11
13
ADC
Carcinoid
LC
LCC
No
NSCL
SCC
SCC+ADC
SCLC
SCLC+NSCL

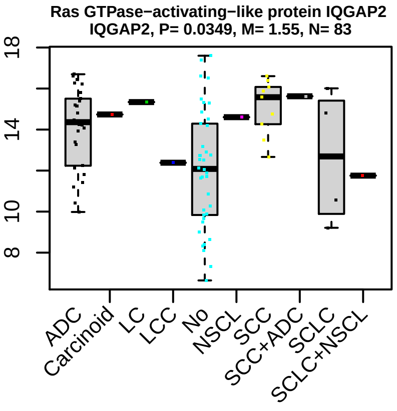
Ras GTPase−activating−like protein IQGAP2
IQGAP2, P= 0.0349, M= 1.55, N= 83
8
10
14
18
ADC
Carcinoid
LC
LCC
No
NSCL
SCC
SCC+ADC
SCLC
SCLC+NSCL

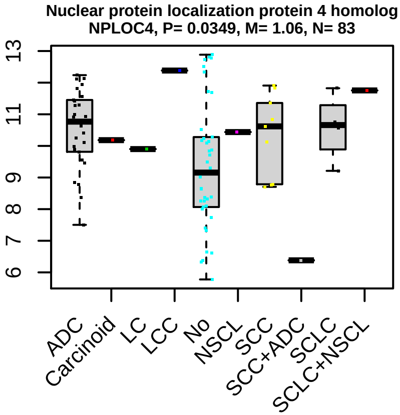
Nuclear protein localization protein 4 homolog
NPLOC4, P= 0.0349, M= 1.06, N= 83
6
7
8
9
11
13
ADC
Carcinoid
LC
LCC
No
NSCL
SCC
SCC+ADC
SCLC
SCLC+NSCL

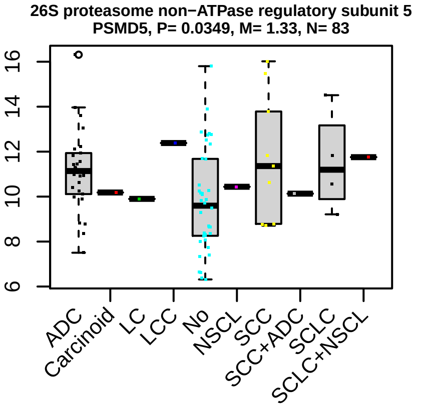
26S proteasome non−ATPase regulatory subunit 5
PSMD5, P= 0.0349, M= 1.33, N= 83
6
8
10
12
14
16
ADC
Carcinoid
LC
LCC
No
NSCL
SCC
SCC+ADC
SCLC
SCLC+NSCL

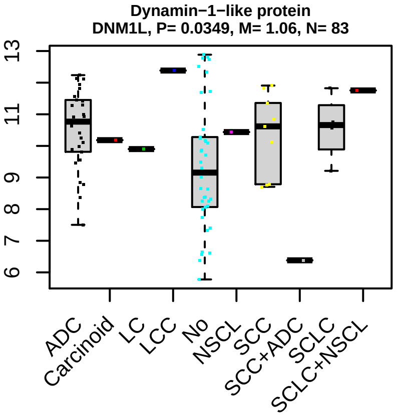
Dynamin−1−like protein
DNM1L, P= 0.0349, M= 1.06, N= 83
6
7
8
9
11
13
ADC
Carcinoid
LC
LCC
No
NSCL
SCC
SCC+ADC
SCLC
SCLC+NSCL

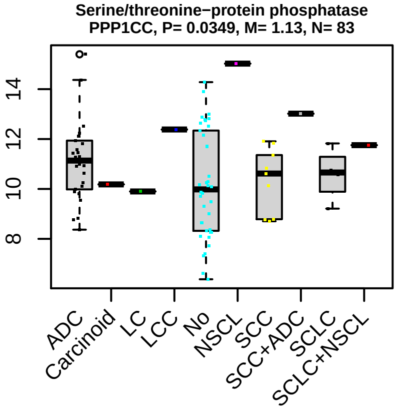
Serine/threonine−protein phosphatase
PPP1CC, P= 0.0349, M= 1.13, N= 83
8
10
12
14
ADC
Carcinoid
LC
LCC
No
NSCL
SCC
SCC+ADC
SCLC
SCLC+NSCL

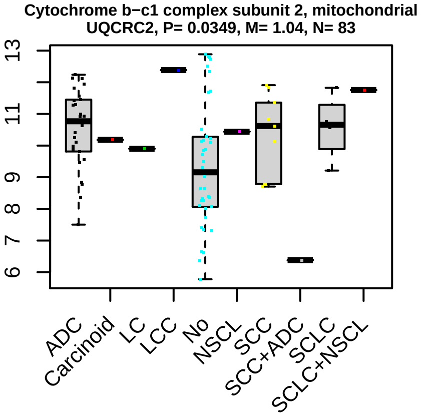
Cytochrome b−c1 complex subunit 2, mitochondrial
UQCRC2, P= 0.0349, M= 1.04, N= 83
6
7
8
9
11
13
ADC
Carcinoid
LC
LCC
No
NSCL
SCC
SCC+ADC
SCLC
SCLC+NSCL

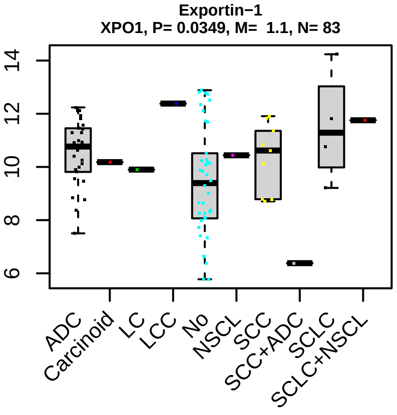
Exportin−1
XPO1, P= 0.0349, M= 1.1, N= 83
6
8
10
12
14
ADC
Carcinoid
LC
LCC
No
NSCL
SCC
SCC+ADC
SCLC
SCLC+NSCL

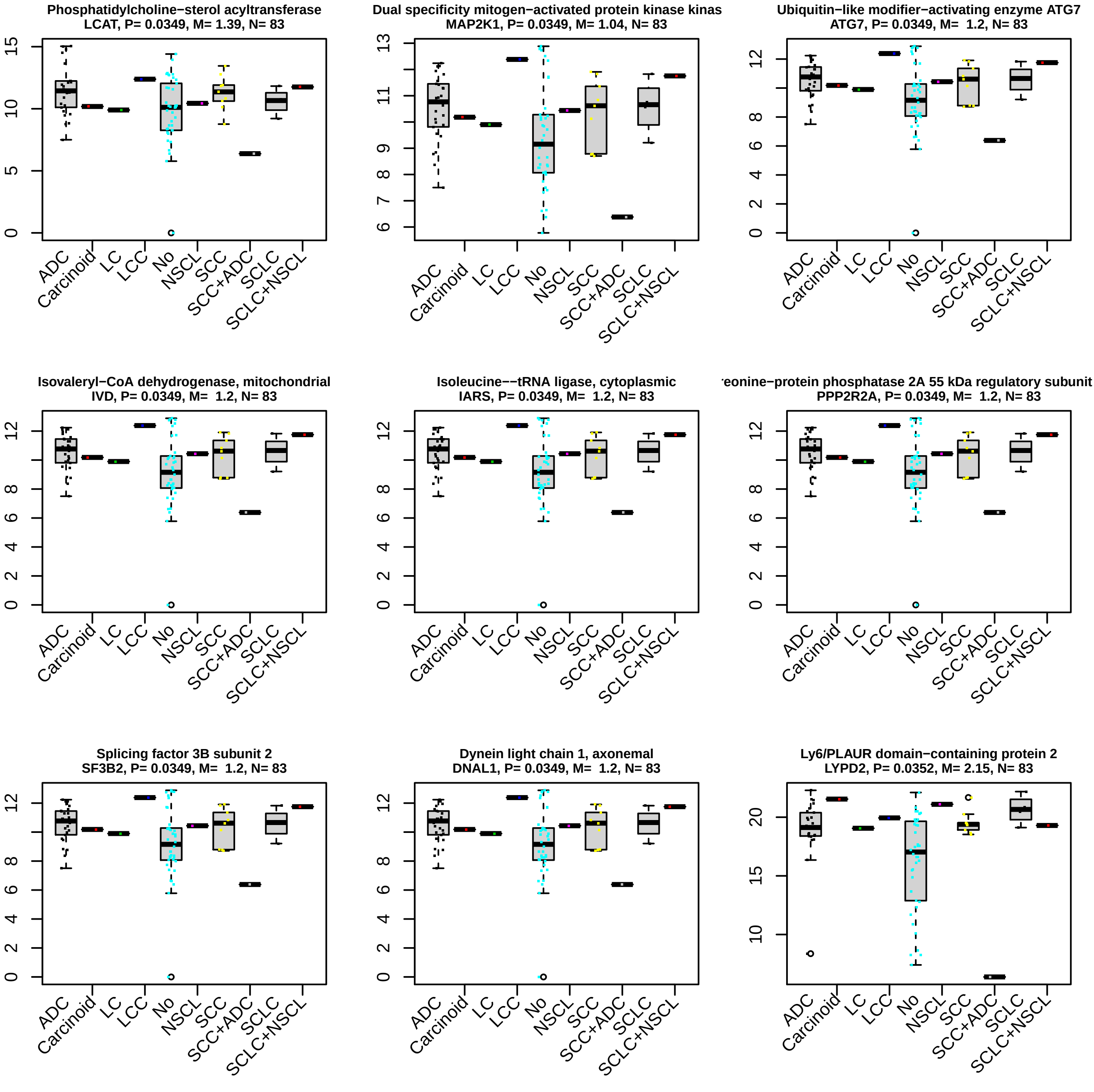

Phosphatidylcholine−sterol acyltransferase
LCAT, P= 0.0349, M= 1.39, N= 83
Dual specificity mitogen−activated protein kinase kinas
MAP2K1, P= 0.0349, M= 1.04, N= 83
Ubiquitin−like modifier−activating enzyme ATG7
ATG7, P= 0.0349, M= 1.2, N= 83
Isovaleryl−CoA dehydrogenase, mitochondrial
IVD, P= 0.0349, M= 1.2, N= 83
Isoleucine−−tRNA ligase, cytoplasmic
IARS, P= 0.0349, M= 1.2, N= 83
eonine−protein phosphatase 2A 55 kDa regulatory subunit
PPP2R2A, P= 0.0349, M= 1.2, N= 83
Splicing factor 3B subunit 2
SF3B2, P= 0.0349, M= 1.2, N= 83
Dynein light chain 1, axonemal
DNAL1, P= 0.0349, M= 1.2, N= 83
Ly6/PLAUR domain−containing protein 2
LYPD2, P= 0.0352, M= 2.15, N= 83
ADC
Carcinoid
LC
LCC
No
NSCL
SCC
SCC+ADC
SCLC
SCLC+NSCL

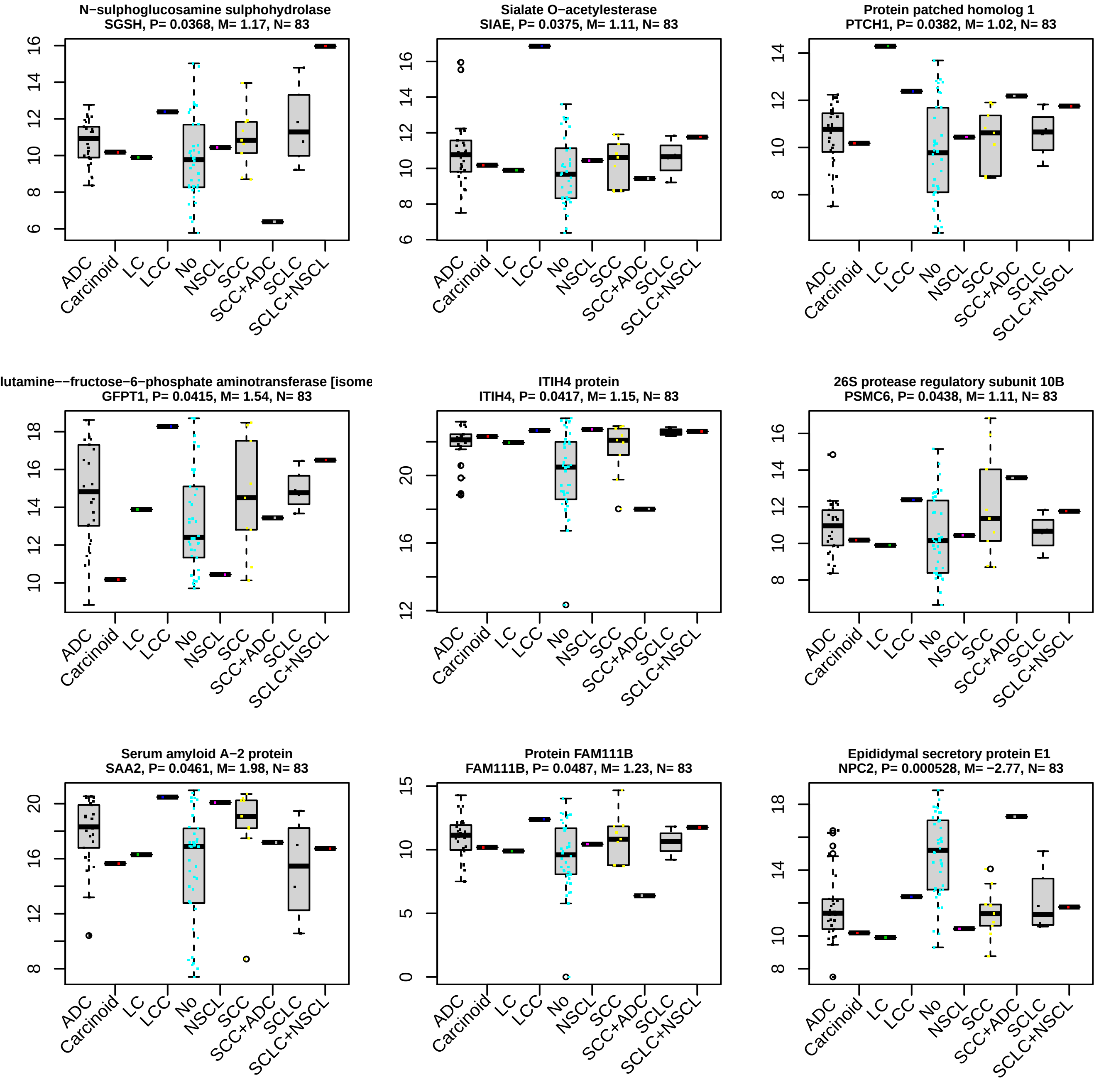
N−sulphoglucosamine sulphohydrolase
SGSH, P= 0.0368, M= 1.17, N= 83
Sialate O−acetylesterase
SIAE, P= 0.0375, M= 1.11, N= 83
Protein patched homolog 1
PTCH1, P= 0.0382, M= 1.02, N= 83
lutamine−−fructose−6−phosphate aminotransferase [isome
GFPT1, P= 0.0415, M= 1.54, N= 83
ITIH4 protein
ITIH4, P= 0.0417, M= 1.15, N= 83
26S protease regulatory subunit 10B
PSMC6, P= 0.0438, M= 1.11, N= 83
Serum amyloid A−2 protein
SAA2, P= 0.0461, M= 1.98, N= 83
Protein FAM111B
FAM111B, P= 0.0487, M= 1.23, N= 83
Epididymal secretory protein E1
NPC2, P= 0.000528, M= −2.77, N= 83
ADC
Carcinoid
LC
LCC
No
NSCL
SCC
SCC+ADC
SCLC
SCLC+NSCL

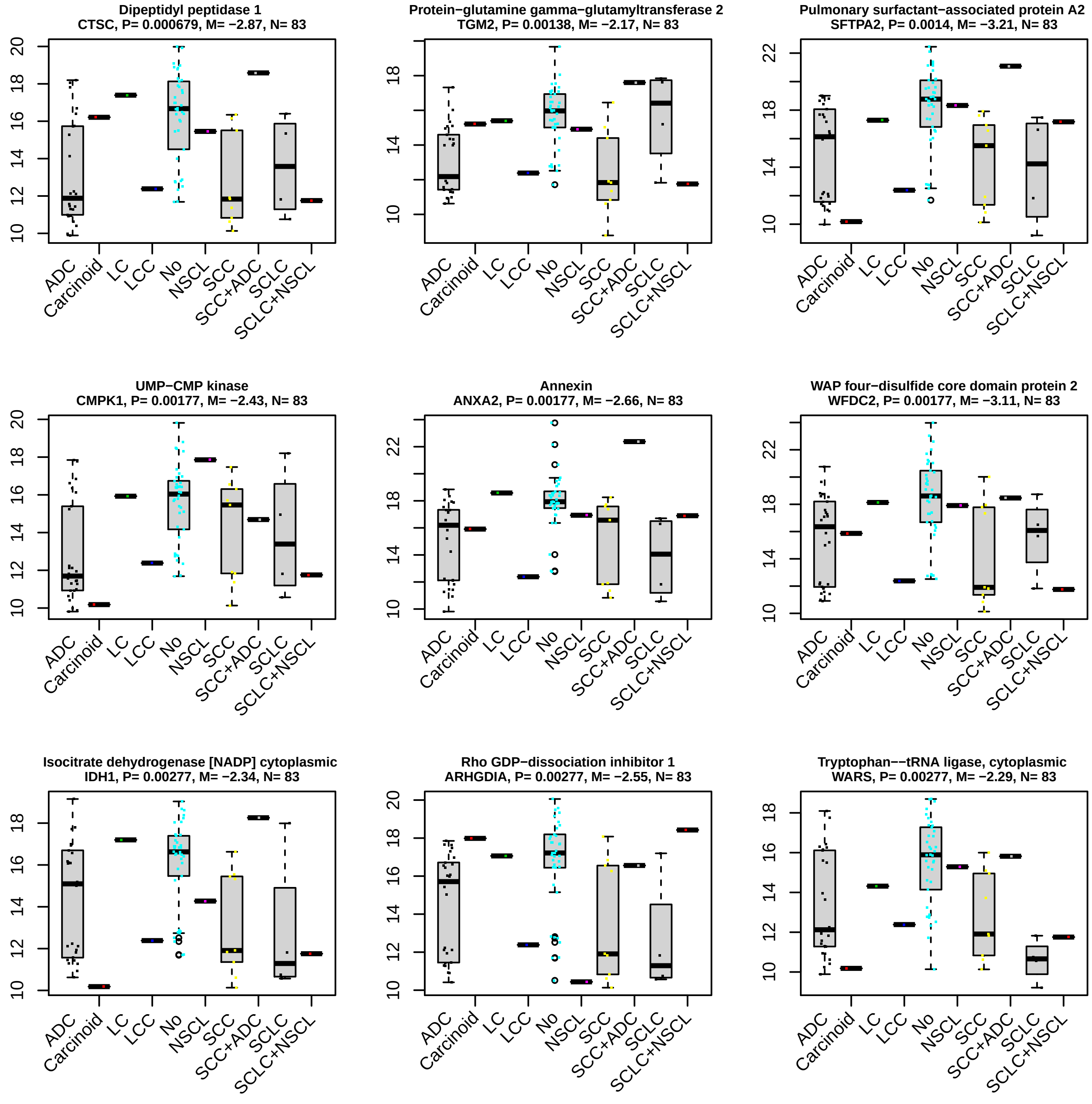

Dipeptidyl peptidase 1
CTSC, P= 0.000679, M= −2.87, N= 83
10 12 14 16 18 20
ADC Carcinoid LC LCC No NSCL SCC SCC+ADC SCLC SCLC+NSCL
Protein−glutamine gamma−glutamyltransferase 2
TGM2, P= 0.00138, M= −2.17, N= 83
10 14 18
ADC Carcinoid LC LCC No NSCL SCC SCC+ADC SCLC SCLC+NSCL
Pulmonary surfactant−associated protein A2
SFTPA2, P= 0.0014, M= −3.21, N= 83
10 14 18 22
ADC Carcinoid LC LCC No NSCL SCC SCC+ADC SCLC SCLC+NSCL
UMP−CMP kinase
CMPK1, P= 0.00177, M= −2.43, N= 83
10 12 14 16 18 20
ADC Carcinoid LC LCC No NSCL SCC SCC+ADC SCLC SCLC+NSCL
Annexin
ANXA2, P= 0.00177, M= −2.66, N= 83
10 14 18 22
ADC Carcinoid LC LCC No NSCL SCC SCC+ADC SCLC SCLC+NSCL
WAP four−disulfide core domain protein 2
WFDC2, P= 0.00177, M= −3.11, N= 83
10 14 18 22
ADC Carcinoid LC LCC No NSCL SCC SCC+ADC SCLC SCLC+NSCL
Isocitrate dehydrogenase [NADP] cytoplasmic
IDH1, P= 0.00277, M= −2.34, N= 83
10 12 14 16 18
ADC Carcinoid LC LCC No NSCL SCC SCC+ADC SCLC SCLC+NSCL
Rho GDP−dissociation inhibitor 1
ARHGDIA, P= 0.00277, M= −2.55, N= 83
10 12 14 16 18 20
ADC Carcinoid LC LCC No NSCL SCC SCC+ADC SCLC SCLC+NSCL
Tryptophan−−tRNA ligase, cytoplasmic
WARS, P= 0.00277, M= −2.29, N= 83
10 12 14 16 18
ADC Carcinoid LC LCC No NSCL SCC SCC+ADC SCLC SCLC+NSCL

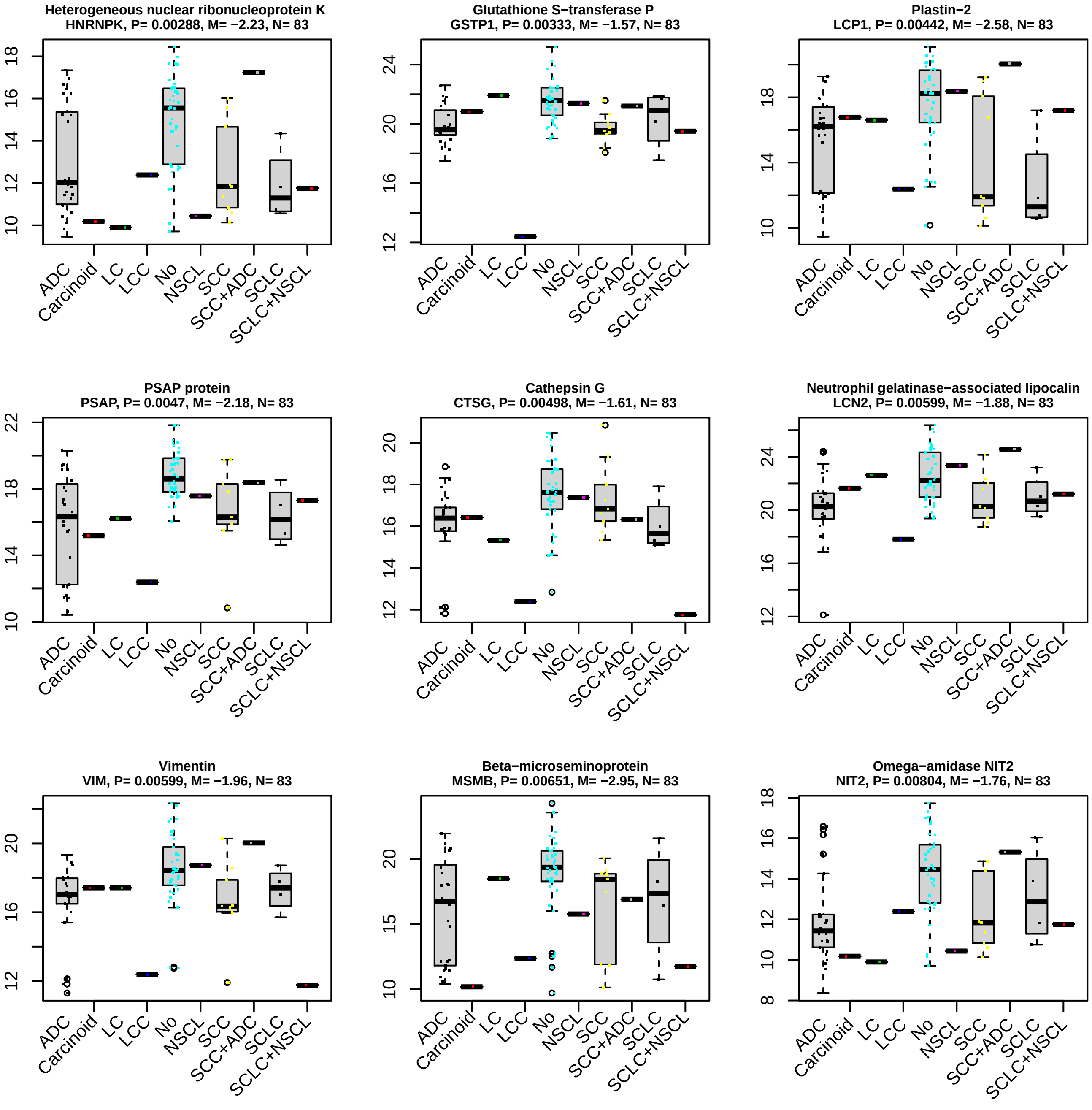
Heterogeneous nuclear ribonucleoprotein K
HNRNPK, P= 0.00288, M= −2.23, N= 83
Glutathione S−transferase P
GSTP1, P= 0.00333, M= −1.57, N= 83
Plastin−2
LCP1, P= 0.00442, M= −2.58, N= 83
PSAP protein
PSAP, P= 0.0047, M= −2.18, N= 83
Cathepsin G
CTSG, P= 0.00498, M= −1.61, N= 83
Neutrophil gelatinase−associated lipocalin
LCN2, P= 0.00599, M= −1.88, N= 83
Vimentin
VIM, P= 0.00599, M= −1.96, N= 83
Beta−microseminoprotein
MSMB, P= 0.00651, M= −2.95, N= 83
Omega−amidase NIT2
NIT2, P= 0.00804, M= −1.76, N= 83
ADC
Carcinoid
LC
LCC
No
NSCL
SCC
SCC+ADC
SCLC
SCLC+NSCL

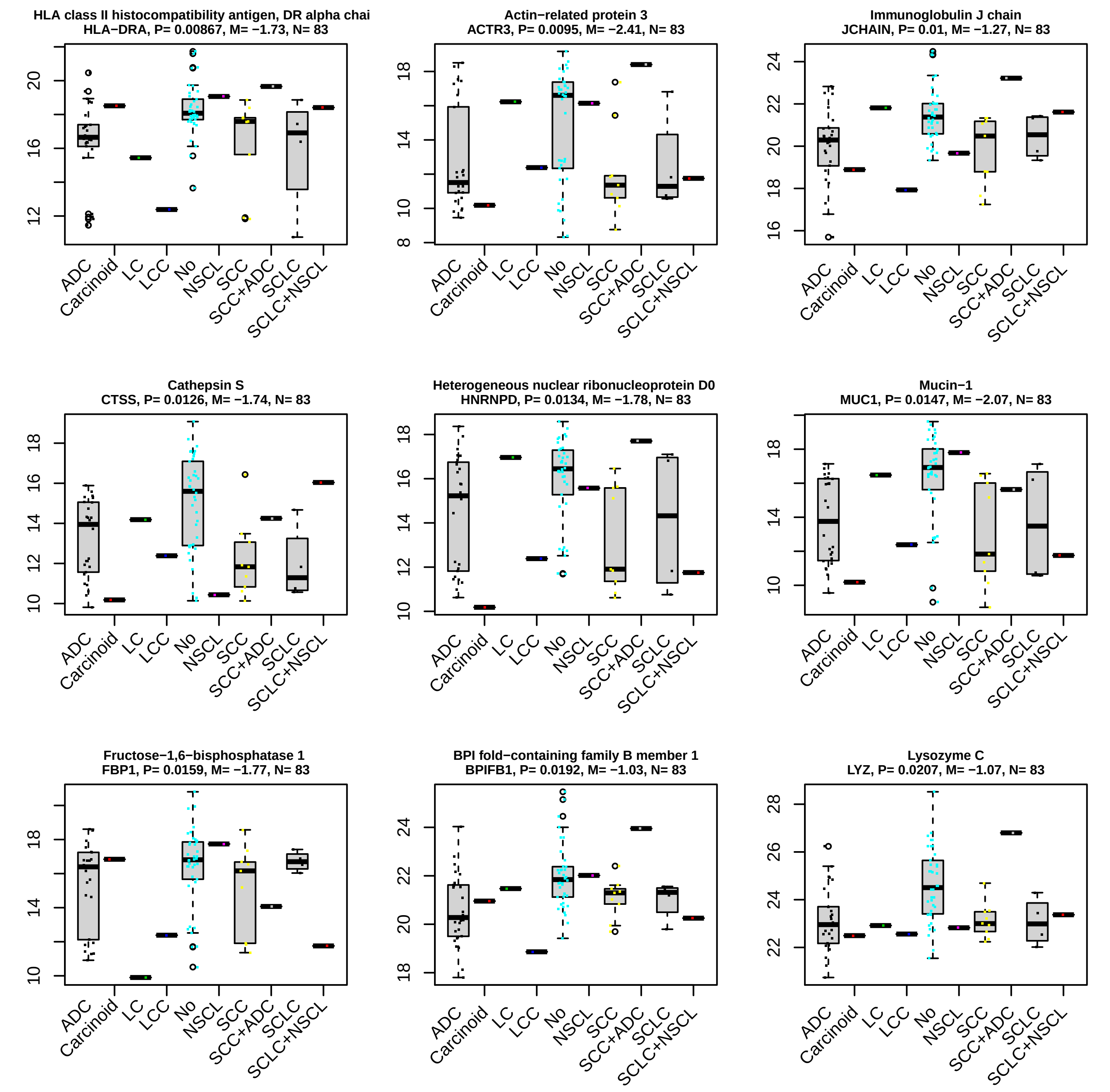
HLA class II histocompatibility antigen, DR alpha chai
HLA−DRA, P= 0.00867, M= −1.73, N= 83
12
16
20
ADC
Carcinoid
LC
LCC
No
NSCL
SCC
SCC+ADC
SCLC
SCLC+NSCL
Actin−related protein 3
ACTR3, P= 0.0095, M= −2.41, N= 83
8
10
14
18
ADC
Carcinoid
LC
LCC
No
NSCL
SCC
SCC+ADC
SCLC
SCLC+NSCL
Immunoglobulin J chain
JCHAIN, P= 0.01, M= −1.27, N= 83
16
18
20
22
24
ADC
Carcinoid
LC
LCC
No
NSCL
SCC
SCC+ADC
SCLC
SCLC+NSCL
Cathepsin S
CTSS, P= 0.0126, M= −1.74, N= 83
10
12
14
16
18
ADC
Carcinoid
LC
LCC
No
NSCL
SCC
SCC+ADC
SCLC
SCLC+NSCL
Heterogeneous nuclear ribonucleoprotein D0
HNRNPD, P= 0.0134, M= −1.78, N= 83
10
12
14
16
18
ADC
Carcinoid
LC
LCC
No
NSCL
SCC
SCC+ADC
SCLC
SCLC+NSCL
Mucin−1
MUC1, P= 0.0147, M= −2.07, N= 83
10
14
18
ADC
Carcinoid
LC
LCC
No
NSCL
SCC
SCC+ADC
SCLC
SCLC+NSCL
Fructose−1,6−bisphosphatase 1
FBP1, P= 0.0159, M= −1.77, N= 83
10
14
18
ADC
Carcinoid
LC
LCC
No
NSCL
SCC
SCC+ADC
SCLC
SCLC+NSCL
BPI fold−containing family B member 1
BPIFB1, P= 0.0192, M= −1.03, N= 83
18
20
22
24
ADC
Carcinoid
LC
LCC
No
NSCL
SCC
SCC+ADC
SCLC
SCLC+NSCL
Lysozyme C
LYZ, P= 0.0207, M= −1.07, N= 83
22
24
26
28
ADC
Carcinoid
LC
LCC
No
NSCL
SCC
SCC+ADC
SCLC
SCLC+NSCL

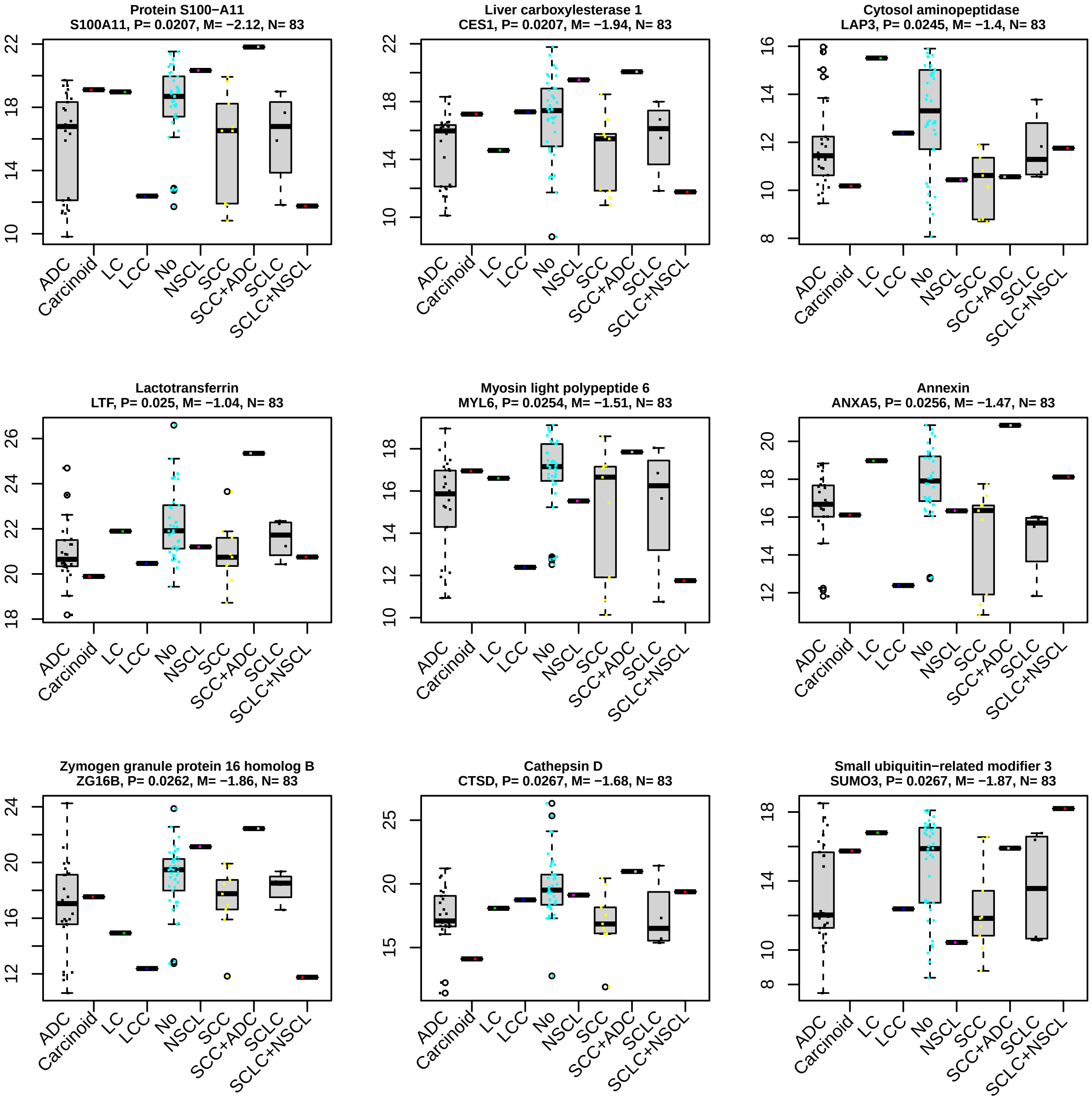
Protein S100−A11
S100A11, P= 0.0207, M= −2.12, N= 83
10 14 18 22
Liver carboxylesterase 1
CES1, P= 0.0207, M= −1.94, N= 83
10 14 18 22
Cytosol aminopeptidase
LAP3, P= 0.0245, M= −1.4, N= 83
8 10 12 14 16
Lactotransferrin
LTF, P= 0.025, M= −1.04, N= 83
18 20 22 24 26
Myosin light polypeptide 6
MYL6, P= 0.0254, M= −1.51, N= 83
10 12 14 16 18
Annexin
ANXA5, P= 0.0256, M= −1.47, N= 83
12 14 16 18 20
Zymogen granule protein 16 homolog B
ZG16B, P= 0.0262, M= −1.86, N= 83
12 16 20 24
Cathepsin D
CTSD, P= 0.0267, M= −1.68, N= 83
15 20 25
Small ubiquitin−related modifier 3
SUMO3, P= 0.0267, M= −1.87, N= 83
8 10 14 18
ADC
Carcinoid
LC
LCC
No
NSCL
SCC
SCC+ADC
SCLC
SCLC+NSCL

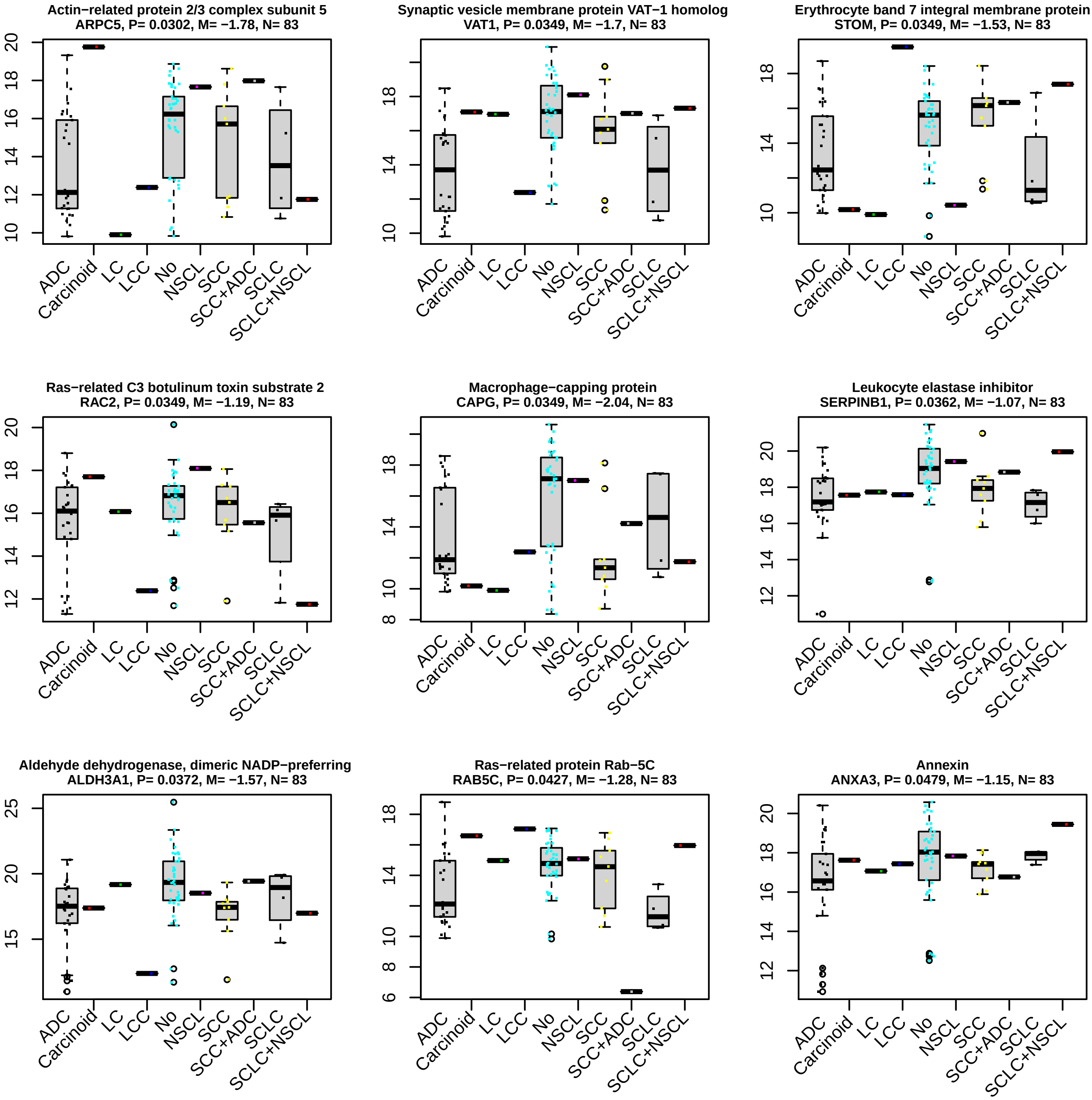

Actin−related protein 2/3 complex subunit 5
ARPC5, P= 0.0302, M= −1.78, N= 83
Synaptic vesicle membrane protein VAT−1 homolog
VAT1, P= 0.0349, M= −1.7, N= 83
Erythrocyte band 7 integral membrane protein
STOM, P= 0.0349, M= −1.53, N= 83
Ras−related C3 botulinum toxin substrate 2
RAC2, P= 0.0349, M= −1.19, N= 83
Macrophage−capping protein
CAPG, P= 0.0349, M= −2.04, N= 83
Leukocyte elastase inhibitor
SERPINB1, P= 0.0362, M= −1.07, N= 83
Aldehyde dehydrogenase, dimeric NADP−preferring
ALDH3A1, P= 0.0372, M= −1.57, N= 83
Ras−related protein Rab−5C
RAB5C, P= 0.0427, M= −1.28, N= 83
Annexin
ANXA3, P= 0.0479, M= −1.15, N= 83
ADC
Carcinoid
LC
LCC
No
NSCL
SCC
SCC+ADC
SCLC
SCLC+NSCL

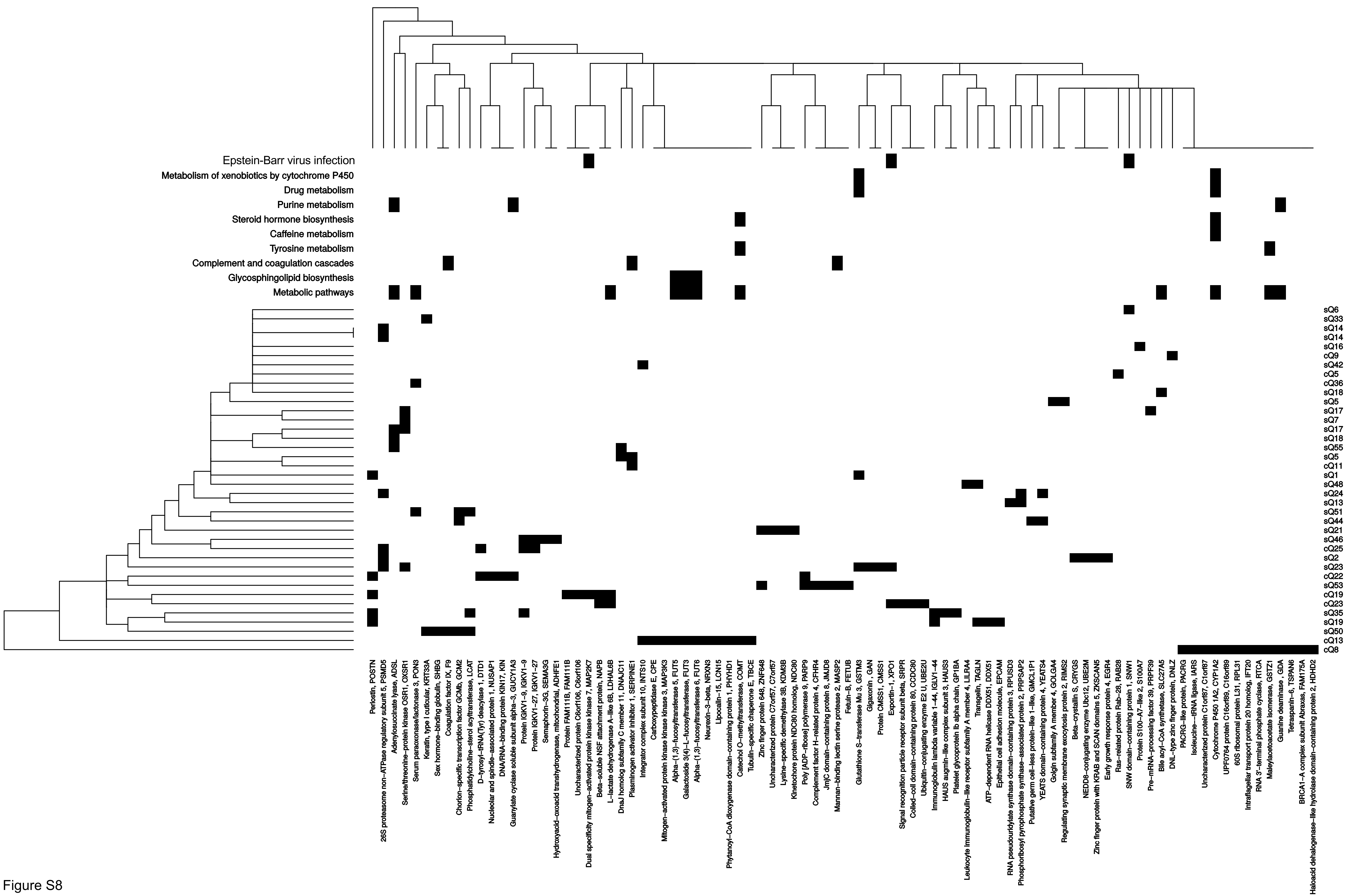

Epstein-Barr virus infection
Metabolism of xenobiotics by cytochrome P450
Drug metabolism
Purine metabolism
Steroid hormone biosynthesis
Caffeine metabolism
Tyrosine metabolism
Complement and coagulation cascades
Glycosphingolipid biosynthesis
Metabolic pathways
sQ6
sQ33
sQ14
sQ14
sQ16
cQ9
sQ42
cQ5
cQ36
sQ18
sQ5
sQ17
sQ7
sQ17
sQ18
sQ55
sQ5
cQ11
sQ1
sQ48
sQ24
sQ13
sQ51
sQ44
sQ21
sQ46
cQ25
sQ2
sQ23
cQ22
sQ53
cQ19
cQ23
sQ35
sQ19
sQ50
cQ13
cQ8

Figure S8

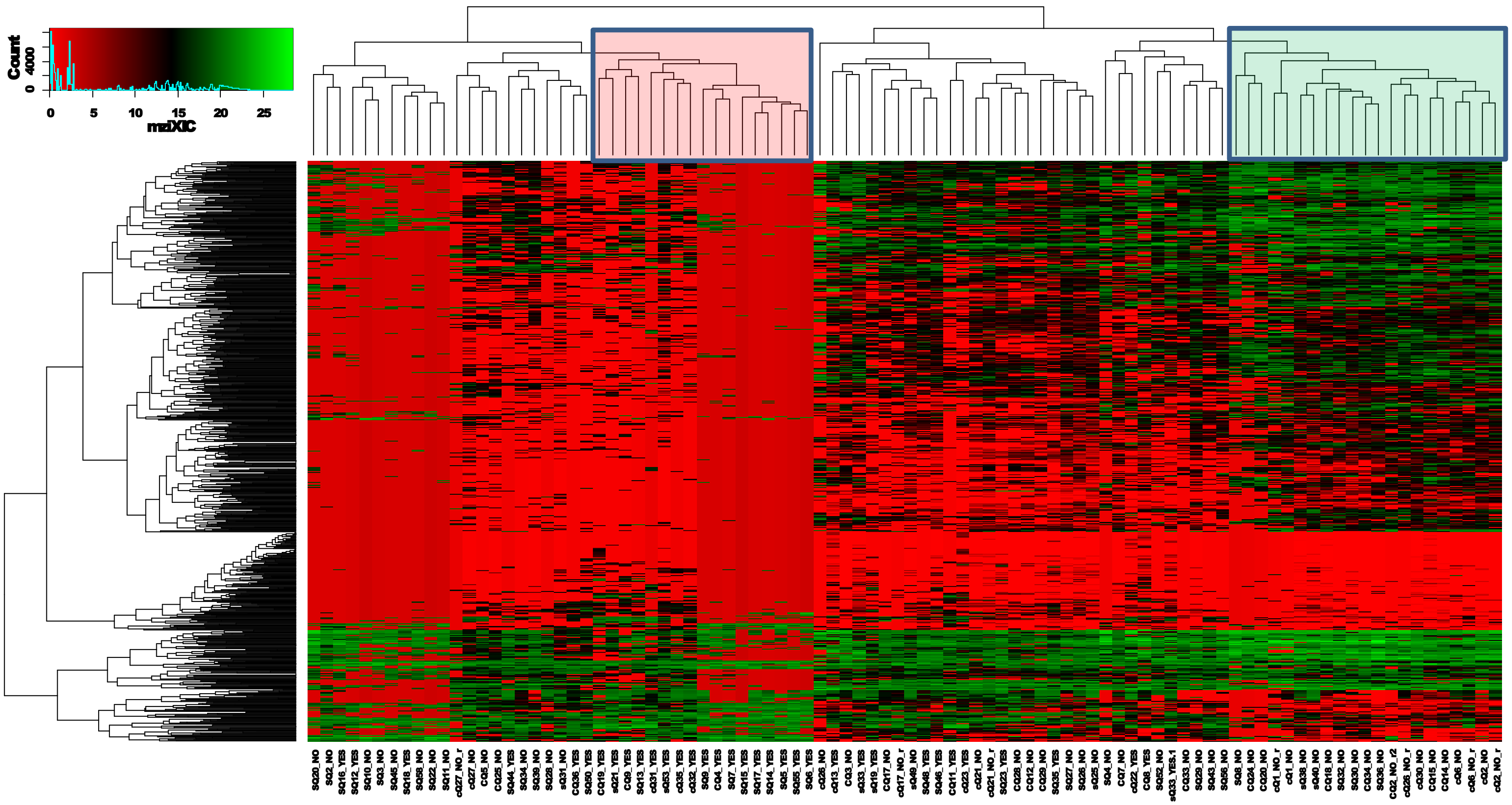

Figure S9

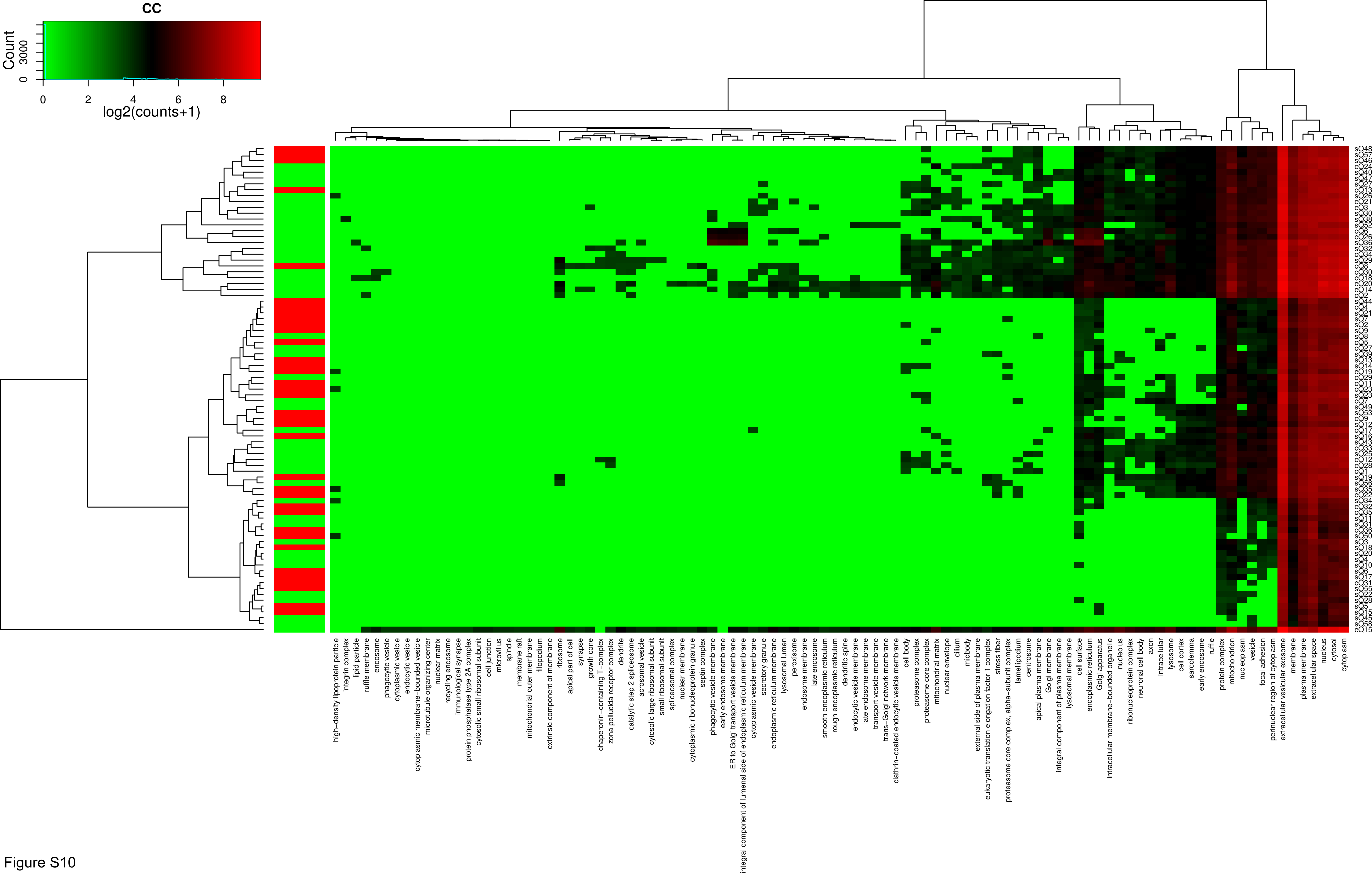

Figure S10

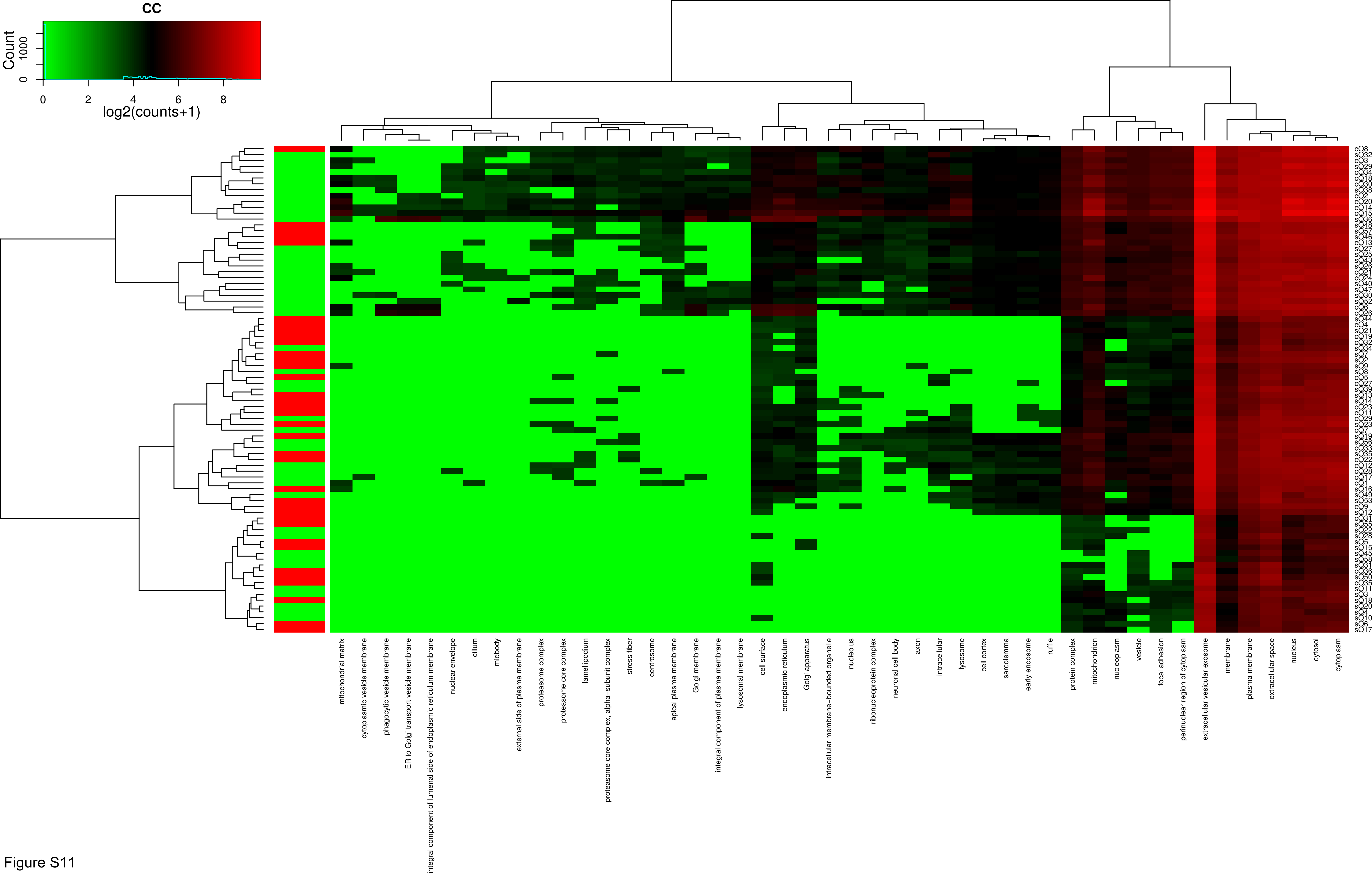

CC
Count
0
1000
0
2
4
6
8
log2(counts+1)
mitochondrial matrix
cytoplasmic vesicle membrane
phagocytic vesicle membrane
ER to Golgi transport vesicle membrane
integral component of lumenal side of endoplasmic reticulum membrane
nuclear envelope
cilium
midbody
external side of plasma membrane
proteasome complex
proteasome core complex
lamellipodium
proteasome core complex, alpha−subunit complex
stress fiber
centrosome
apical plasma membrane
Golgi membrane
integral component of plasma membrane
lysosomal membrane
cell surface
endoplasmic reticulum
Golgi apparatus
intracellular membrane−bounded organelle
nucleolus
ribonucleoprotein complex
neuronal cell body
axon
intracellular
lysosome
cell cortex
sarcolemma
early endosome
ruffle
protein complex
mitochondrion
nucleoplasm
vesicle
focal adhesion
perinuclear region of cytoplasm
extracellular vesicular exosome
membrane
plasma membrane
extracellular space
nucleus
cytosol
cytoplasm
cQ8
sQ32
cQ3
sQ29
cQ34
cQ18
cQ30
sQ38
cQ2
cQ20
cQ14
cQ15
sQ36
sQ48
sQ57
sQ46
cQ13
sQ27
sQ25
sQ43
sQ26
cQ21
cQ24
sQ40
sQ47
sQ30
sQ52
cQ6
cQ26
sQ44
cQ4
sQ21
cQ19
cQ32
sQ34
sQ7
sQ2
sQ9
sQ8
cQ5
cQ27
sQ39
sQ13
sQ14
cQ23
cQ11
cQ29
sQ23
cQ7
sQ19
sQ56
cQ33
sQ35
cQ22
cQ12
cQ28
cQ17
cQ1
sQ16
sQ49
sQ53
cQ9
sQ12
cQ31
sQ55
sQ22
sQ28
sQ5
sQ15
sQ45
sQ58
sQ31
cQ36
sQ50
cQ35
sQ11
sQ3
sQ18
sQ20
sQ4
sQ10
sQ6
sQ17

Figure S11

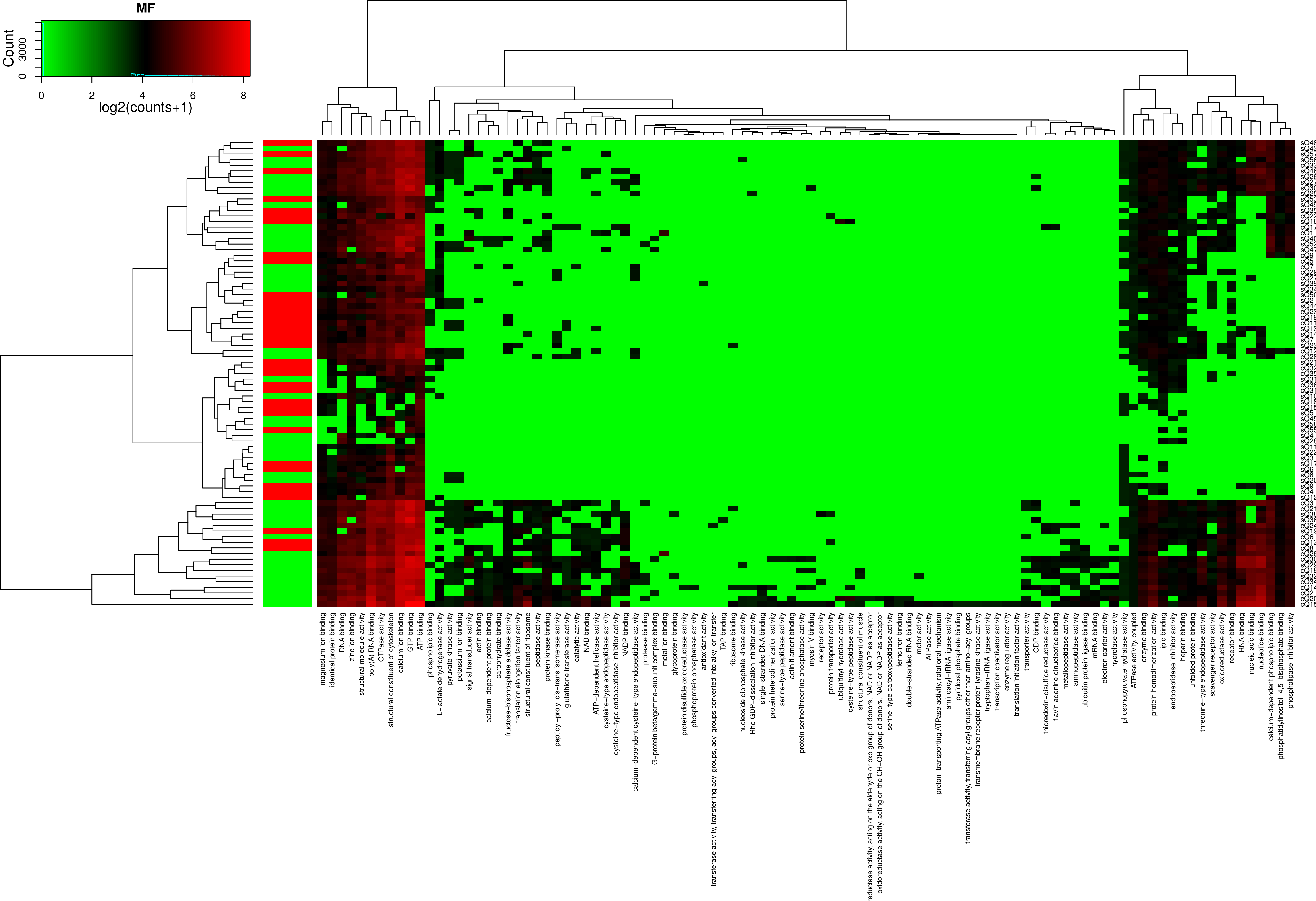
MF
Count
0
3000
0
2
4
6
8
log2(counts+1)
magnesium ion binding
identical protein binding
DNA binding
zinc ion binding
structural molecule activity
poly(A) RNA binding
GTPase activity
structural constituent of cytoskeleton
calcium ion binding
GTP binding
ATP binding
phospholipid binding
L−lactate dehydrogenase activity
pyruvate kinase activity
potassium ion binding
signal transducer activity
actin binding
calcium−dependent protein binding
carbohydrate binding
fructose−bisphosphate aldolase activity
translation elongation factor activity
structural constituent of ribosome
peptidase activity
protein kinase binding
peptidyl−prolyl cis−trans isomerase activity
glutathione transferase activity
catalytic activity
NAD binding
ATP−dependent helicase activity
cysteine−type endopeptidase activity
cysteine−type endopeptidase inhibitor activity
NADP binding
calcium−dependent cysteine−type endopeptidase activity
protease binding
G−protein beta/gamma−subunit complex binding
metal ion binding
glycoprotein binding
protein disulfide oxidoreductase activity
phosphoprotein phosphatase activity
antioxidant activity
transferase activity, transferring acyl groups, acyl groups converted into alkyl on transfer
TAP binding
ribosome binding
nucleoside diphosphate kinase activity
Rho GDP−dissociation inhibitor activity
single−stranded DNA binding
protein heterodimerization activity
serine−type peptidase activity
actin filament binding
protein serine/threonine phosphatase activity
myosin V binding
receptor activity
protein transporter activity
ubiquitinyl hydrolase activity
cysteine−type peptidase activity
structural constituent of muscle
oxidoreductase activity, acting on the aldehyde or oxo group of donors, NAD or NADP as acceptor
oxidoreductase activity, acting on the CH−OH group of donors, NAD or NADP as acceptor
serine−type carboxypeptidase activity
ferric iron binding
double−stranded RNA binding
motor activity
ATPase activity
proton−transporting ATPase activity, rotational mechanism
aminoacyl−tRNA ligase activity
pyridoxal phosphate binding
transferase activity, transferring acyl groups other than amino−acyl groups
transmembrane receptor protein tyrosine kinase activity
tryptophan−tRNA ligase activity
transcription coactivator activity
enzyme regulator activity
translation initiation factor activity
transporter activity
GDP binding
thioredoxin−disulfide reductase activity
flavin adenine dinucleotide binding
metallopeptidase activity
aminopeptidase activity
ubiquitin protein ligase binding
mRNA binding
electron carrier activity
hydrolase activity
phosphopyruvate hydratase activity
ATPase activity, coupled
enzyme binding
protein homodimerization activity
lipid binding
endopeptidase inhibitor activity
heparin binding
unfolded protein binding
threonine−type endopeptidase activity
scavenger receptor activity
oxidoreductase activity
receptor binding
RNA binding
nucleic acid binding
nucleotide binding
calcium−dependent phospholipid binding
phosphatidylinositol−4,5−bisphosphate binding
phospholipase inhibitor activity
Figure S12

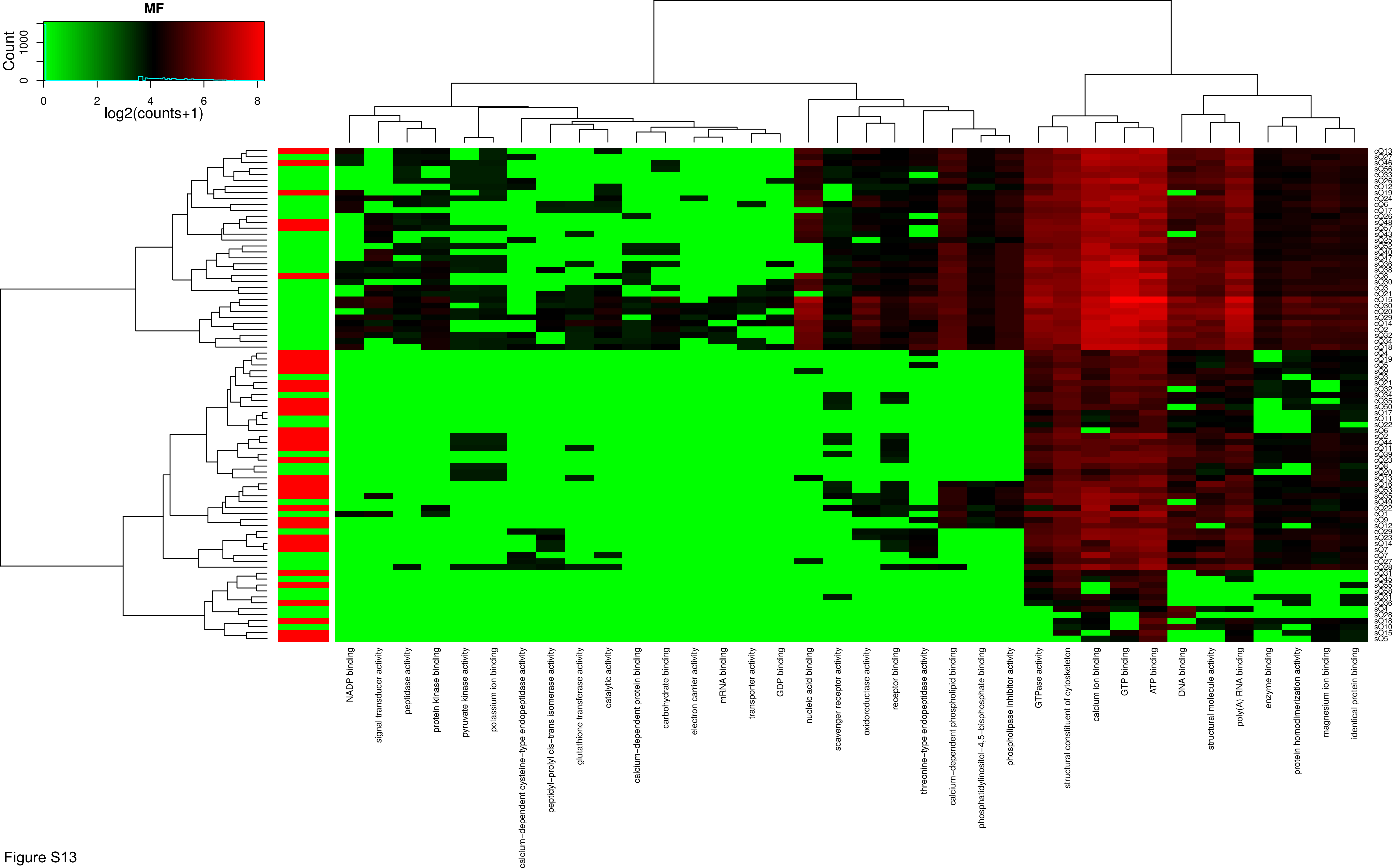
MF
Count
0
1000
0
2
4
6
8
log2(counts+1)
NADP binding
signal transducer activity
peptidase activity
protein kinase binding
pyruvate kinase activity
potassium ion binding
calcium−dependent cysteine−type endopeptidase activity
peptidyl−prolyl cis−trans isomerase activity
glutathione transferase activity
catalytic activity
calcium−dependent protein binding
carbohydrate binding
electron carrier activity
mRNA binding
transporter activity
GDP binding
nucleic acid binding
scavenger receptor activity
oxidoreductase activity
receptor binding
threonine−type endopeptidase activity
calcium−dependent phospholipid binding
phosphatidylinositol−4,5−bisphosphate binding
phospholipase inhibitor activity
GTPase activity
structural constituent of cytoskeleton
calcium ion binding
GTP binding
ATP binding
DNA binding
structural molecule activity
poly(A) RNA binding
enzyme binding
protein homodimerization activity
magnesium ion binding
identical protein binding

Figure S13

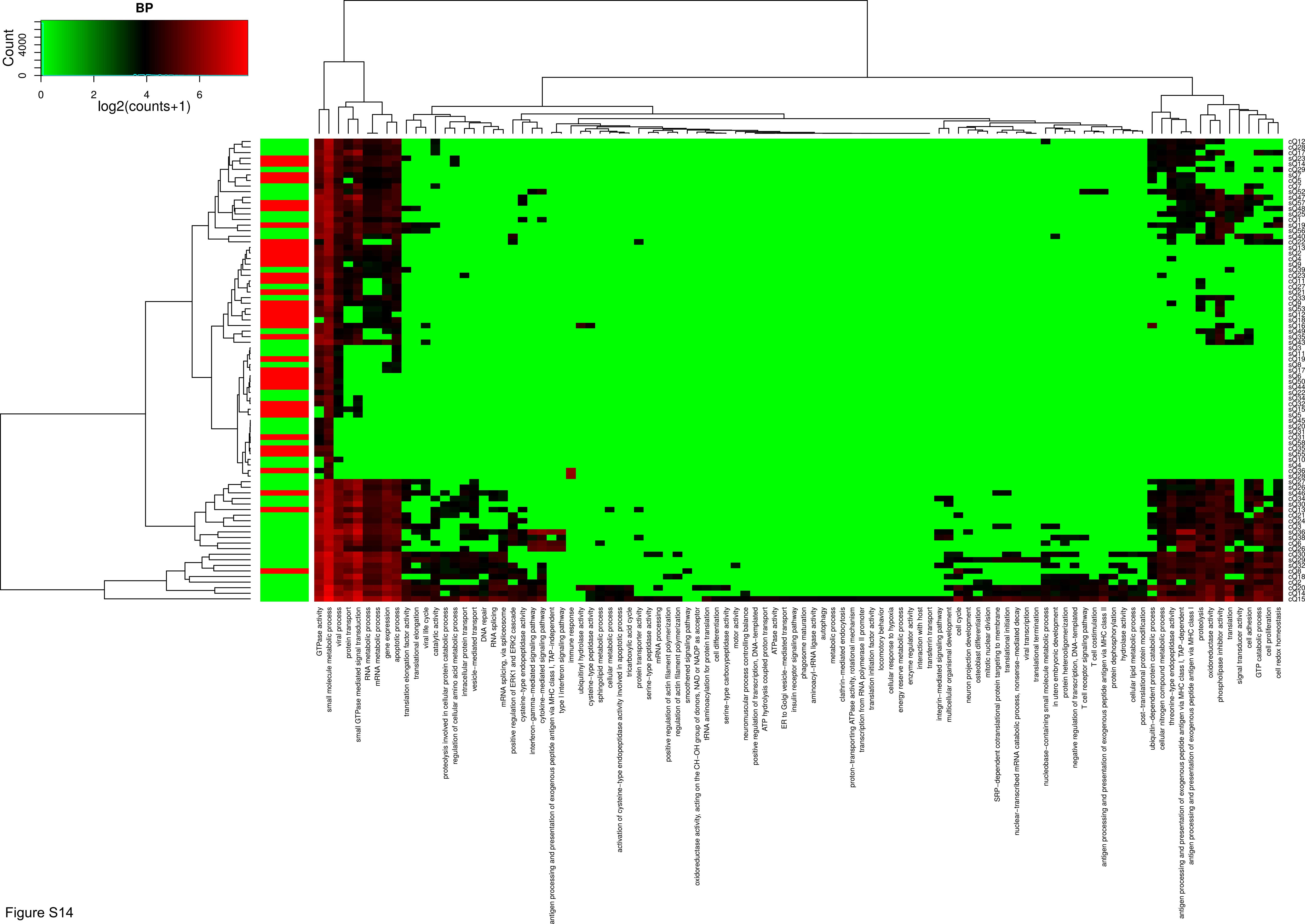
BP
Count
0
4000
0
2
4
6
log2(counts+1)
GTPase activity
small molecule metabolic process
viral process
protein transport
small GTPase mediated signal transduction
RNA metabolic process
mRNA metabolic process
gene expression
apoptotic process
translation elongation factor activity
translational elongation
viral life cycle
catalytic activity
proteolysis involved in cellular protein catabolic process
regulation of cellular amino acid metabolic process
intracellular protein transport
vesicle−mediated transport
DNA repair
RNA splicing
mRNA splicing, via spliceosome
positive regulation of ERK1 and ERK2 cascade
cysteine−type endopeptidase activity
interferon−gamma−mediated signaling pathway
cytokine−mediated signaling pathway
antigen processing and presentation of exogenous peptide antigen via MHC class I, TAP−independent
type I interferon signaling pathway
immune response
ubiquitinyl hydrolase activity
cysteine−type peptidase activity
sphingolipid metabolic process
cellular metabolic process
activation of cysteine−type endopeptidase activity involved in apoptotic process
tricarboxylic acid cycle
protein transporter activity
serine−type peptidase activity
mRNA processing
positive regulation of actin filament polymerization
regulation of actin filament polymerization
smoothened signaling pathway
oxidoreductase activity, acting on the CH−OH group of donors, NAD or NADP as acceptor
tRNA aminoacylation for protein translation
cell differentiation
serine−type carboxypeptidase activity
motor activity
neuromuscular process controlling balance
positive regulation of transcription, DNA−templated
ATP hydrolysis coupled proton transport
ATPase activity
ER to Golgi vesicle−mediated transport
insulin receptor signaling pathway
phagosome maturation
aminoacyl−tRNA ligase activity
autophagy
metabolic process
clathrin−mediated endocytosis
proton−transporting ATPase activity, rotational mechanism
transcription from RNA polymerase II promoter
translation initiation factor activity
locomotory behavior
cellular response to hypoxia
energy reserve metabolic process
enzyme regulator activity
interaction with host
transferrin transport
integrin−mediated signaling pathway
multicellular organismal development
cell cycle
neuron projection development
osteoblast differentiation
mitotic nuclear division
SRP−dependent cotranslational protein targeting to membrane
nuclear−transcribed mRNA catabolic process, nonsense−mediated decay
translational initiation
viral transcription
translational termination
nucleobase−containing small molecule metabolic process
in utero embryonic development
protein heterooligomerization
negative regulation of transcription, DNA−templated
T cell receptor signaling pathway
T cell costimulation
antigen processing and presentation of exogenous peptide antigen via MHC class II
protein dephosphorylation
hydrolase activity
cellular lipid metabolic process
post−translational protein modification
ubiquitin−dependent protein catabolic process
cellular nitrogen compound metabolic process
threonine−type endopeptidase activity
antigen processing and presentation of exogenous peptide antigen via MHC class I, TAP−dependent
antigen processing and presentation of exogenous peptide antigen via MHC class I
proteolysis
oxidoreductase activity
phospholipase inhibitor activity
translation
signal transducer activity
cell adhesion
GTP catabolic process
cell proliferation
cell redox homeostasis

Figure S14

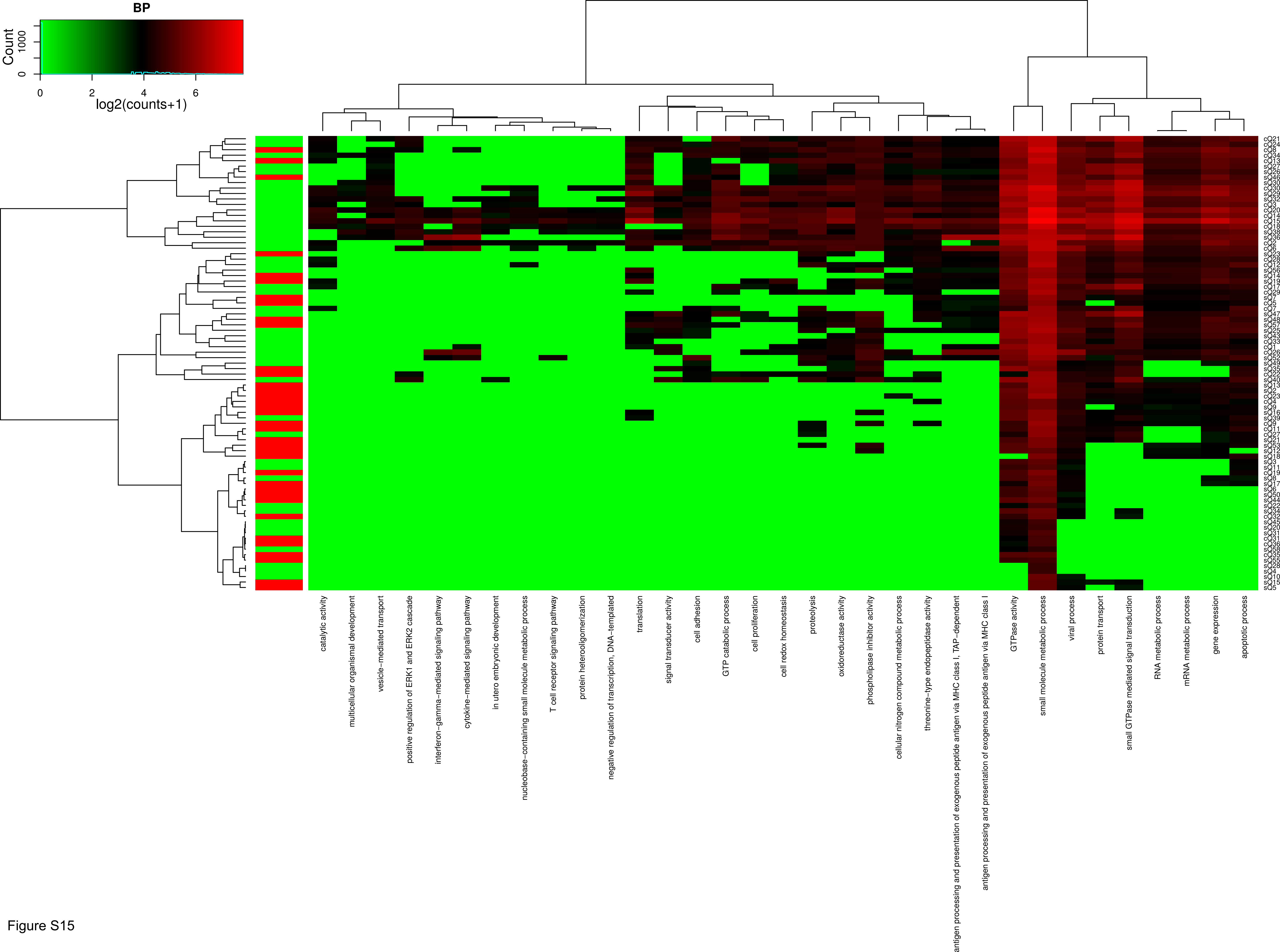
BP
Count
0
1000
0
2
4
6
log2(counts+1)
catalytic activity
multicellular organismal development
vesicle−mediated transport
positive regulation of ERK1 and ERK2 cascade
interferon−gamma−mediated signaling pathway
cytokine−mediated signaling pathway
in utero embryonic development
nucleobase−containing small molecule metabolic process
T cell receptor signaling pathway
protein heterooligomerization
negative regulation of transcription, DNA−templated
translation
signal transducer activity
cell adhesion
GTP catabolic process
cell proliferation
cell redox homeostasis
proteolysis
oxidoreductase activity
phospholipase inhibitor activity
cellular nitrogen compound metabolic process
threonine−type endopeptidase activity
antigen processing and presentation of exogenous peptide antigen via MHC class I, TAP−dependent
antigen processing and presentation of exogenous peptide antigen via MHC class I
GTPase activity
small molecule metabolic process
viral process
protein transport
small GTPase mediated signal transduction
RNA metabolic process
mRNA metabolic process
gene expression
apoptotic process

Figure S15

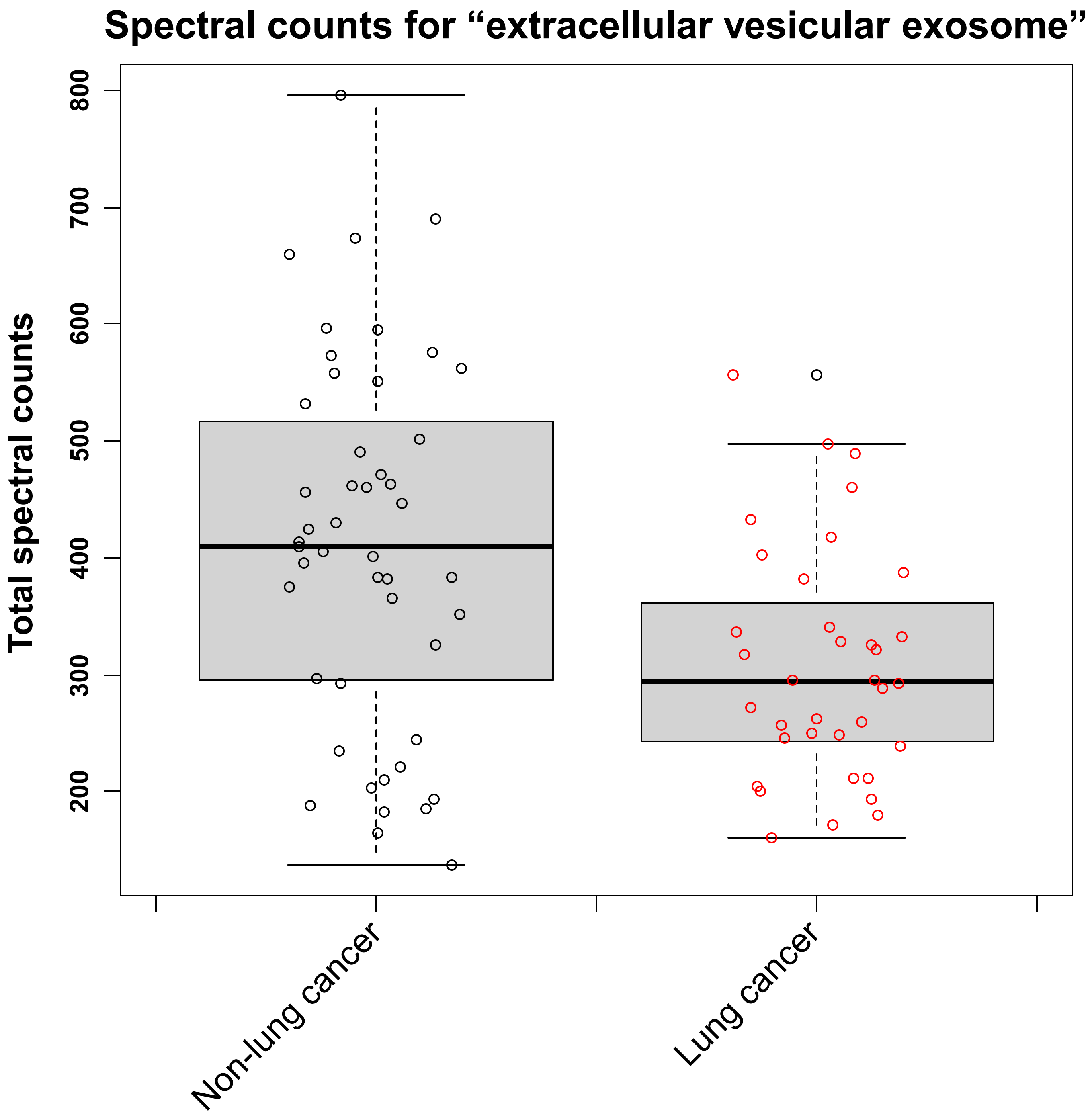

Spectral counts for “extracellular vesicular exosome”
Total spectral counts
800
700
600
500
400
300
200
Non-lung cancer
Lung cancer

Figure S16

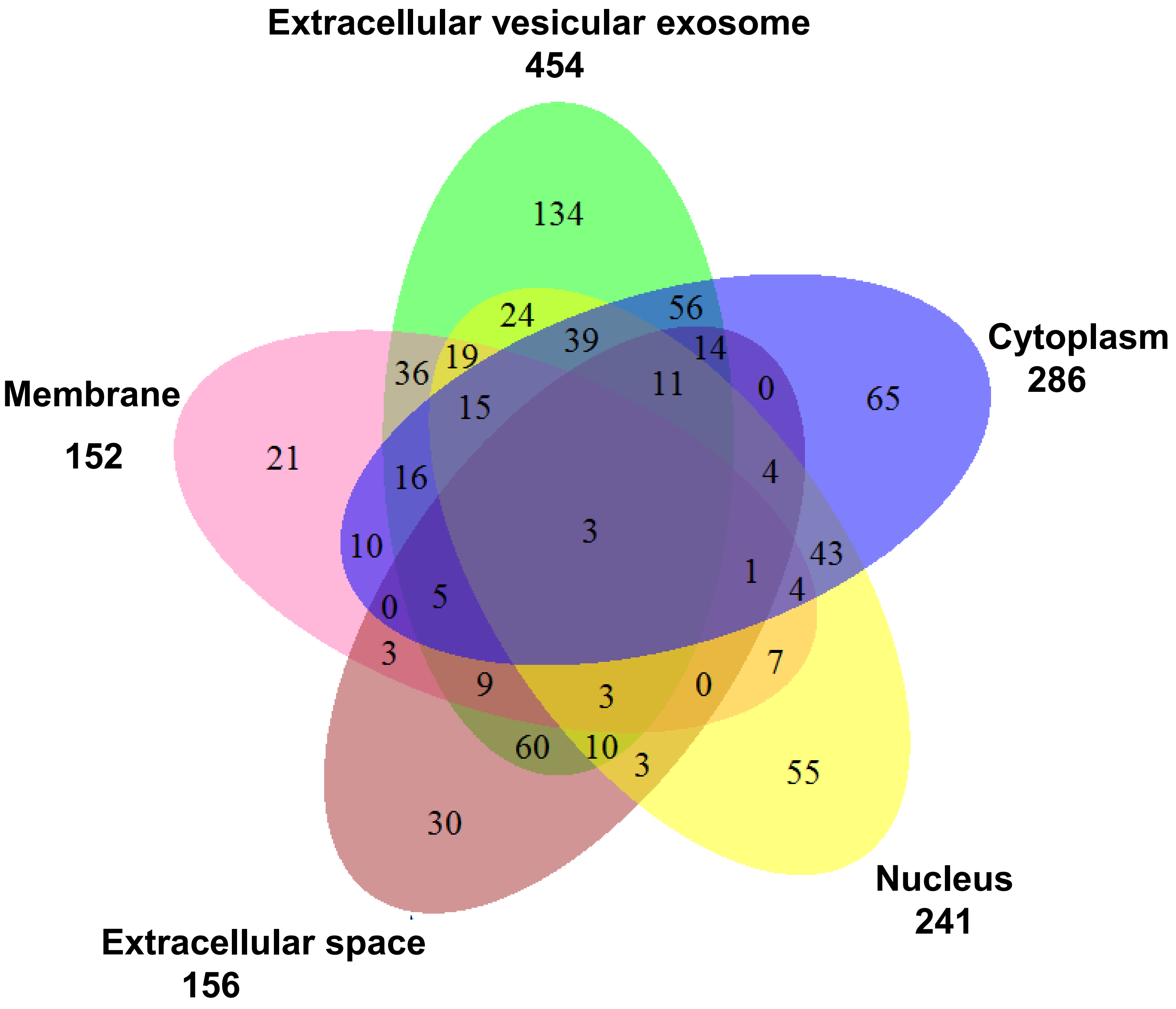
Extracellular vesicular exosome
454
Cytoplasm
286
Membrane
152
Nucleus
241
Extracellular space
156
134
24
56
39
19
36
14
11
0
15
65
21
16
4
3
10
43
1
4
5
0
3
7
9
3
0
60
10
3
55
30

Figure S17

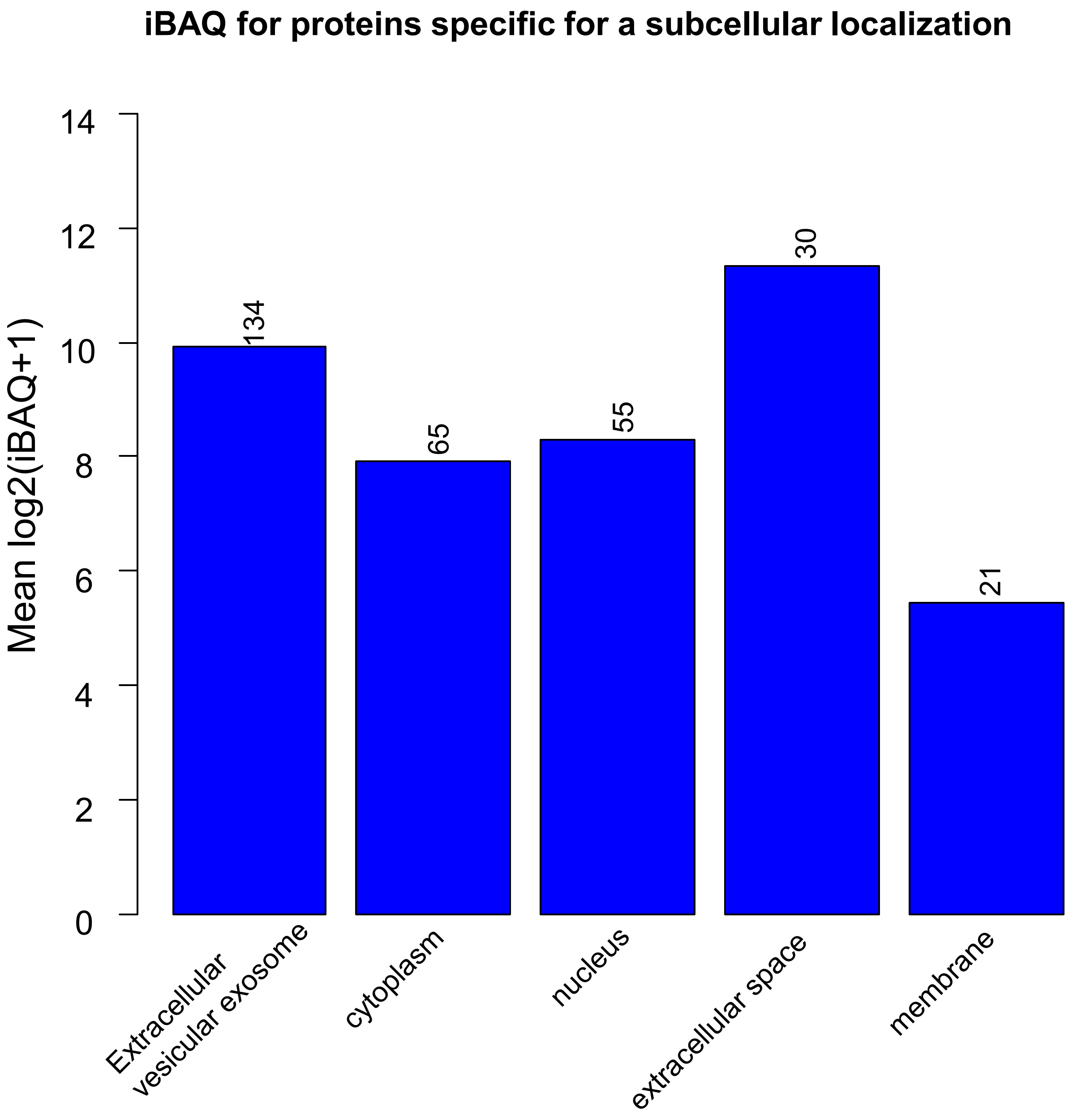
iBAQ for proteins specific for a subcellular localization
Mean log2(iBAQ+1)
0
2
4
6
8
10
12
14
134
65
55
30
21
Extracellular vesicular exosome
cytoplasm
nucleus
extracellular space
membrane

Figure S18

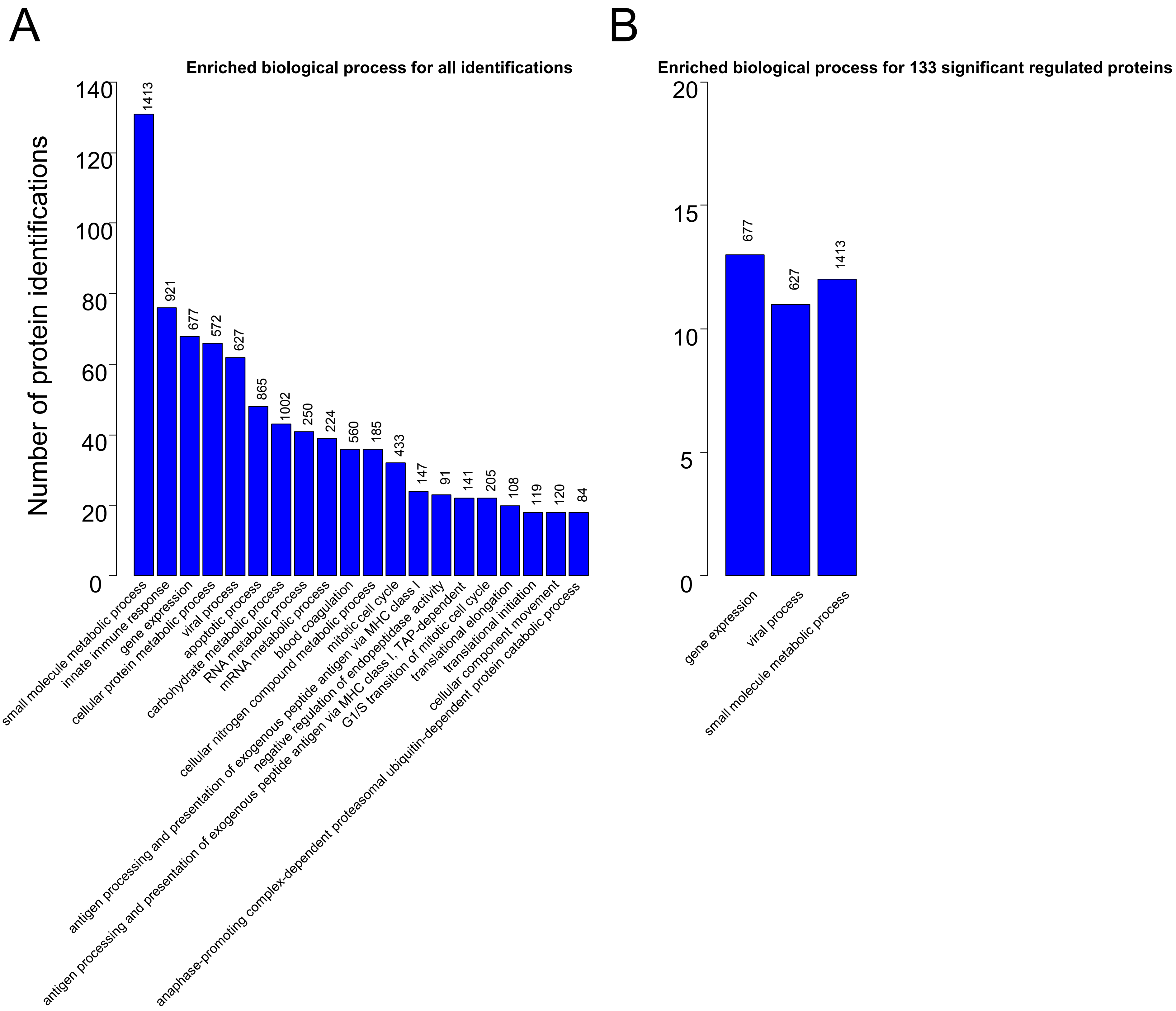
A
Enriched biological process for all identifications
Number of protein identifications
0
20
40
60
80
100
120
140
1413
921
677
572
627
865
1002
250
224
560
185
433
147
91
141
205
108
119
120
84
small molecule metabolic process
innate immune response
gene expression
cellular protein metabolic process
viral process
apoptotic process
carbohydrate metabolic process
RNA metabolic process
mRNA metabolic process
blood coagulation
cellular nitrogen compound metabolic process
mitotic cell cycle
antigen processing and presentation of exogenous peptide antigen via MHC class I
negative regulation of endopeptidase activity
antigen processing and presentation of exogenous peptide antigen via MHC class I, TAP-dependent
G1/S transition of mitotic cell cycle
translational elongation
translational initiation
cellular component movement
anaphase-promoting complex-dependent proteasomal ubiquitin-dependent protein catabolic process
B
Enriched biological process for 133 significant regulated proteins
0
5
10
15
20
677
627
1413
gene expression
viral process
small molecule metabolic process

Figure S19

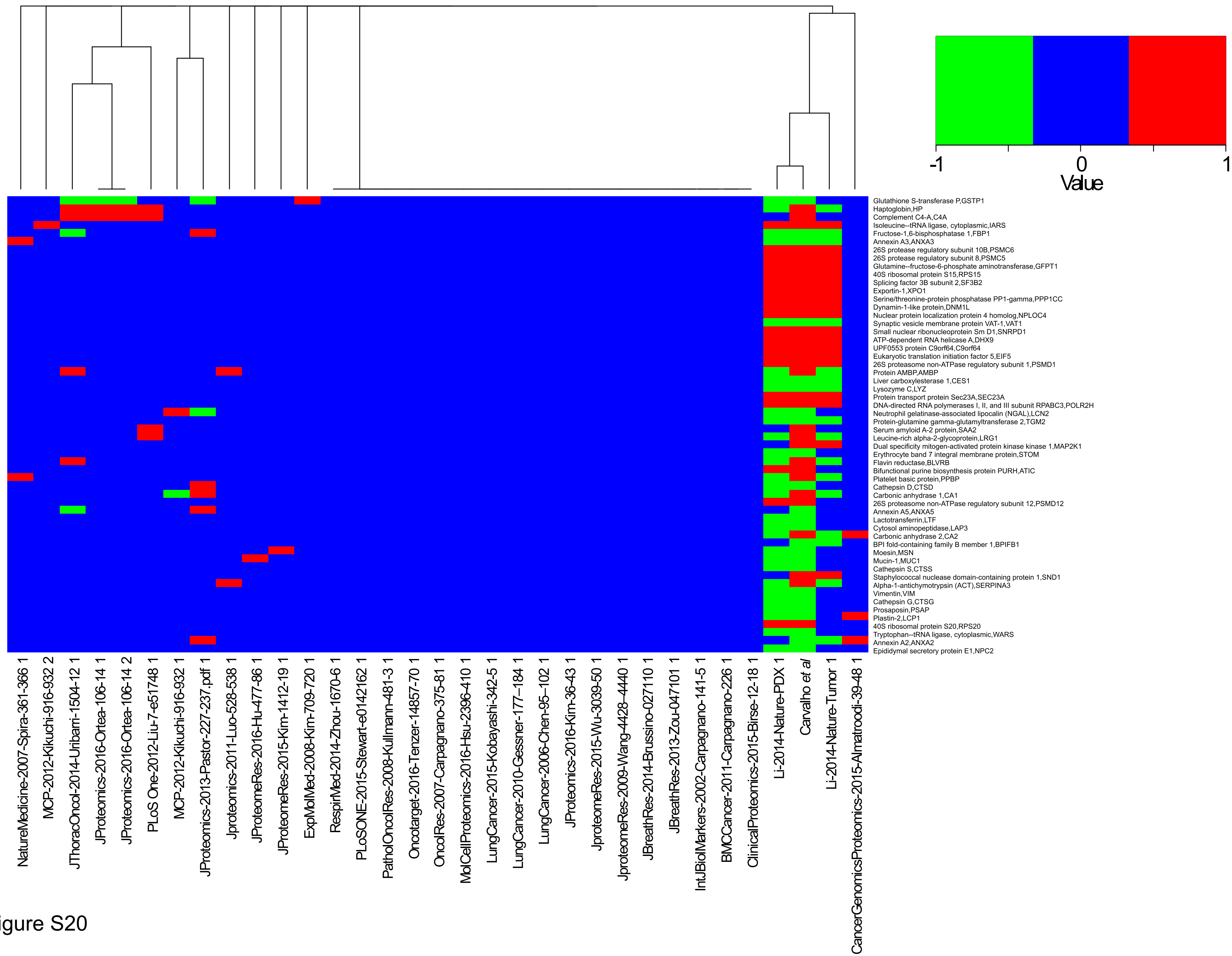

Value
-1
0
1
Glutathione S-transferase P,GSTP1
Haptoglobin,HP
Complement C4-A,C4A
Isoleucine--tRNA ligase, cytoplasmic,IARS
Fructose-1,6-bisphosphatase 1,FBP1
Annexin A3,ANXA3
26S protease regulatory subunit 10B,PSMC6
26S protease regulatory subunit 8,PSMC5
Glutamine--fructose-6-phosphate aminotransferase,GFPT1
40S ribosomal protein S15,RPS15
Splicing factor 3B subunit 2,SF3B2
Exportin-1,XPO1
Serine/threonine-protein phosphatase PP1-gamma,PPP1CC
Dynamin-1-like protein,DNM1L
Nuclear protein localization protein 4 homolog,NPLOC4
Synaptic vesicle membrane protein VAT-1,VAT1
Small nuclear ribonucleoprotein Sm D1,SNRPD1
ATP-dependent RNA helicase A,DHX9
UPF0553 protein C9orf64,C9orf64
Eukaryotic translation initiation factor 5,EIF5
26S proteasome non-ATPase regulatory subunit 1,PSMD1
Protein AMBP,AMBP
Liver carboxylesterase 1,CES1
Lysozyme C,LYZ
Protein transport protein Sec23A,SEC23A
DNA-directed RNA polymerases I, II, and III subunit RPABC3,POLR2H
Neutrophil gelatinase-associated lipocalin (NGAL),LCN2
Protein-glutamine gamma-glutamyltransferase 2,TGM2
Serum amyloid A-2 protein,SAA2
Leucine-rich alpha-2-glycoprotein,LRG1
Dual specificity mitogen-activated protein kinase kinase 1,MAP2K1
Erythrocyte band 7 integral membrane protein,STOM
Flavin reductase,BLVRB
Bifunctional purine biosynthesis protein PURH,ATIC
Platelet basic protein,PPBP
Cathepsin D,CTSD
Carbonic anhydrase 1,CA1
26S proteasome non-ATPase regulatory subunit 12,PSMD12
Annexin A5,ANXA5
Lactotransferrin,LTF
Cytosol aminopeptidase,LAP3
Carbonic anhydrase 2,CA2
BPI fold-containing family B member 1,BPIFB1
Moesin,MSN
Mucin-1,MUC1
Cathepsin S,CTSS
Staphylococcal nuclease domain-containing protein 1,SND1
Alpha-1-antichymotrypsin (ACT),SERPINA3
Vimentin,VIM
Cathepsin G,CTSG
Prosaposin,PSAP
Plastin-2,LCP1
40S ribosomal protein S20,RPS20
Tryptophan--tRNA ligase, cytoplasmic,WARS
Annexin A2,ANXA2
Epididymal secretory protein E1,NPC2
NatureMedicine-2007-Spira-361-366 1
MCP-2012-Kikuchi-916-932 2
JThoracOncol-2014-Uribarri-1504-12 1
JProteomics-2016-Ortea-106-14 1
JProteomics-2016-Ortea-106-14 2
PLoS One-2012-Liu-7-e51748 1
MCP-2012-Kikuchi-916-932 1
JProteomics-2013-Pastor-227-237.pdf 1
Jproteomics-2011-Luo-528-538 1
JProteomeRes-2016-Hu-477-86 1
JProteomeRes-2015-Kim-1412-19 1
ExpMolMed-2008-Kim-709-720 1
RespirMed-2014-Zhou-1670-6 1
PLoSONE-2015-Stewart-e0142162 1
PatholOncolRes-2008-Kullmann-481-3 1
Oncotarget-2016-Tenzer-14857-70 1
OncolRes-2007-Carpagnano-375-81 1
MolCellProteomics-2016-Hsu-2396-410 1
LungCancer-2015-Kobayashi-342-5 1
LungCancer-2010-Gessner-177–184 1
LungCancer-2006-Chen-95–102 1
JProteomics-2016-Kim-36-43 1
JproteomeRes-2015-Wu-3039-50 1
JproteomeRes-2009-Wang-4428-4440 1
JBreathRes-2014-Brussino-027110 1
JBreathRes-2013-Zou-047101 1
IntJBiolMarkers-2002-Carpagnano-141-5 1
BMCCancer-2011-Carpagnano-226 1
ClinicalProteomics-2015-Birse-12-18 1
Li-2014-Nature-PDX 1
Carvalho *et al*
Li-2014-Nature-Tumor 1
CancerGenomicsProteomics-2015-Almatroodi-39-48 1

Figure S20

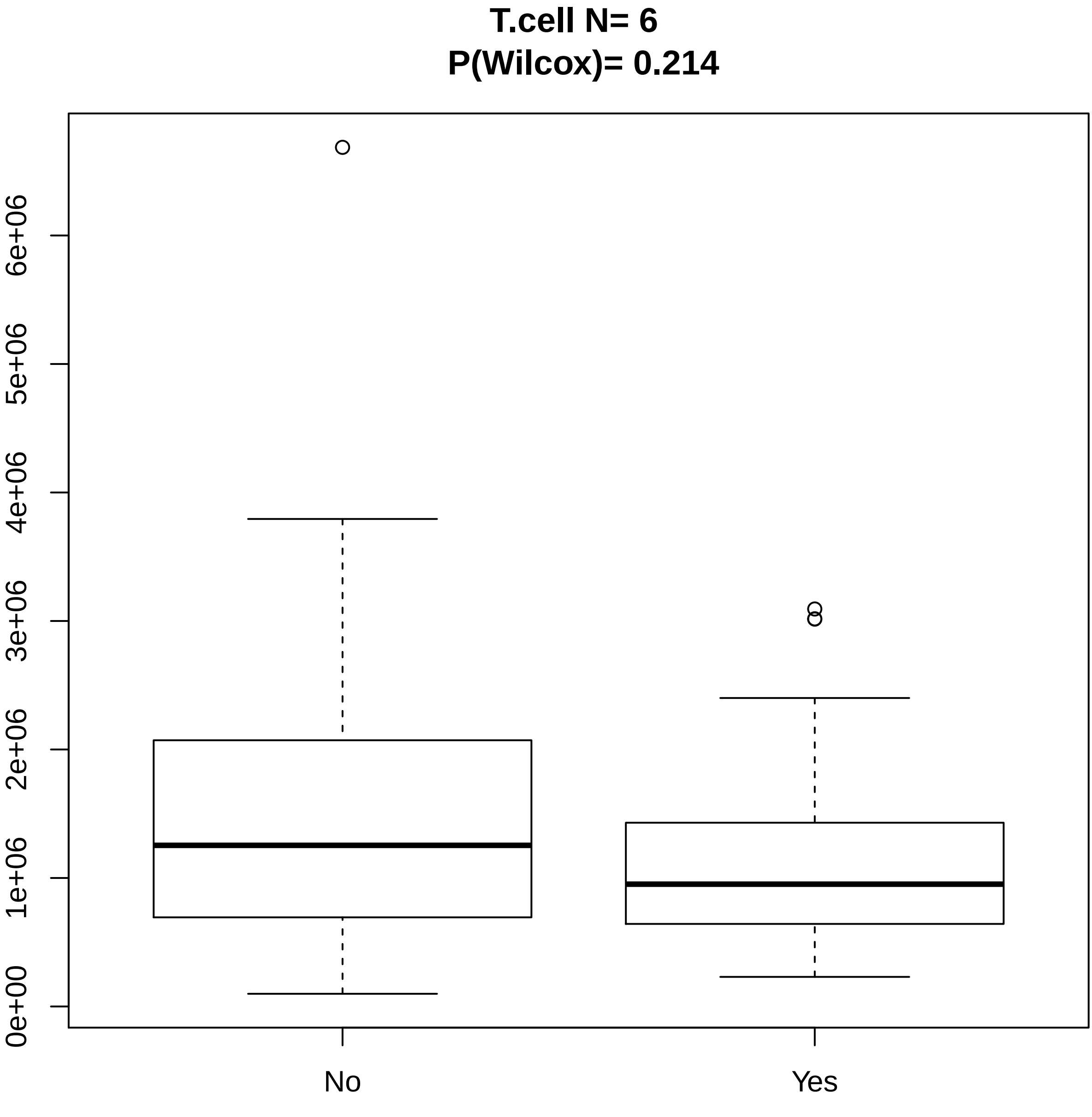
T.cell N= 6
P(Wilcox)= 0.214
0e+00
1e+06
2e+06
3e+06
4e+06
5e+06
6e+06
No
Yes

Figure S21

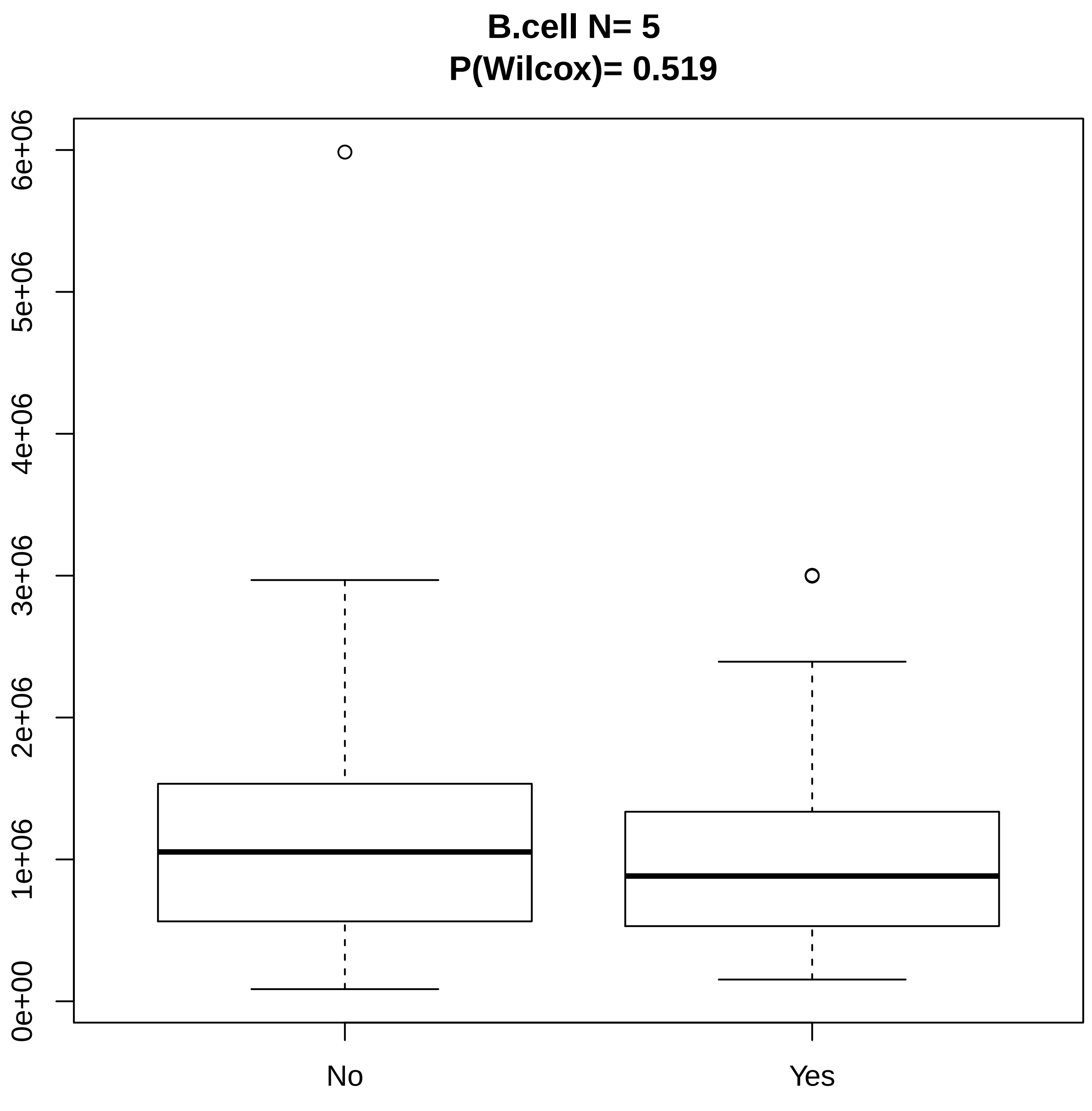
B.cell N= 5
P(Wilcox)= 0.519
0e+00
1e+06
2e+06
3e+06
4e+06
5e+06
6e+06
No
Yes

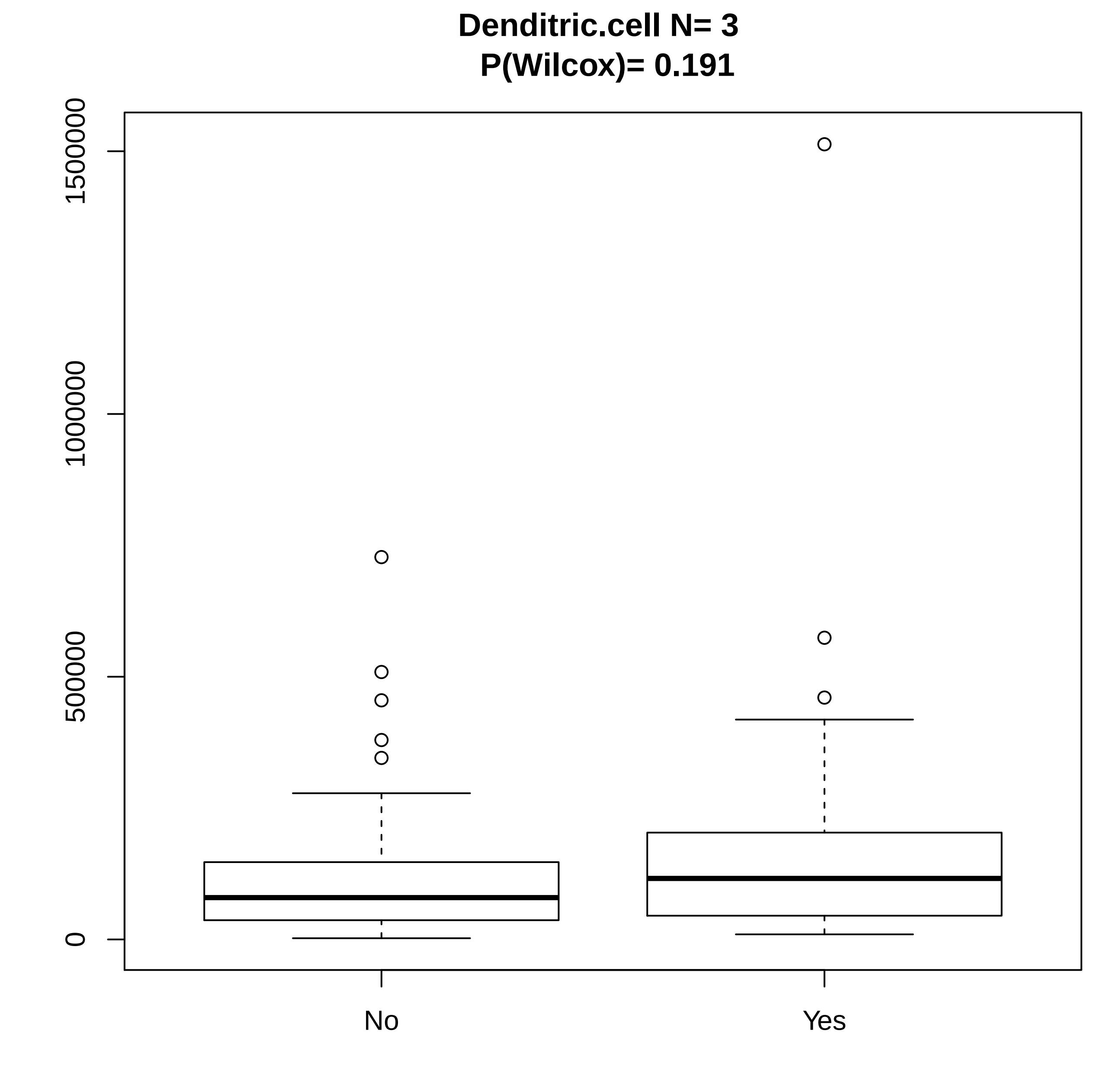
Denditric.cell N= 3
P(Wilcox)= 0.191
0
500000
1000000
1500000
No
Yes

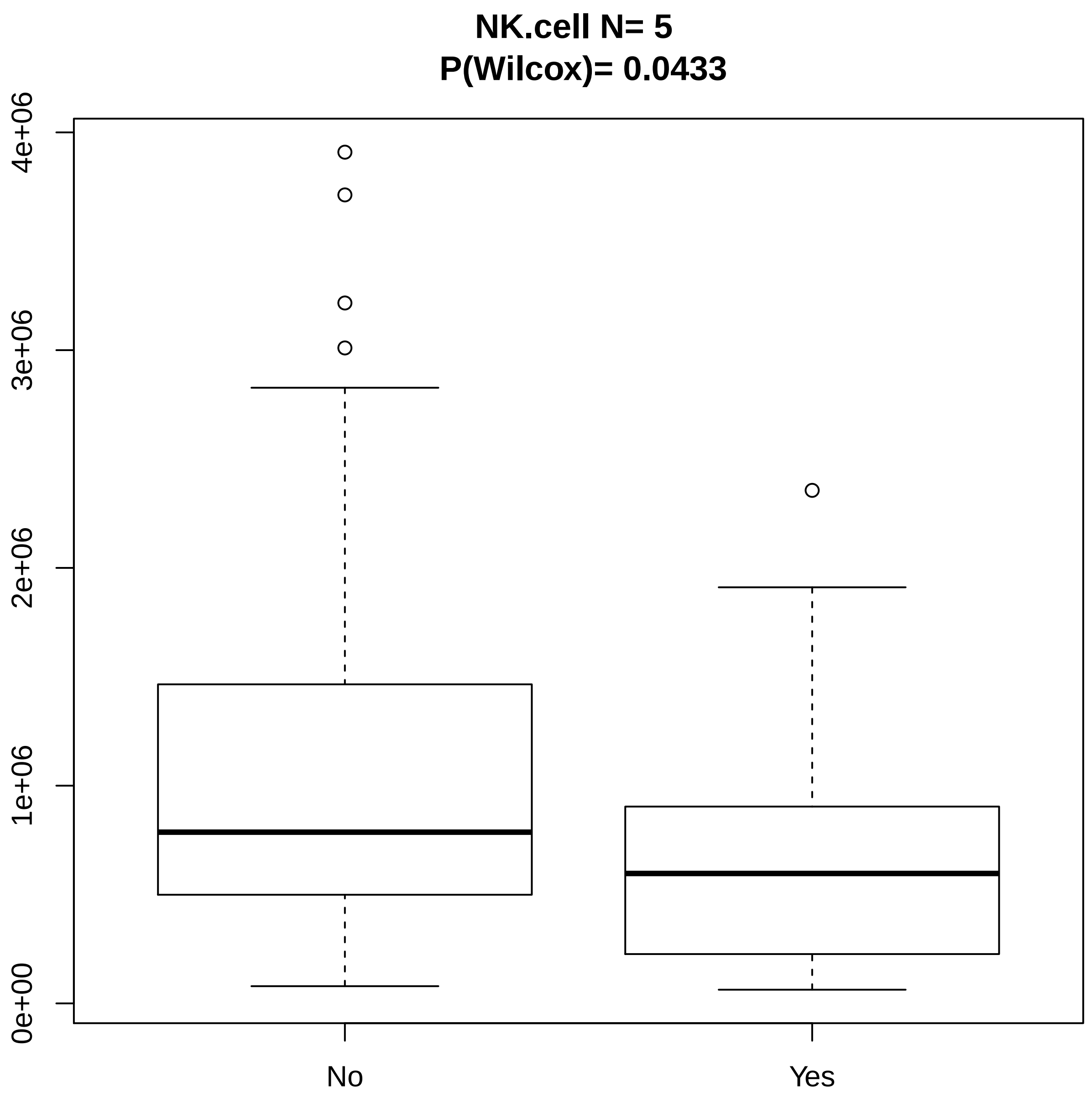
NK.cell N= 5
P(Wilcox)= 0.0433
0e+00
1e+06
2e+06
3e+06
4e+06
No
Yes

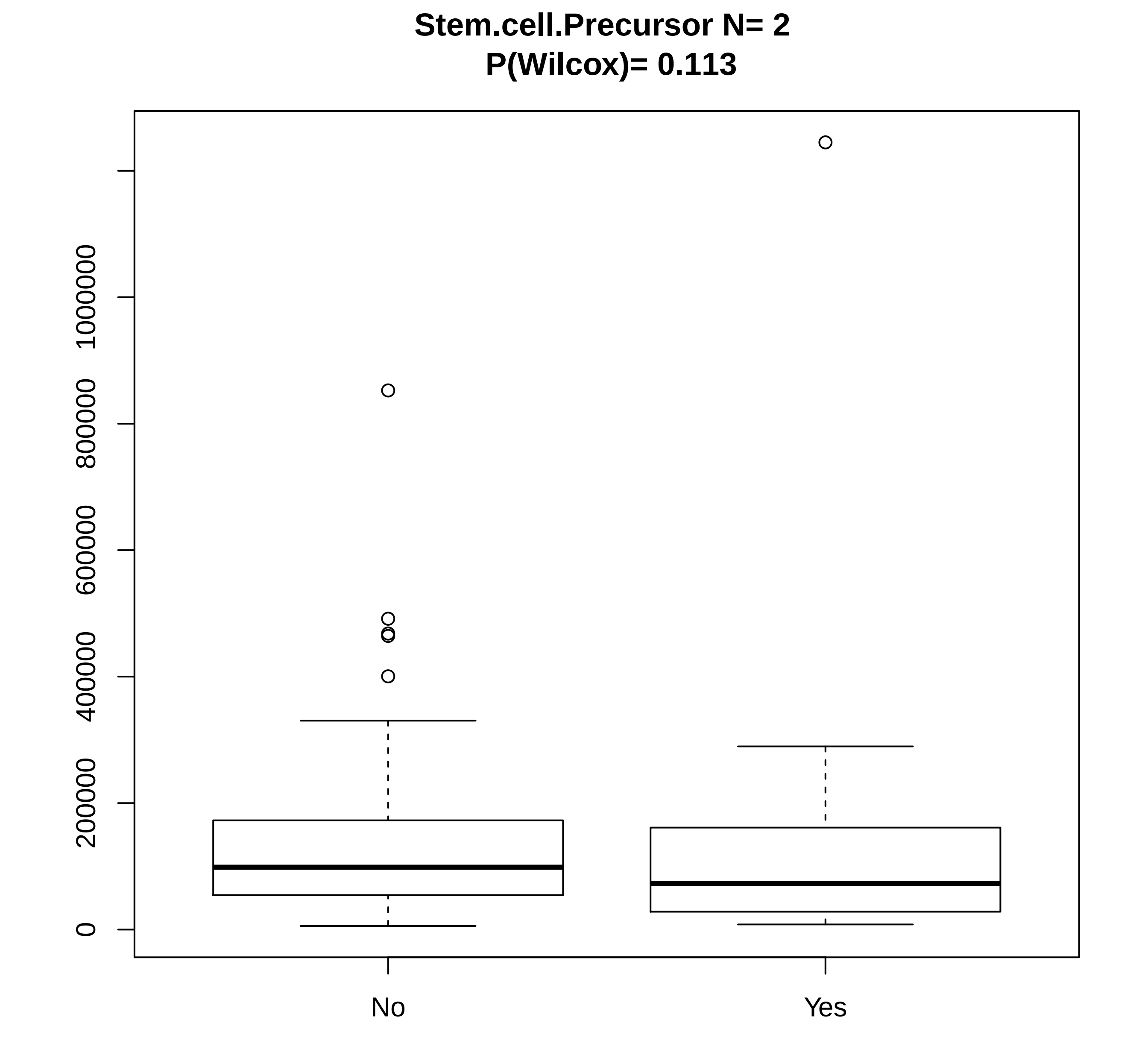
Stem.cell.Precursor N= 2
P(Wilcox)= 0.113
0
200000
400000
600000
800000
1000000
No
Yes

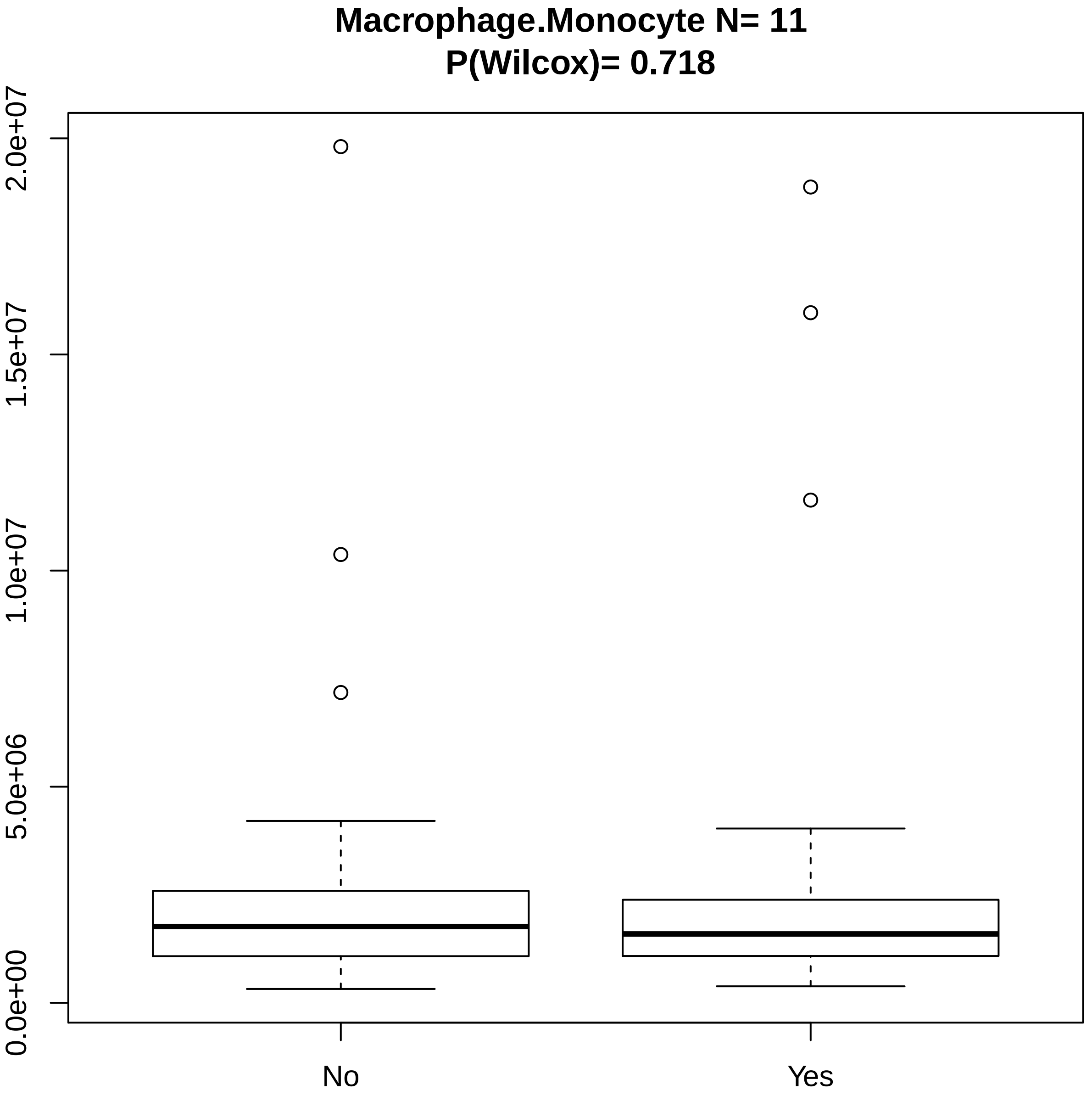
Macrophage.Monocyte N= 11
P(Wilcox)= 0.718
0.0e+00
5.0e+06
1.0e+07
1.5e+07
2.0e+07
No
Yes

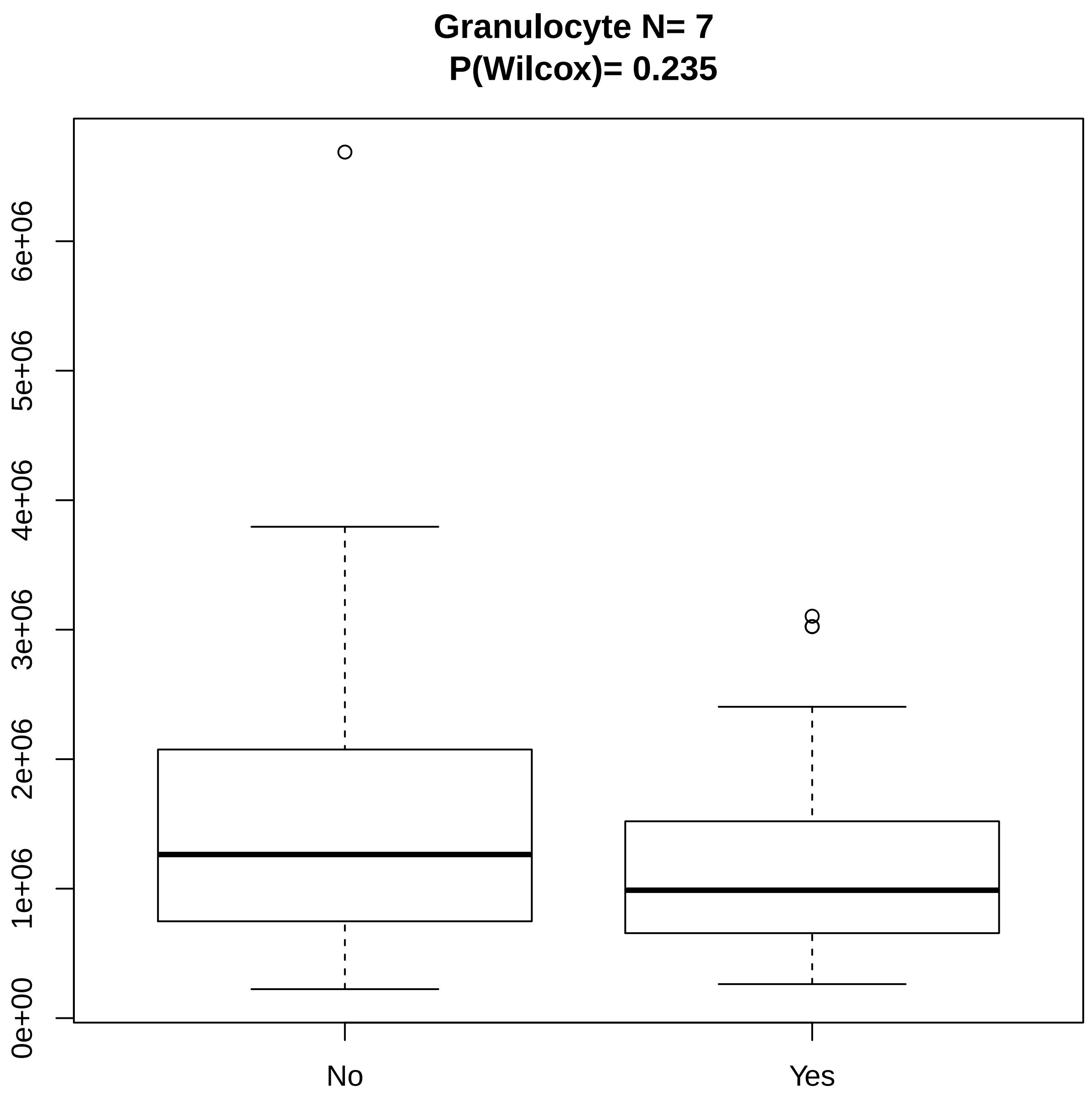
Granulocyte N= 7
P(Wilcox)= 0.235
0e+00
1e+06
2e+06
3e+06
4e+06
5e+06
6e+06
No
Yes

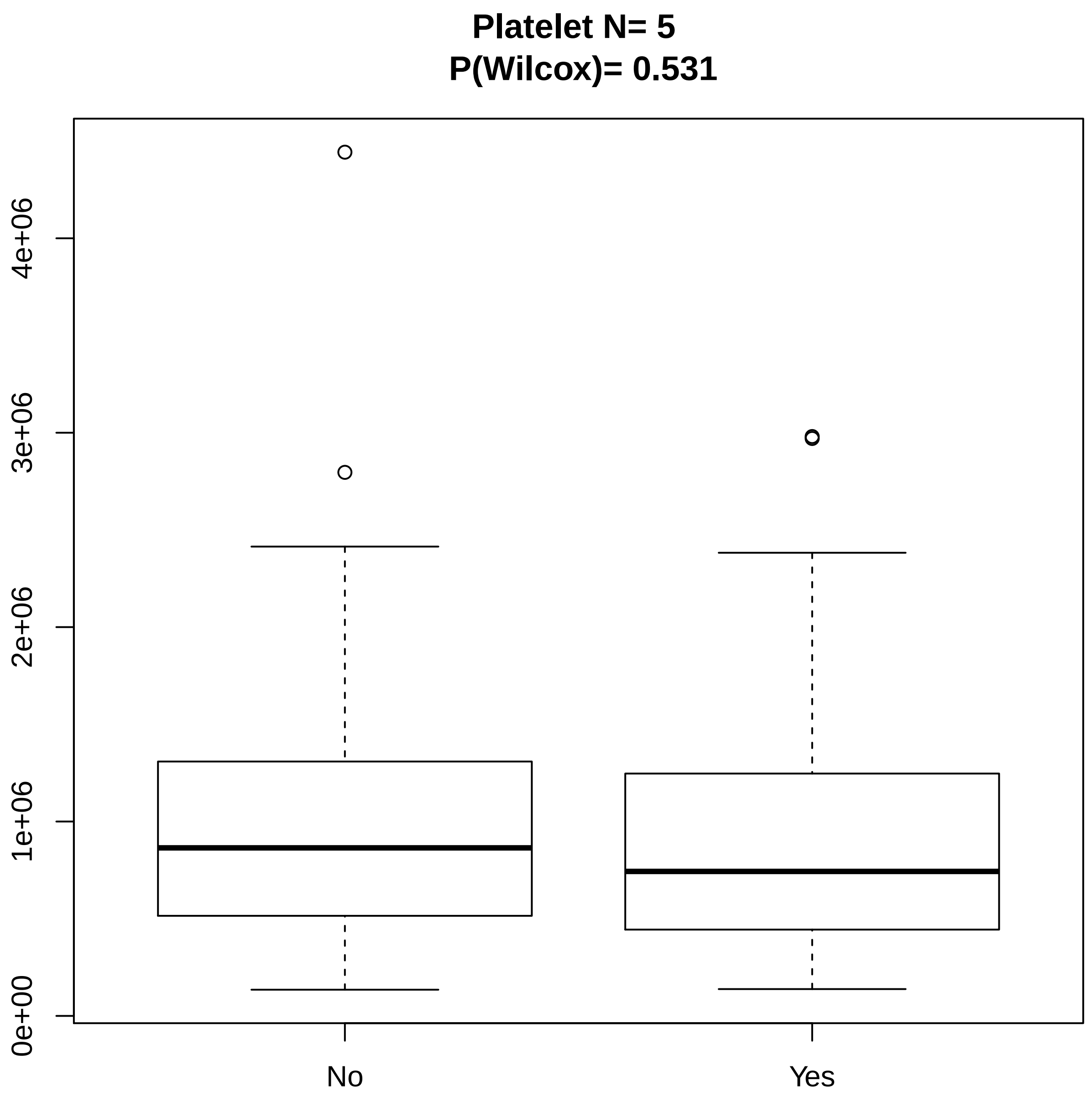
Platelet N= 5
P(Wilcox)= 0.531
0e+00
1e+06
2e+06
3e+06
4e+06
No
Yes

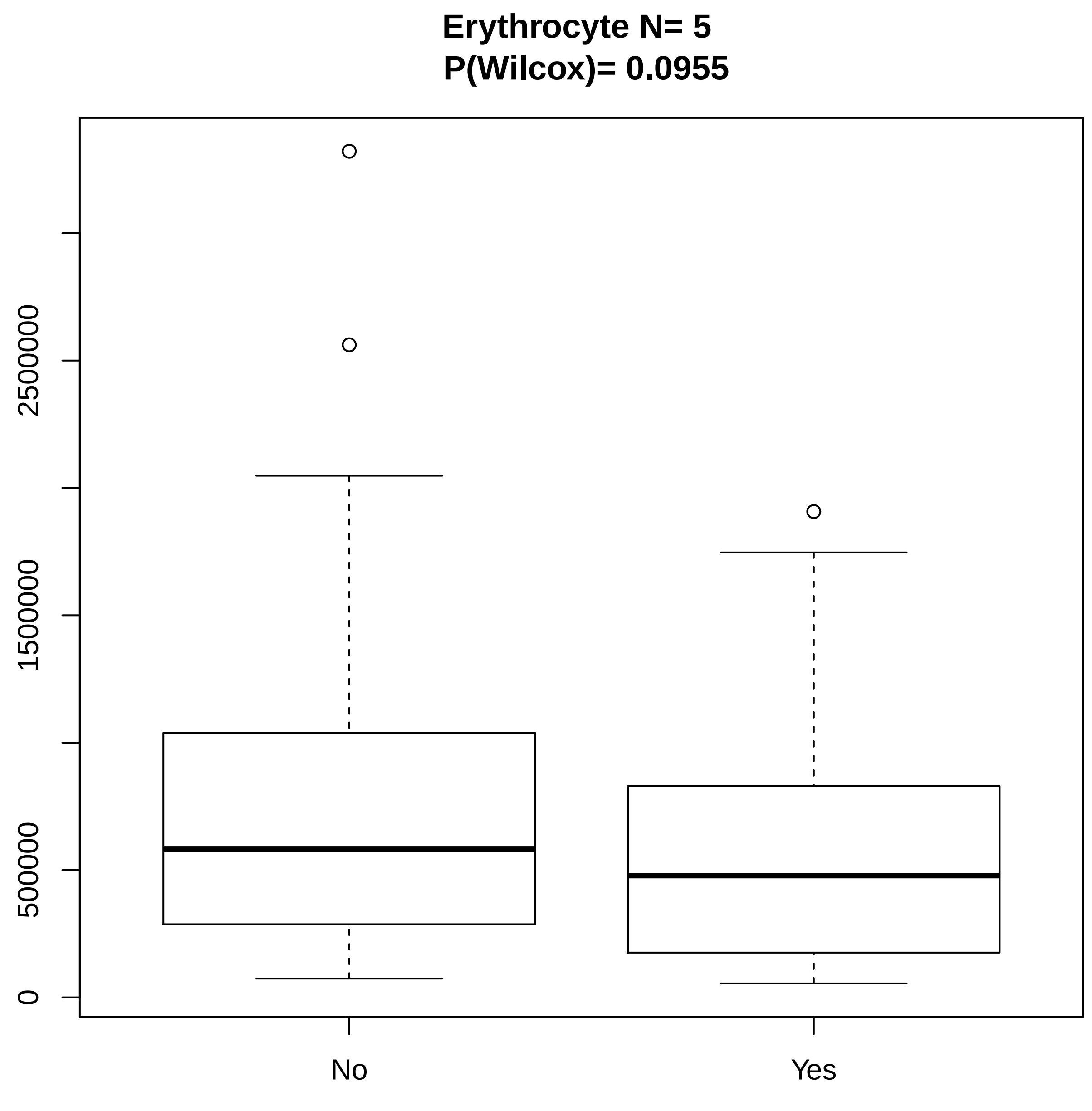
Erythrocyte N= 5
P(Wilcox)= 0.0955
0
500000
1500000
2500000
No
Yes

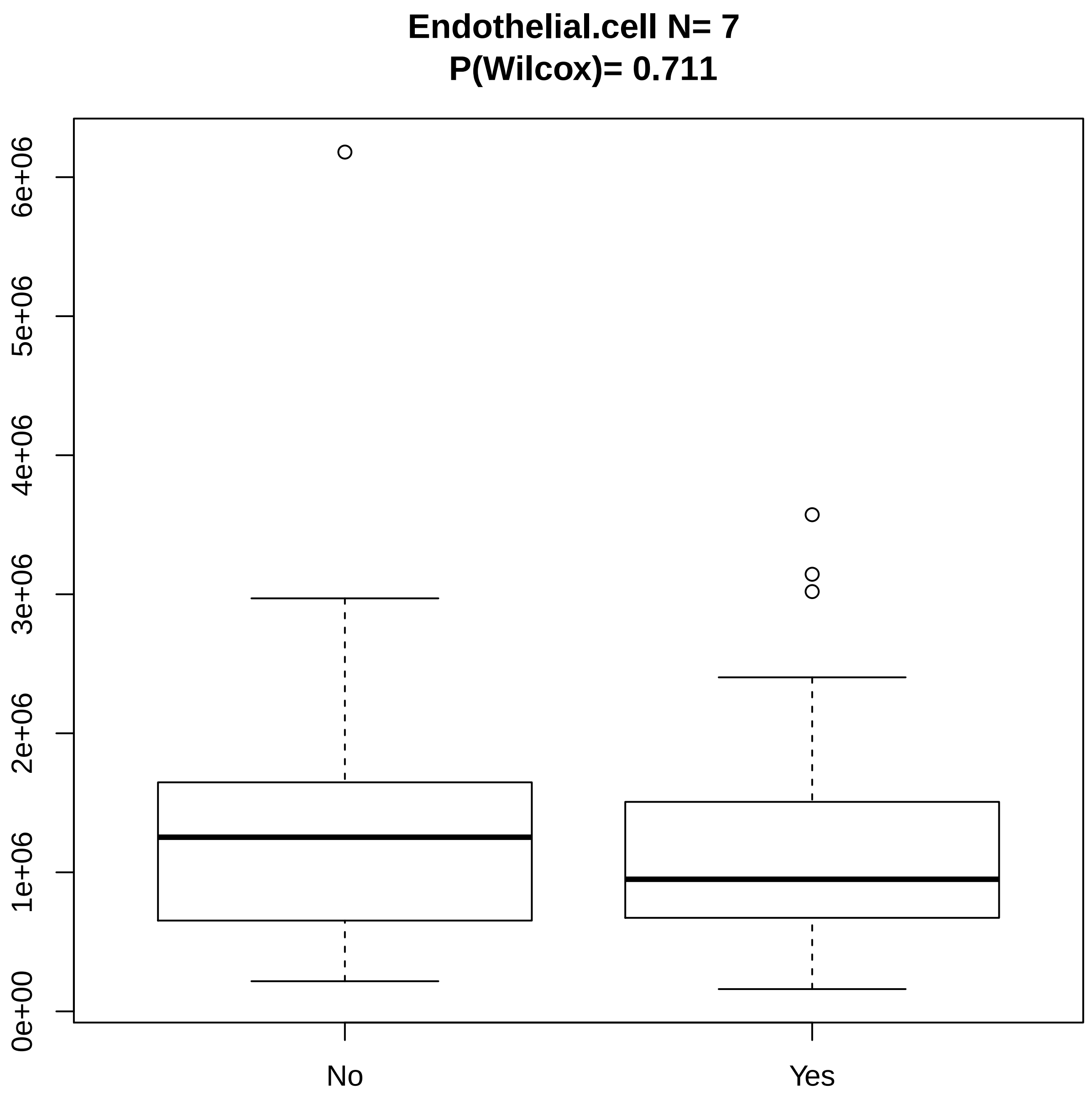
Endothelial.cell N= 7
P(Wilcox)= 0.711
0e+00
1e+06
2e+06
3e+06
4e+06
5e+06
6e+06
No
Yes

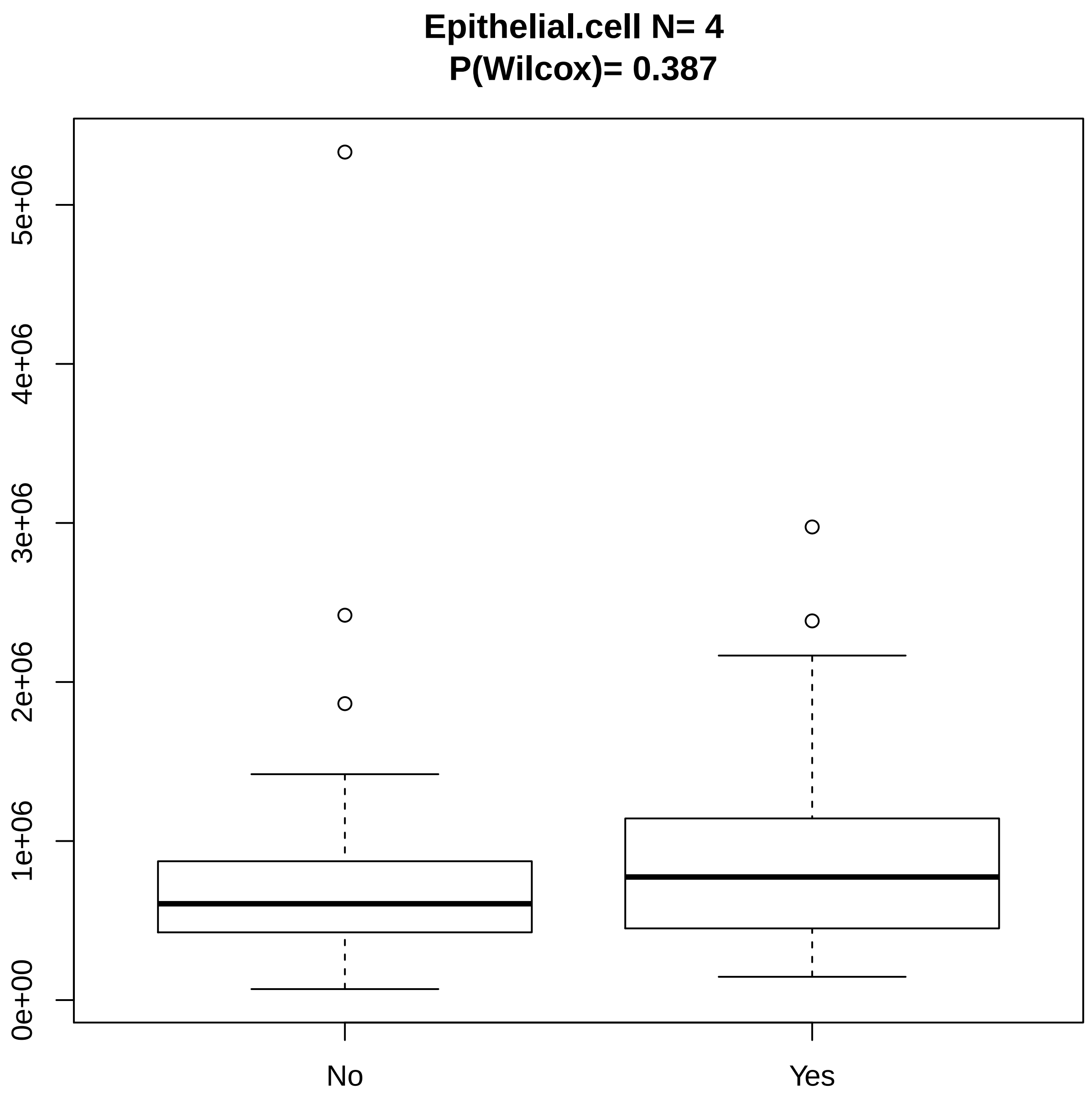
Epithelial.cell N= 4
P(Wilcox)= 0.387
0e+00
1e+06
2e+06
3e+06
4e+06
5e+06
No
Yes

References for table S1

1 Almatroodi, S. A., McDonald, C. F., Collins, A. L., Darby, I. A. & Pouniotis, D. S. Quantitative proteomics of bronchoalveolar lavage fluid in lung adenocarcinoma. *Cancer genomics & proteomics* **12**, 39-48 (2015).

2 Ortea, I., Rodriguez-Ariza, A., Chicano-Galvez, E., Arenas Vacas, M. S. & Jurado Gamez, B. Discovery of potential protein biomarkers of lung adenocarcinoma in bronchoalveolar lavage fluid by SWATH MS data-independent acquisition and targeted data extraction. *Journal of proteomics* **138**, 106-114, doi:10.1016/j.jprot.2016.02.010 (2016).

3 Uribarri, M. *et al.* A new biomarker panel in bronchoalveolar lavage for an improved lung cancer diagnosis. *Journal of thoracic oncology : official publication of the International Association for the Study of Lung Cancer* **9**, 1504-1512, doi:10.1097/JTO.0000000000000282 (2014).

4 Pastor, M. D. *et al.* Identification of proteomic signatures associated with lung cancer and COPD. *Journal of proteomics* **89**, 227-237, doi:10.1016/j.jprot.2013.04.037 (2013).

5 Kim, Y. J. *et al.* Verification of the biomarker candidates for non-small-cell lung cancer using a targeted proteomics approach. *Journal of proteome research* **14**, 1412-1419, doi:10.1021/pr5010828 (2015).

6 Wu, H. Y. *et al.* Qualification and Verification of Serological Biomarker Candidates for Lung Adenocarcinoma by Targeted Mass Spectrometry. *Journal of proteome research* **14**, 3039-3050, doi:10.1021/pr501195t (2015).

7 Wang, C. L. *et al.* Discovery of retinoblastoma-associated binding protein 46 as a novel prognostic marker for distant metastasis in nonsmall cell lung cancer by combined analysis of cancer cell secretome and pleural effusion proteome. *Journal of proteome research* **8**, 4428-4440, doi:10.1021/pr900160h (2009).

8 Luo, X. *et al.* A high-quality secretome of A549 cells aided the discovery of C4b-binding protein as a novel serum biomarker for non-small cell lung cancer. *Journal of proteomics* **74**, 528-538, doi:10.1016/j.jprot.2011.01.011 (2011).

9 Chen, Y. *et al.* Elevation of serum l-lactate dehydrogenase B correlated with the clinical stage of lung cancer. *Lung Cancer* **54**, 95-102, doi:10.1016/j.lungcan.2006.06.014 (2006).

10 Liu, Y. *et al.* Integrative proteomics and tissue microarray profiling indicate the association between overexpressed serum proteins and non-small cell lung cancer. *PloS one* **7**, e51748, doi:10.1371/journal.pone.0051748 (2012).

11 Birse, C. E. *et al.* Blood-based lung cancer biomarkers identified through proteomic discovery in cancer tissues, cell lines and conditioned medium. *Clinical proteomics* **12**, 18, doi:10.1186/s12014-015-9090-9 (2015).

12 Kobayashi, M. *et al.* Calnexin is a novel sero-diagnostic marker for lung cancer. *Lung Cancer* **90**, 342-345, doi:10.1016/j.lungcan.2015.08.015 (2015).

13 Kim, Y. I. *et al.* Meta-markers for the differential diagnosis of lung cancer and lung disease. *Journal of proteomics* **148**, 36-43, doi:10.1016/j.jprot.2016.04.052 (2016).

14 Kikuchi, T. *et al.* In-depth proteomic analysis of nonsmall cell lung cancer to discover molecular targets and candidate biomarkers. *Molecular & cellular proteomics : MCP* **11**, 916-932, doi:10.1074/mcp.M111.015370 (2012).

15 Tenzer, S. *et al.* Integrated quantitative proteomic and transcriptomic analysis of lung tumor and control tissue: a lung cancer showcase. *Oncotarget* **7**, 14857-14870, doi:10.18632/oncotarget.7562 (2016).

16 Li, L. *et al.* Integrated omic analysis of lung cancer reveals metabolism proteome signatures with prognostic impact. *Nature communications* **5**, 5469, doi:10.1038/ncomms6469 (2014).
17 Stewart, P. A. *et al.* A Pilot Proteogenomic Study with Data Integration Identifies MCT1 and GLUT1 as Prognostic Markers in Lung Adenocarcinoma. *PloS one* **10**, e0142162, doi:10.1371/journal.pone.0142162 (2015).
18 Hsu, C. H. *et al.* Identification and Characterization of Potential Biomarkers by Quantitative Tissue Proteomics of Primary Lung Adenocarcinoma. *Molecular & cellular proteomics : MCP* **15**, 2396-2410, doi:10.1074/mcp.M115.057026 (2016).
19 Kim, J. E., Koo, K. H., Kim, Y. H., Sohn, J. & Park, Y. G. Identification of potential lung cancer biomarkers using an in vitro carcinogenesis model. *Experimental & molecular medicine* **40**, 709-720, doi:10.3858/emm.2008.40.6.709 (2008).
20 Hu, R. *et al.* Quantitative Secretomic Analysis Identifies Extracellular Protein Factors That Modulate the Metastatic Phenotype of Non-Small Cell Lung Cancer. *Journal of proteome research* **15**, 477-486, doi:10.1021/acs.jproteome.5b00819 (2016).
21 Spira, A. *et al.* Airway epithelial gene expression in the diagnostic evaluation of smokers with suspect lung cancer. *Nature medicine* **13**, 361-366, doi:10.1038/nm1556 (2007).
22 Brussino, L. *et al.* Inflammatory cytokines and VEGF measured in exhaled breath condensate are correlated with tumor mass in non-small cell lung cancer. *Journal of breath research* **8**, 027110, doi:10.1088/1752-7155/8/2/027110 (2014).
23 Carpagnano, G. E., Resta, O., Foschino-Barbaro, M. P., Gramiccioni, E. & Carpagnano, F. Interleukin-6 is increased in breath condensate of patients with non-small cell lung cancer. *The International journal of biological markers* **17**, 141-145 (2002).
24 Carpagnano, G. E. *et al.* IL-2, TNF-alpha, and leptin: local versus systemic concentrations in NSCLC patients. *Oncology research* **16**, 375-381 (2007).
25 Gessner, C. *et al.* Angiogenic markers in breath condensate identify non-small cell lung cancer. *Lung Cancer* **68**, 177-184, doi:10.1016/j.lungcan.2009.06.010 (2010).
26 Carpagnano, G. E. *et al.* Neutrophilic airways inflammation in lung cancer: the role of exhaled LTB-4 and IL-8. *BMC cancer* **11**, 226, doi:10.1186/1471-2407-11-226 (2011).
27 Kullmann, T., Barta, I., Csiszer, E., Antus, B. & Horvath, I. Differential cytokine pattern in the exhaled breath of patients with lung cancer. *Pathology oncology research : POR* **14**, 481-483, doi:10.1007/s12253-008-9046-8 (2008).
28 Zou, Y. *et al.* CEA, SCC and NSE levels in exhaled breath condensate--possible markers for early detection of lung cancer. *Journal of breath research* **7**, 047101, doi:10.1088/1752-7155/7/4/047101 (2013).
29 Carpagnano, G. E. *et al.* Cigarette smoke and increased COX-2 and survivin levels in exhaled breath condensate of lung cancer patients: how hot is the link? *Lung Cancer* **67**, 108-113, doi:10.1016/j.lungcan.2009.03.033 (2010).
30 Zhou, F. *et al.* Increased levels of exhaled sICAM1, sVCAM1, and sE-selectin in patients with non-small cell lung cancer. *Respiratory medicine* **108**, 1670-1676, doi:10.1016/j.rmed.2014.08.003 (2014).
